# An extensive morphological and molecular characterization of the neglected class Odontostomatea (Ciliophora)

**DOI:** 10.1007/s42995-026-00352-x

**Published:** 2026-03-12

**Authors:** Daniel Méndez-Sánchez, Ondřej Pomahač, Marek Valt, William A. Bourland, Ivan Čepička

**Affiliations:** Department of Zoology, Faculty of Science, Charles University, 128 00 Prague, Czech Republic

**Keywords:** Anoxic sediments, Discomorphellidae, Epalxellidae, Environmental sequences, Mylestomatidae, Phylogeny

## Abstract

Odontostomatid ciliates, known for over a century, were historically classified within various taxonomic groups of Ciliophora Doflein, 1901 until their reclassification into the class Odontostomatea. Despite the recognition of 25 valid species, most descriptions predate the advent of silver impregnation and sequencing methods. Consequently, many species were described based solely on observations of live specimens, leading to incomplete or ambiguous records. To date, redescriptions of only three species include 18S rRNA gene sequences data, and their evolutionary relationships remain unresolved. In this study, we investigated 32 populations representing 15 species—including three newly described—across the genera *Discomorphella*, *Epalxella*, *Limnomylestoma* gen. nov., *Mircalla* gen. nov., *Mylestoma*, *Pelodinium*, *Saprodinium*, and *Tostonella* gen. nov. Comprehensive analyses were conducted using in vivo microscopy, silver impregnation, and scanning electron microscopy. We also designed specific primers to amplify the partial 18S rRNA gene of various odontostomateans and retrieved additional 18S rRNA sequences from environmental metatranscriptomic and metagenomic datasets. This study represents the most extensive investigation of Odontostomatea to date, confirming the monophyly of the class by revealing the position of *Epalxella*, reconstructing its internal phylogeny, identifying two main odontostomatean lineages, and revealing its remarkable diversity.

**Supplementary Information:**

The online version contains supplementary material available at 10.1007/s42995-026-00352-x.

## Introduction

Until now, the taxonomy of the anaerobic ciliate class Odontostomatea Fernandes et al., 2018 has been little-studied by modern techniques, e.g., protargol impregnation, scanning electron microscopy, and gene sequencing. Odontostomateans are free-living anaerobic ciliates dwelling in anoxic/hypoxic freshwater, brackish water, or marine environments. Perhaps, their most outstanding characteristics include a complex morphology consisting of a rigid amour-like cortex equipped with spines, an epistomial fringe or frontal band, and a highly reduced somatic ciliature arising from cortical pits. Additionally, they possess mitochondrion-related organelles and prokaryotic endosymbionts (Fernandes et al. 2018; Jankowski 1964a, b; Kahl 1932a; Lynn 2008; Méndez-Sánchez et al. 2023; Paiva et al. 2017; Schrenk and Bardele 1991).

Since first discovery over a century ago, at least 25 species of odontostomateans have been described and classified into a single order, Odontostomatida Sawaya, 1940, three families: Discomorphellidae Corliss, 1960, Epalxellidae Corliss, 1960, and Mylestomatidae Kahl in Doflein & Reichenow, 1929, and six genera: *Atopodinium* Kahl, 1932, *Discomorphella* Corliss, 1960, *Epalxella* Corliss, 1960, *Mylestoma* Kahl, 1928, *Pelodinium* Lauterborn, 1908, and *Saprodinium* Lauterborn, 1908 (Corliss 1960; Kahl 1928, 1932a, b; Lauterborn 1901, 1908; Lynn 2008; Roux 1899). Kahl (1928, 1932a, b) described the majority of the known species, some of which were revisited by Jankowski (1964a), but due to incomplete or brief descriptions, many species remain unclearly defined. Most odontostomatean species have been characterized based solely on living or fixed cells, and only three of them have molecular and morphological characterization using modern methods (Fernandes et al. 2018; Jankowski 1964a, b; Kahl 1932a; Klein 1930; Méndez-Sánchez et al. 2023; Paiva et al. 2017; Schrenk and Bardele 1991; Tuffrau 1992).

The order Odontostomatida has been historically included by various authors in several ciliate classes, e.g., Spirotrichea Bütschli, 1889, Heterotrichea Stein, 1859, or Armophorea Lynn, 2004 (Corliss 1979; Jankowski 1964b, 2007). However, the classification of odontostomateans has changed with the complementation of molecular data. Based on an 18S rRNA gene sequence from a morphologically unidentified source organism, labeled in GenBank (EF014286) as *Epalxella antiquorum* (Penard, 1922) Corliss, 1960, the phylogenetic analysis of Stoeck et al. (2007), quite unexpectedly, suggested a position of odontostomatids within the class Plagiopylea Small & Lynn, 1985. More recently, 18S rRNA genes of three species, *Discomorphella pedroeneasi* Paiva et al. 2017, *Mylestoma* sp., and *Saprodinium dentatum* (Lauterborn, 1901) Lauterborn, 1908, have been partially or fully sequenced and all comprise a fully supported lineage closer to Armophorea or Litostomatea Small & Lynn, 1981 than Plagiopylea (Fernandes et al. 2018; Méndez-Sánchez et al. 2023). Accordingly, Fernandes et al. (2018) proposed the class Odontostomatea. So-called “universal eukaryotic” or “ciliate-specific” primers typically fail to amplify the divergent 18S rRNA gene of odontostomateans (Fernandes et al. 2018; Méndez-Sánchez et al. 2023). Consequently, the diversity of odontostomatean ciliates is likely underrepresented in many environmental analyses.

This study offers a comprehensive molecular and morphological characterization of 32 odontostomatean populations, representing nine known, three newly described, and three undetermined species belonging to eight genera, three of which are novel. It provides a set of odontostomatean-specific primers for 18S rRNA gene amplification. Furthermore, our analysis of available environmental transcriptomic and metagenomic data reveals a substantial diversity of potential odontostomatean sequences, highlighting a probable underestimation of odontostomatean diversity based only on limited 18S rRNA gene sequence data.

## Materials and methods

### Sampling

Hypoxic sediments from various localities (Table 1) were collected in Falcon tubes or plastic jars. Approximately 1 or 2 mL of the samples were inoculated into 9 mL of Sonneborn’s *Paramecium* medium: American Type Culture Collection (ATCC) medium #802 for freshwater samples, ATCC medium #1525 for marine samples, or a 1:1 ratio of the two media for brackish samples, and maintained at room temperature (23 °C). Tubes were tightly closed to prevent rapid oxygenation. The inoculated tubes were subcultured biweekly or monthly, as previously described (Bourland et al. 2017; Rotterová et al. 2018). Short-term cultures were successfully obtained in a few cases, but most studied populations were directly studied from the raw sample (Table 1). A total of 32 populations were studied.

**Table 1:** Origin of the studied odontostomatean populations

Population	Species found	Sampling place/habitat/year	Coordinates
3S6A^a^	Odontostomatea sp.	A small pond, Croatia. Freshwater. 2022	N.A.
BOPAT	*Saprodinium mimeticum*	A small pond in Smíchousův rybník, Karlovice, Czech Republic. Freshwater. 2020	50°33′13.1″ N, 15°10′59.8″ E
BOTAN	*Discomorphella pectinata*	A small pond in the Botanical Garden Faculty of Science, Charles University, Prague, Czech Republic. Freshwater. 2022, 2023	50°04′20″ N, 14°25′24″ E
BUL7A^a^	*Tostonella uncinata*	A small pond within the Arkutino Reserve, Primorsko, Bulgaria. Freshwater. 2021	42°19′53.0″ N 27°43′33.3″ E
BYMOKOT	*Saprodinium mimeticum*	Modřanské laguny, a small permanent pond adjacent to the Vltava River in Prague, Czech Republic. Freshwater. 2024	49°59′44.5″ N, 14°24′10.1″ E
CANSYK	Epalxellidae sp.	A small pond, Gran Canaria, Cannary Islands. Freshwater. 2024	N.A.
CEDARKOR^a^	*Tostonella uncinata*	A small pond in dunes in the vicinity of Lake Korission, “Cedar forest”, Korfu, Greece. Freshwater. 2022	39°26′09.2″ N 19°55′46.7″ E
EPOPOLO^a^	*Mircalla triangula*	A small pond, Bayanga, Central African Republic, freshwater. 2019	2°50′32″ N, 16°27′56″ E
FAROTODO^b^	*Mylestoma monodontum*	A tidal wetland sediment in Olhão, Algarve, Portugal. Brackish. 2018	37°01′49.4″ N, 7°48′32.0″ W
FRESHO^b^	*Mylestoma monodontum*	Fresh River, Falmouth, Massachusetts, USA. Brackish. 2018	41°32′30.804″ N, 70°37′19.92″ W
GLENDWOODPEL	*Pelodinium reniforme*	A sulfidic sediment near the Boise River in Boise, Idaho Glenwood runoff, USA. Freshwater. 2006	43°39′47.40″ N, 116°16′57.12″ W
GLENDWOODSA	*Saprodinium dentatum*		
GROSERDU	Odontostomatea sp. *Saprodinium dentatum*	Grosser Dutzendteich pond, in Nuremberg, Germany. Freshwater. 2021	49°25′44.7″ N, 11°07′03.7″ E
IS7^a^	*Saprodinium mimeticum*	A small pond, Croatia, freshwater. 2022	N.A.
KLAGOS^a^	*Mylestoma bipartitum*	A shore in Porto Lagos, Greece. Brackish. 2022	41°00′19.9″ N, 25°06′00.0″ E
LAPLICE	*Discomorphella pectinata*	A small pond, Łapalice, Poland. Freshwater. 2023	54°19′48.7″ N 18°08′21.3″ E
LAUFENJ	*Epalxella exigua*	Ziegelweiher pond, Laufenburg, Switzerland. Freshwater. 2021	47°33′35.5″ N, 8°04′01.6″ E
LERMA3	*Saprodinium dentatum*	Marsh, Atarasquillo, Lerma, State of Mexico, Mexico. Freshwater. 2019	19°20′37″ N, 99°30′09″ W
M1LOT	Epalxellidae sp.	A small puddle in Kaņiera Lake Reed Trail, Latvia. Freshwater. 2022	57°0′7″ N, 23°28′41″ E
MOKOT	*Discomorphella pectinata* Epalxellidae sp.	A small permanent pond adjacent to the Vltava River in Prague, Czech Republic. Freshwater. 2022–2024	49°59′24.9″ N 14°24′04.1″ E
MOKOTP2 MOKOTNOV	*Epalxella* cf.* antiquorum*		
MOKOTP1	*Mircalla polidorii*		
MOKOTPEL	*Pelodinium reniforme*		
MOKOTR	*Saprodinium dentatum*		
SLATINA^a^	*Limnomylestoma* sp. *Saprodinium dentatum*	Slatina pond, Dubeč, Czech Republic. Freshwater. 2022	50°04′13.2″ N 14°34′12.5″ E
VLKOV	*Saprodinium dentatum* *Epalxella* cf.* antiquorum*	A small permanent pond in the village of Vlkov, Czech Republic. Freshwater. 2022	49°09′04.0″ N 14°43′22.4″ E
ZLAKOR2A	*Epalxella* cf.* antiquorum*	A small pool by the river Vltava River, Zlatá Koruna, Czech Republic. Freshwater. 2021	48°51′19.0″ N 14°22′18.8″ E
ZLATOVLASKA	*Epalxella exigua*	A small pond, Pluhův Žďár, Czech Republic. Freshwater. 2021	49°14′53.4″ N 14°53′06.3″ E

*N.A.* not available

^a^Populations were cultured for a short term

^b^Populations are maintained as long-term cultures

### Morphological characterization

Living cells were studied at magnifications of 400–1000× with brightfield and differential interference contrast illumination (DIC) using an Olympus BX51 microscope equipped with an Olympus DP70 camera. Cells were fixed in 10% aqueous formalin (final concentration) and impregnated with protargol to reveal the infraciliature and nuclear apparatus, according to Pan et al. (2013) and Foissner (2014). The infraciliature of *Pelodinium reniforme* Lauterborn, 1908 was studied using silver carbonate impregnation (Foissner 2014). In vivo measurements were made from photomicrographs of uncompressed, freely swimming cells. Counts and measurements from protargol preparations were made at 1000× magnification using calibrated software (QuickPhotoCamera, Promicra, s.r.o., Prague, CZ).

For scanning electron microscopy (SEM), cells were fixed for at least 30 min in an aqueous solution of 2.5% glutaraldehyde, washed, dehydrated in an ethanol series, and dried in a Bal-Tec CPD 030 critical point dryer (Leica Microsystems, Vienna, AT). Specimens were mounted on adhesive carbon tabs on aluminum stubs and coated with Pt/Pd (80%, 20%) in a Bal-Tec SCD 050 sputter coater. Specimens were examined at an accelerating voltage of 15–20 kV in a JEOL IT200 scanning electron microscope (JEOL LV, USA). Morphological terminology is based on Kahl (1932a, b), Lynn (2008), Paiva et al. (2017), Schrenk and Bardele (1991), Tuffrau (1992), and new terminology where indicated (see Glossary).

### DNA extraction, amplification, and sequencing

One to 30 cells from each of 27 odontostomatean populations were hand-picked with glass micropipettes, washed five times with distilled water or filtered culture medium, and added into 30 μL of DNA/RNA shield (Zymo Research, Irvine, CA, USA). Genomic DNA was isolated using the MasterPure™ Complete DNA and purification Kit following the manufacturer’s instructions.

For amplification of the partial 18S rRNA gene sequence (about 1500 bp), oligonucleotide primers (Supplementary Table S1) were designed in silico for different odontostomatean genera, using eight sequences of *Mylestoma* sp., *Saprodinium dentatum*, and *Discomorphella pedroeneasi* already available in GenBank. Thermocycler conditions were as follows: 94 °C for 5 min, followed by 35 cycles of 30 s at 94 °C, 30 s at 45 or 52 °C (annealing, Supplementary Table S1), and 2 min at 72 °C, and a final extension cycle at 72 °C for 5 min. PCR products were verified by 1% agarose gel electrophoresis and purified using EXOSAP (Applied Biosystems) or the Gel/PCR DNA Fragments Extraction Kit (Qiagen, Hilden, Germany). In some cases, to increase the amount of PCR product, the initial products were reamplified using the same primers as for the first reaction.

The purified PCR products from *Saprodinium mimeticum* (Penard, 1922) Kahl, 1932 (BOPAT population), *Mylestoma bipartitum* (Gourret & Roeser, 1886) Kahl, 1928 (KLAGOS population), Odontostomatean sp. 3 (3S6A population), and *Discomorphella pectinata* (Levander, 1894) Corliss, 1960 (MOKOT population) were cloned using the pGEM-T Easy Vector System I kit and JM109 competent cells of *Escherichia coli* (Promega). The plasmids were amplified using the primers T7 (5′-TAATACGACTCACTATAGGG-3′) and SP6 (5′-ATTTAGGTGACACTATAG-3′). Cloning procedures were done following the manufacturer’s instructions. Four clones were sequenced from *S*. *mimeticum* BOTAN and three from the other two populations.

The purified PCR products were sequenced at the Laboratory of DNA Sequencing (Charles University, Prague, Czech Republic) on an ABI PRISM 3100 sequencer (Applied Biosystems) or at Macrogen (Amsterdam, The Netherlands) on a 3730xl DNA Analyzer. Sequences were assembled in Geneious Prime® 2024.0.7, using de novo assembly options or mapped to a reference sequence (*Saprodinium dentatum* OP034694). All the partial sequences originating from clones of each species were assembled together.

### Phylogenetic analyses

The newly obtained sequences, excluding identical sequences from each population, and 104 sequences obtained from GenBank representing all ciliate classes, were aligned using the G-INS-I algorithm in MAFFT v7.520 (Katoh 2002). The alignment (1944 positions) was manually edited using AliView (Larsson 2014) to trim primer sequences and remove columns with singletons. The final dataset included 1805 positions. Non-corrected genetic distances were calculated in MEGA v12 (pairwise deletion to ambiguous positions) (Kumar et al. 2024). Phylogenetic trees were constructed by maximum-likelihood (ML) and Bayesian inference (BI) methods under the GTR + I + Γ as the best-fit model by ModelGenerator v0.85 (Keane et al. 2006) according to the Akaike Information Criterion. ML analysis was performed in RAxML HPC v8.2.4 (Stamatakis 2014). Node support was assessed by 1000 bootstrap pseudoreplicates. Bayesian analysis was performed using MrBayes v3.2.7 (Ronquist et al. 2012). Four Markov chain Monte Carlo simulations were run for 10 million generations with a sampling frequency of 1000 generations. The first 25% of sampled trees were removed as burn-in; convergence was assessed using Tracer v1.7.2 (Rambaut et al. 2018); the average standard deviation of split frequencies was 0.003 after 10 million generations. Both ML and BI analyses were performed in CIPRES Science Gateway (Miller et al. 2010).

A possible close relationship between *Mylestoma* and *Limnomylestoma* gen. nov. was tested by the approximately unbiased (AU) test in consel 0.1i (Shimodaira and Hasegawa 2001). Two alternative hypothesis were tested: (1) *Mylestoma* spp. and *Lymnomylestoma shuriken* sp. nov. form a clade, and (2) *Mylestoma* spp., *Lymnomylestoma shuriken* sp. nov., and the environmental sequence AY821980 form a clade. In the tests, the overall best tree topology was compared with the best trees found under respective constraint, which were computed by maximum likelihood in RAxML HPC v.8.2.4 as described above.

### Environmental analyses

Unique sequences of odontostomateans (*N* = 16) were queried in the PebbleScout web server (Shiryev and Agarwala 2024), using metagenomic and whole genome sequencing (WGS) databases with default parameters. A threshold of ≥ 90 PBScore was chosen, resulting in 42 environmental samples after dereplication between queries (Supplementary Tables S2, S3). For the metatranscriptomic hits (*N* = 41), assemblies were created using Trinity v2.15.1 and rnaSPAdes v3.15.4. For the single metagenomic hit, a publicly available assembly was downloaded. From all the assemblies, the 18S rRNA gene sequence was extracted by barrnap v0.9 (github.com/tseemann/barrnap), and the extracted sequences were added to a subset of a pan-eukaryotic dataset, which contained 88 sequences representing the broad eukaryotic diversity (not shown). The dataset was aligned using MAFFT v7.520 and trimmed (BMGE v1.12). The phylogenetic tree was constructed with IQ-TREE v2.3.4 (Minh et al. 2020) using a model selected by ModelFinder (Kalyaanamoorthy et al. 2017). The statistical support was tested with 1000 ultrafast bootstraps (Hoang et al. 2018). The datasets were then progressively refined by deleting distant sequences and repeating the phylogenetic tree construction pipeline until sequences within the Odontostomatea clade were readily identifiable. Finally, the filtered sequences were checked for chimeras manually and automatically using VSEARCH v2.14.1 (Rognes et al. 2016). A final dataset comprising 2001 positions was constructed by aligning the 263 odontostomatean environmental sequences, the 36 odontostomatean sequences, and 104 ciliate sequences from GenBank (see above) using the G-INS-i algorithm in MAFFT v7.520. The alignment was manually trimmed to remove primers and singletons, resulting in a final dataset with 1890 positions. The minimum length of sequences was 419 bp. The phylogenetic tree was constructed in RAxML v8.2.12, using the model GTR + I + Γ and 100 standard bootstraps.

## Results

### Phylogeny of Odontostomatea based on the 18S rRNA gene sequences

**Fig. 1:**
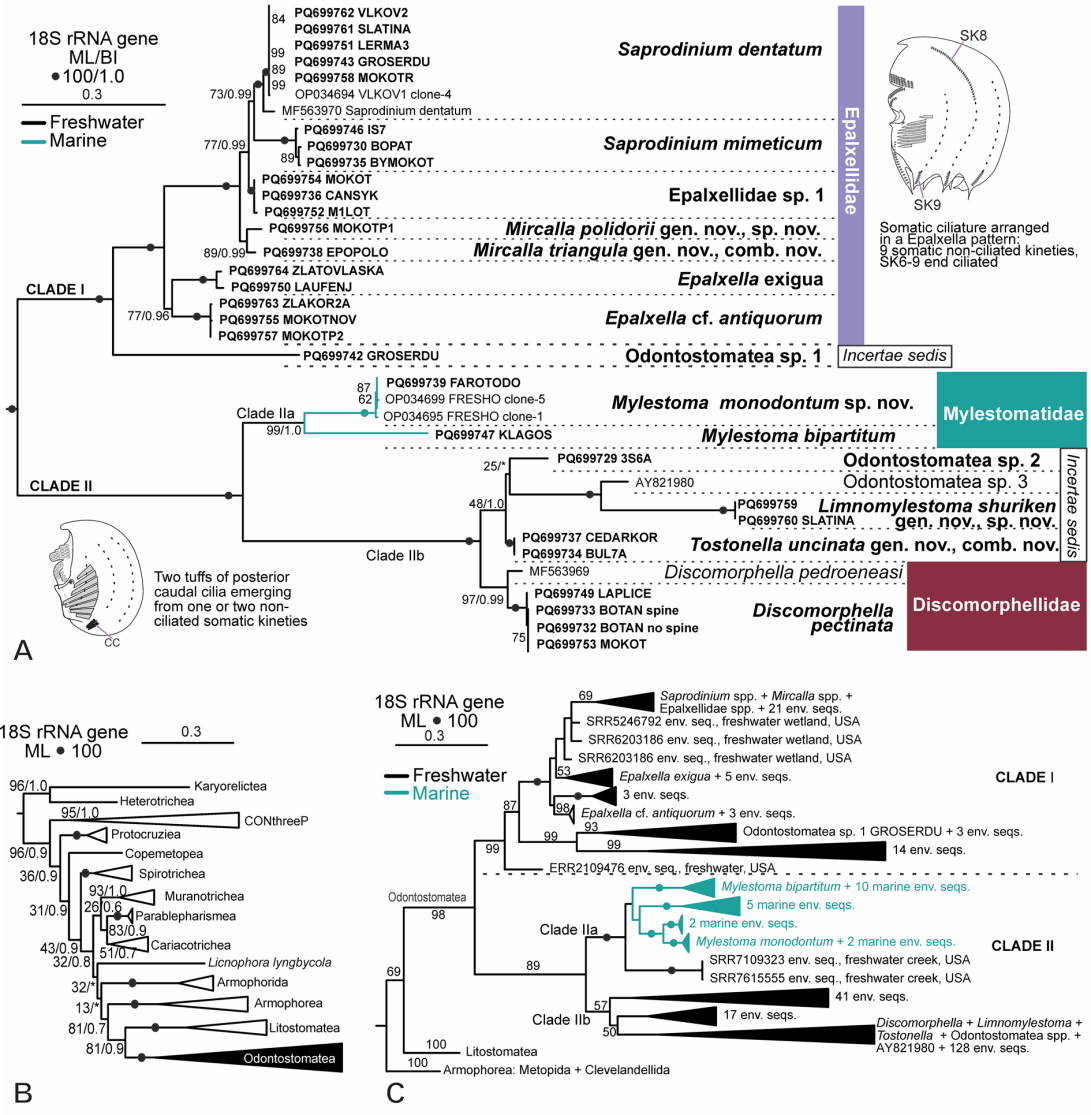
Phylogeny of Odontostomatea based on 18S rRNA gene. **A** ML phylogenetic tree showing the newly sequenced populations and species (in bold) of odontostomateans. **B** ML tree showing the position of Odontostomatea within Ciliophora after adding the new sequences. **C** ML phylogenetic tree including the environmental odontostomatean sequences from metatranscriptomes and metagenomes, plus the newly obtained sequences. For a full tree, refer to Supplementary Fig. S1. Branch values represent bootstrap support of ML and Bayesian posterior probabilities of BI, and values below 50 and 0.80, respectively, are not shown or are represented by an (*). Scale bars: 30 changes per 100 nucleotides

Thirty-six 18S rRNA partial sequences were obtained from 27 populations of 14 odontostomatean species using different pairs of newly designed primers (Supplementary Table S1). Odontostomateans form a fully supported clade, which branches sister to Litostomatea with low-to-moderate support (Fig. 1B). Odontostomatea comprises two fully supported clades, here referred to as the Clade I and Clade II (Fig. 1A). Clade I includes two main branches: one of them solely contains an undescribed species (GROSERDU population), whereas the second branch represents the fully supported family Epalxellidae. Epalxellidae comprises the well-known genera *Saprodinium*, *Epalxella*, the new genus *Mircalla* gen. nov., and three populations assigned as Epalxellidae sp. 1. Clade II contains *Mylestoma* (Mylestomatidae), *Discomorphella* (Discomorphellidae), and two new genera, *Tostonella* gen. nov. and *Limnomylestoma* gen. nov., as well as one undescribed odontostomatean (3S6A population) and one uncultured freshwater eukaryote (sequence AY821980) that cannot be assigned to any known odontostomatean family (Fig. 1A).

Uncorrected genetic distances are shown in Supplementary Table S4. The two constrained topologies, in which *Limnomylestoma* is closely related to *Mylestoma* or forms a clade with *Mylestoma* and the sequence AY821980, were both rejected by the AU test (Supplementary Table S5).

### Environmental survey of odontostomateans

A total of 263 unique partial 18S rRNA sequences retrieved from available environmental metagenomic and metatranscriptomic data (Supplementary Tables S2, S3) were identified as potential odontostomatean sequences. The topology of the phylogenetic tree of environmental sequence, which includes our isolated populations, is consistent with the 18S rRNA gene sequence tree of Odontostomatea, showing the two main clades (Fig. 1C; Supplementary Fig. S1). Fifty-three sequences are distributed within Clade I, and the remaining 210 in Clade II.

Within Clade I, Odontostomatean sp. 1 (GROSERDU population) forms a fully supported clade with three environmental sequences and is sister to another well-supported clade containing 14 sequences. These two groups form a well-supported clade that is sister to the highly supported Epalxellidae clade. Epalxellidae is internally unresolved.

Clade II is divided into two major lineages: the well-supported Clade IIa, and the unsupported Clade IIb. Clade IIa contains two groups: a highly supported clade with two freshwater environmental sequences and a weakly supported clade consisting of *Mylestoma bipartitum* and *Mylestoma monodontum* sp. nov., and environmental sequences from brackish and marine environments, distributed across four highly supported subclades.

Clade IIb contains three poorly supported clades. Two of them consist entirely of environmental sequences (41 and 17 sequences, respectively), while the third clade includes sequences from *Discomorphella* spp., *Limnomylestoma shuriken* sp. nov., *Tostonella uncinata* (Penard, 1922) comb. nov., Odontostomatea spp., and 128 environmental sequences, which are not fully resolved internally.

### General morphology of odontostomateans

**Fig. 2:**
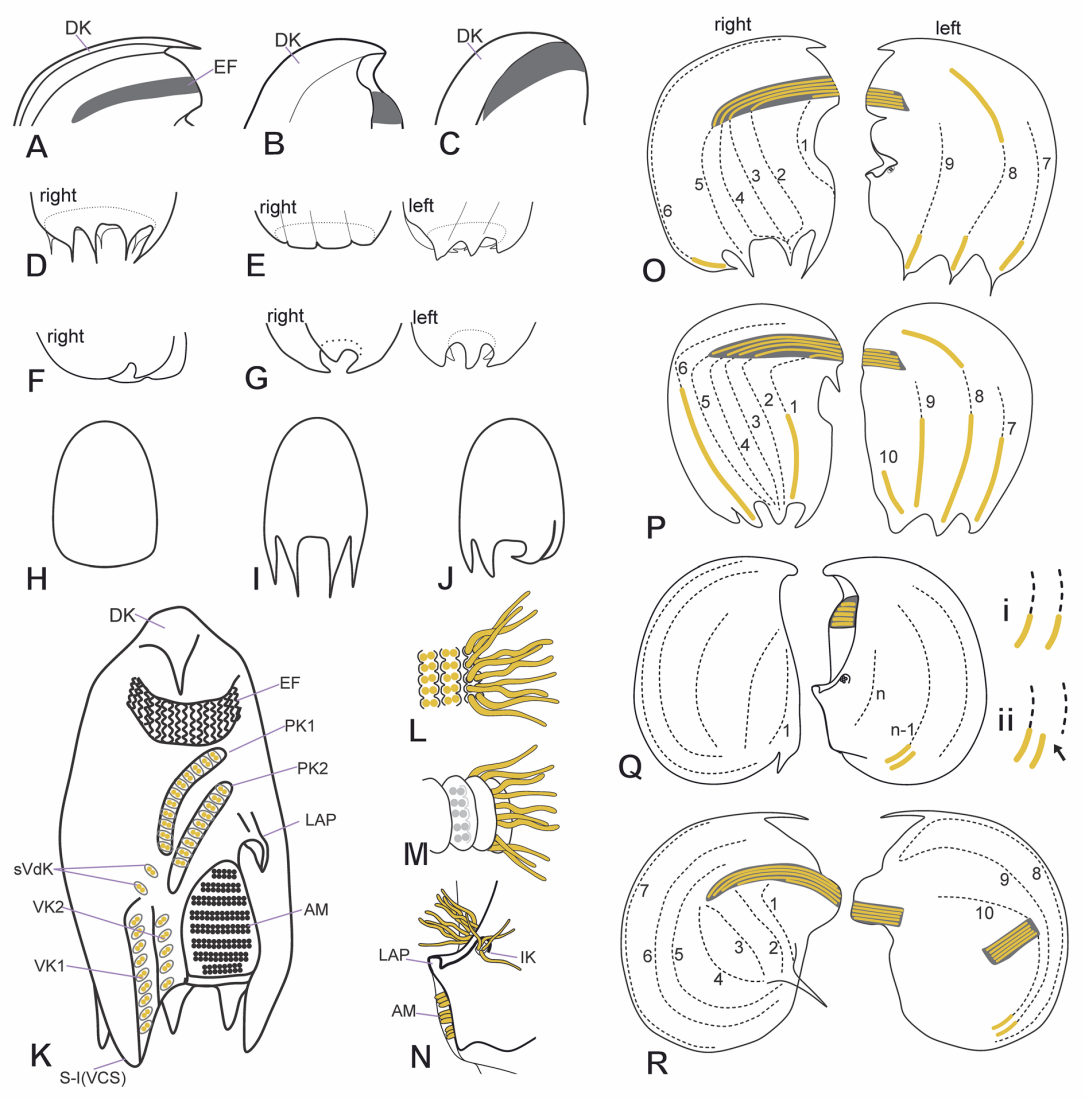
General morphology of Odontostomatea in vivo (**A**–**J**) and after impregnation (**K**–**R**). Shape of the anterior region of the cell on the lateral view, ending as a frontal spine (**A**), as a hood-like structure (**B**) or blunt (**C**). Shape of the posterior end, on lateral views, with prominent spines and broad posterior marginal concavity (**D**), wavy and with inconspicuous spines and broad posterior marginal concavity (**E**), smooth with reduced spines (**F**), or small heart-shaped concavity (**G**). Posterior end in dorsal view ending smooth (**H**), with spines on both sides (**I**), or with concavity with inwardly folding spines (**J**). **K** General ventral view of the ciliature. **L**, **M** Organization of the pectinelles without (**L**) or with auricles (**M**). **N** Left lateral view of the buccal area showing the disposition of the lapel-like structure and the inverse kinety. Different ciliature patterns: *Epalxella* pattern (**O**), *Pelodinium* pattern (**P**), *Mylestoma* pattern (**Q**) with caudal cirrus arranged as an amphikinetal pattern (**Qi**), and a semikinetal pattern (**Qii**), and *Discomorphella* pattern (**R**). *AM* adoral membranelles, *DK* dorsal keel, *EF* epistomial fringe, *IK* inverse kinety, *LAP* lapel-like structure, *PK* preoral kinety, *S-1* spine 1, *sVdK* supplemental ventral dikinetid, *VCS* ventrocaudal spine, *VK* ventral kinety

Odontostomateans’ morphology is exceptionally complex and can be described with a rather specialized terminology (Paiva et al. 2017; Schrenk 1989; Schrenk and Bardele 1991; Tuffrau 1992) (see Glossary). The cell size varies, ranging from 20 to ~ 90 µm. The cells are usually almost circular in outline, but exceptions are found (e.g., triangular, quadrangular), strongly laterally compressed, and show right- and left-side asymmetry. The armor-like cortex is rigid, inflexible, and may bear *cortical slits* (“Fensterbildungen”) and *cortical pits*. The anteroventral portion ends as a frontal spine (Fig. 2A), a hood-like structure (Fig. 2B), or a blunt shape (Fig. 2C), continuing posteriorly as a *dorsal keel* (Fig. 2A–C), which might fold rightwards either completely forming a carapace-like (Fig. 2B, C) or just slightly forming a shallow trough that runs posteriorly (Fig. 2A). The posterior margin is either rounded with or without spines (Fig. 2F, H), or it is concave and bordered by prominent to inconspicuous spines, which are sometimes hidden in the posterior concavity (Fig. 2G, I, J).

The nuclear apparatus consists of one-to-four spherical macronuclear nodules and invariably a single spherical micronucleus. The cytoplasm is colorless and contains globules of various sizes. Food vacuoles contain prokaryotes and sometimes algae. A single contractile vacuole is usually located in the center of the lateral right side or posteriorly. Mitochondrion-related organelles are distributed in the cytoplasm. Endosymbiotic prokaryotes, including UV-autofluorescent methanogenic archaea, lie freely in the cytoplasm. The swimming pace varies from slow to fast, and the cell usually rotates around the long axis when swimming or crawls along debris on its right side.

The infraciliature is sparse and reduced to 12 or fewer kineties consisting of dikinetids in *ciliary sockets*. The dikinetids may have one or both basal bodies ciliated or may be barren. The kineties usually consist of three segments: an anterior ciliated segment, a non-ciliated middle segment, and a posterior ciliated segment often located on a spine. The non-ciliated segments are composed of *subsurface basal bodies* (barren dikinetids). There are usually two kineties on the ventral margin; the anterior and posterior ciliated segments of these kineties are called preoral kineties and ventral kineties, respectively. Between both segments, a row of a few supplementary ciliated ventral dikinetids may be present. The remaining kineties, which are on the lateral sides, are called somatic kineties (*SK*). The anterior ciliated segments of the first five kineties (*SK1*–5) constitute the epistomial fringe. The posterior ciliated segments of the somatic kineties usually end on the posterior spines and are called spine segments. On the left side, two caudal cirri, bearing long cilia, may emerge at the end of one or two somatic kineties. Additionally, an inverse kinety and two endoral kineties may be present. Usually, there are nine or ten adoral membranelles. The paroral membrane is absent. Four infraciliary patterns are recognized (see below).

The *ventral ridge* lies above the equator and bears the *epistomial fringe*. The *ventral ridge* may protrude as a *frontal awning*. Just posterior to it, two diagonal furrows usually contain the preoral kineties. The buccal cavity is located on the ventral margin in the posterior half of the cell, nearer to the left surface and just posterior to the preoral kineties. The buccal cavity is flanked by either a *ventrocaudal spine* (spine 1) or a *ventral flap* on its right side, a rigid plate on its left side, and a buccal lip posteriorly. The ventrocaudal spine or ventral flap bears the ventral kineties and supplementary ventral dikinetids. The anterior portion of the left buccal plate usually folds outward, forming a *lapel-like structure* that embraces the *inverse kinety*, which generally consists of two or three dikinetids (Fig. 2N). Based on transmission electron microscopy (TEM), the *inverse kinety* shows an inverse orientation with respect to the longitudinal cell axis. Inside the buccal cavity, up to ten truncated adoral membranelles with inverted orientation and separated by septa are contained in at least two compartments, usually most membranelles in the outer compartment and one in the inner compartment.

The *epistomial fringe* is ventrally supported by the *ventral ridge* and consists of five ciliary rows of variable length that run horizontally above the equator. The epistomial ciliary rows form longitudinal *pectinelles* separated by notched interkinetal ridges (Fig. 2L) or *auricules* (Fig. 2M). The *epistomial fringe* commences on the left side of the cell, embracing the *ventral ridge*, and ending on the right side in a staggered manner. It may be continuous or comprise two fragments: a dextro-frontal and a left fragment, separated by an *epistomial fringe spacer*. *Papillary dikinetids* may be present at the end of the *epistomial fringe*.

### Somatic infraciliary patterns

The *epistomial fringe* and the somatic kineties are arranged in at least four patterns: the *Epalxella* pattern (Fig. 2O), *Pelodinium* pattern (Fig. 2P), *Mylestoma* pattern (Fig. 2Q), and *Discomorphella* pattern (Fig. 2R).

The ***Epalxella* pattern** (Fig. 2O) invariably consists of nine somatic kineties (*SK1–9*). The *SK1–6* are located on the right, and *SK7–9 *are on the left side. Five kineties (*SK1–5*) consist of an anterior ciliated segment (epistomial ciliary rows) and a posterior non-ciliated segment each; *SK1* ends on the posterior ventral margin, and *SK2–5* end posteriorly. Three kineties (*SK6, 7, and 9*) consist of an anterior non-ciliated segment that ends as a posterior ciliated segment. One kinety (*SK8*) consists of an anterior ciliated segment, followed by a non-ciliated middle segment, and ending as a posterior ciliated segment. *SK6* runs along the edge of the *dorsal keel*, whereas *SK7* runs almost on the dorsal margin. The anterior segment of *SK8* is located in the anterior third of the cell. The non-ciliated segments of *SK7*–9 begin in the middle third of the cell, extending as parallel rows posteriorly. The four posterior ciliated segments of *SK6*–9 are short, restricted to the spines, and separated by a short gap from the non-ciliated segments.

The ***Pelodinium* pattern** (Fig. 2P) consists of ten somatic kineties (*SK1–10*). The *SK1–6* are located on the right, and *SK7–10* are on the left side. Four kineties (*SK2–5*) consist of an anterior ciliated segment (epistomial ciliary rows) and a posterior non-ciliated segment each, and ending posteriorly. Three kineties (*SK6, 7*, and *9*) consist of an anterior non-ciliated segment that ends as a posterior ciliated segment. Two kineties (*SK1* and *SK8*) consist of an anterior ciliated segment, followed by a non-ciliated middle segment, and ending as a posterior ciliated segment. One kinety (*SK10*) consists solely of a ciliated segment and is located in the ventral margin at the posterior third of the cell. *SK6* runs along the edge of the *dorsal keel*, whereas *SK7* runs almost on the dorsal margin. The anterior ciliated segment of *SK1* is an epistomial ciliary row. The anterior segment of *SK8* is located in the anterior third of the cell. The non-ciliated segments of *SK7–9* begin in the middle third of the cell, extend shortly as parallel rows, and are restricted to the middle third of the cell. The five posterior ciliated segments of *SK1, 6–9*, are long, commencing at or slightly above the cell equator, running posteriorly, ending on spines, and separated by a short gap from the non-ciliated segments.

The ***Mylestoma* pattern** (Fig. 2Q) consists of nine or fewer somatic kineties (*SK1–9*) distributed on the lateral sides and constantly two caudal cirri on the posterior left side. All of the kineties are non-ciliated. A short gap posteriorly separates one or two of the kineties on the left side from the caudal cirri. Two types of arrangement of the caudal cirri are recognized: (a) amphikinetal pattern, where each caudal kinety is connected to a somatic kinety (Fig. 2Qi), and (b) semikinetal pattern, where one caudal kinety is connected to a somatic kinety, whereas the second one is disconnected or misplaced from a somatic kinety (Fig. 2Qii). Posterior marginal spines are reduced in *Mylestoma* species (Fig. 2Q).

The ***Discomorphella* pattern** (Fig. 2R) comprises around nine *non-ciliated kineties*. The *SK1–4* are posterior to the dextro-frontal fringe on the surface of the vaulted right side. The *SK5–7* are arched, starting anteriorly following the margin of the dorsal keel and ending posteriorly; the *SK6–7* end below the posterior spine, whereas the *SK7* continues on the posterior margin and possibly joins the end of the ventral kinety 2. On the left side, the *SK8* is arched, running on the dorsal keel margin, anteriorly folds backward, and continues posteriorly, ending in one of the caudal cirri. The *SK9* runs obliquely from the middle third of the cell, connecting to the most dorsal pectinelle of the left epistomial fragment, and continues posteriorly, possibly ending in the second caudal cirri. Scattered subsurface basal bodies are between the left and the dextro-frontal epistomial fragments (Klein 1930; Tuffrau 1992).

### Occurrence and ecology

Odontostomateans are bacterivorous, although small flagellates can also be ingested. They are distributed worldwide in oxygen-depleted freshwater, brackish water, or marine sediments. They are obligate anaerobes living in symbiotic syntrophic associations with prokaryotes, likely methanogenic archaea in freshwater (Foissner et al. 1992).

### Descriptions of new and known odontostomatean species

***Saprodinium mimeticum*** **(Penard, 1922) Kahl, 1932**

**Fig. 3:**
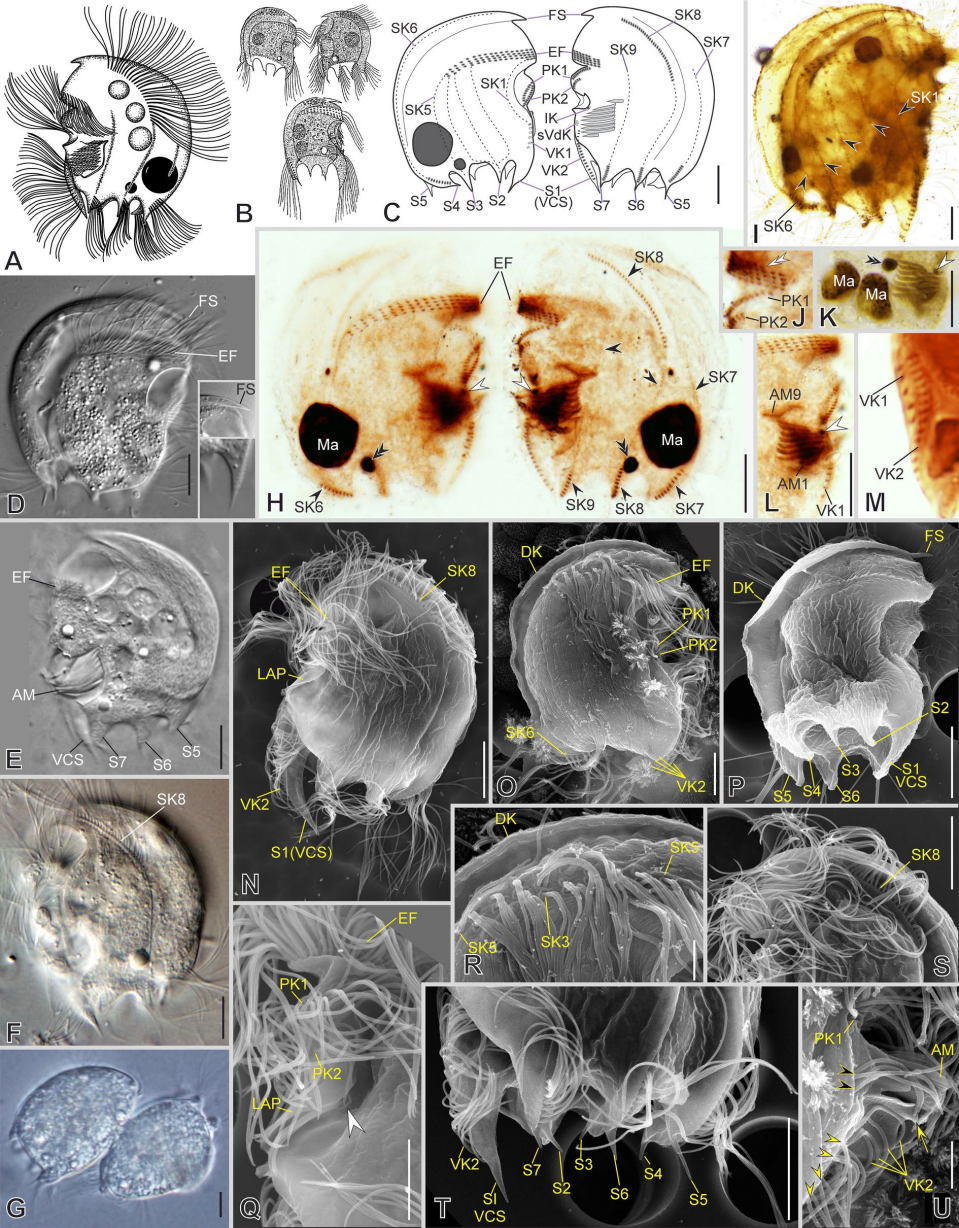
Morphology of *Saprodinium mimeticum* in vivo (**A**, **B, D**–**G**), after impregnation (**C**, **H**–**M**), and in SEM (**N**–**U**). **A**–**F**, **H**, **I**, **N**–**P** General cell shape and infraciliature showing somatic kineties (*SK*, black arrowheads) in an *Epalxella* pattern. Frontal spine (*FS*) and ventrocaudal spine (*S1-VCS*) (inset in **D**). **B** After Kahl (1932). **G** Two cells during conjugation. **J, Q** Detail of epistomial fringe (*EF*), preoral kineties (*PK*), lapel-like structure (*LAP*) and inverse kinety (*IK*, white arrowheads). Displacement of fifth epistomial row (white double arrowhead). **K**, **L** Detail of macronuclei (*Ma*), micronucleus (black double arrowheads), adoral membranelles (*AM*), and *IK* (white arrowheads). **M**, **U** Detail of ventral kineties (*VK*, yellow arrowheads), supplementary ventral dikinetids (*sVdK*, black arrowheads), buccal lip (yellow arrow), and *AM*. **R** Detail of dorsal keel (*DK*) and shallow trough, and *EP’s* end. **S** Anterior ciliated fragment of *SK8*. **T** Distribution of posterior spines (*S*, left lateral view). Scale bars: 10 µm (**A**–**P**), 5 µm (**Q**–**U**)

***Redescription based on IS7, BYMOKOT, and BOPAT populations ***(Fig. 3; Supplementary Table S6, Video S1): Cells 49–55 × 32–43 μm in vivo and 37 × 30 μm on average in protargol-impregnated specimens. The shape is almost quadrangular in outline (Fig. 3A–E, N). Anteriorly, a short frontal spine (4.5 μm on average), followed by a dorsal keel slightly folded rightwards, forming a shallow trough ending subposteriorly (Fig. 3A–D, O, P, R). Seven slender, sharp, conspicuous spines surround a concavity at the posterior cell margin, three on the left and four on the right, including the conspicuous and prominent, sharp, curved, and large ventrocaudal spine (S1) (Fig. 3C, E, N, P, T). The lapel-like structure enfolds the two dikinetids of the inverse kinety (Fig. 3N, Q). One-to-four (three on average) macronuclear nodules and a single micronucleus (Fig. 3C, H, I, K).

On average, the continual epistomial fringe comprises 24 pectinelles, 8 on the left and 16 on the right (Fig. 3C–E, H–J, L, O, R). The two preoral kineties (PK1-2) have an average of 20 and 14 dikinetids, respectively (Fig. 3C, H, J, L, O, Q). The two ventral kineties (VK1–2) have an average of 6 and 16 dikinetids, respectively, and are located on the ventrocaudal spine; the VK1 is more superficial than the VK2 (Fig. 3C, M, N, O, U). A pair of supplementary ventral dikinetids runs anteriorly to the VK1 (Fig. 3U). The oral cavity invariably has nine membranelles, eight within the outer compartment and the ninth in the inner (Fig. 3C, H, K, L, U).

The somatic kineties are arranged in the *Epalxella* pattern (Fig. 3C, H, I, N, O). The SK1 ends on the posterior ventral margin. The posterior ends of SK2–4 join at the base of spine 2. The SK5 ends in the spine 3. The SK6 ends in the spine 4 with 9 ciliated dikinetids (Fig. 3C, H, O). The SK8 begins with 13 ciliated dikinetids (Fig. 3C, F, H, N, S). The SK7, 8, and 9 end in the spines 5, 6, and 7, respectively, with an average of 8, 7, and 6 dikinetids, respectively (Fig. 3C, H, T).

***Notes:*** The morphometric values given in the above description are based on the IS7 population. Refer to Supplementary Table S6 for the morphometric values of the other two populations. The BOPAT and IS7 populations are highly similar to each other; the only notable difference between them is the number of dikinetids in the anterior ciliated segment of SK8 (21 vs. 13, on average, respectively) (Supplementary Table S6). The data from BYMOKOT population were insufficient for a morphometric description.

***Notes on 18S rRNA gene sequences:*** The genetic distance (uncorrected p distance) between the three studied populations (IS7, BYMOKOT, and BOPAT) ranges from 0 to 0.004 (Supplementary Table S4).

### ***Saprodinium dentatum* (Lauternorn, 1901) Lauterborn, 1908**

**Fig. 4:**
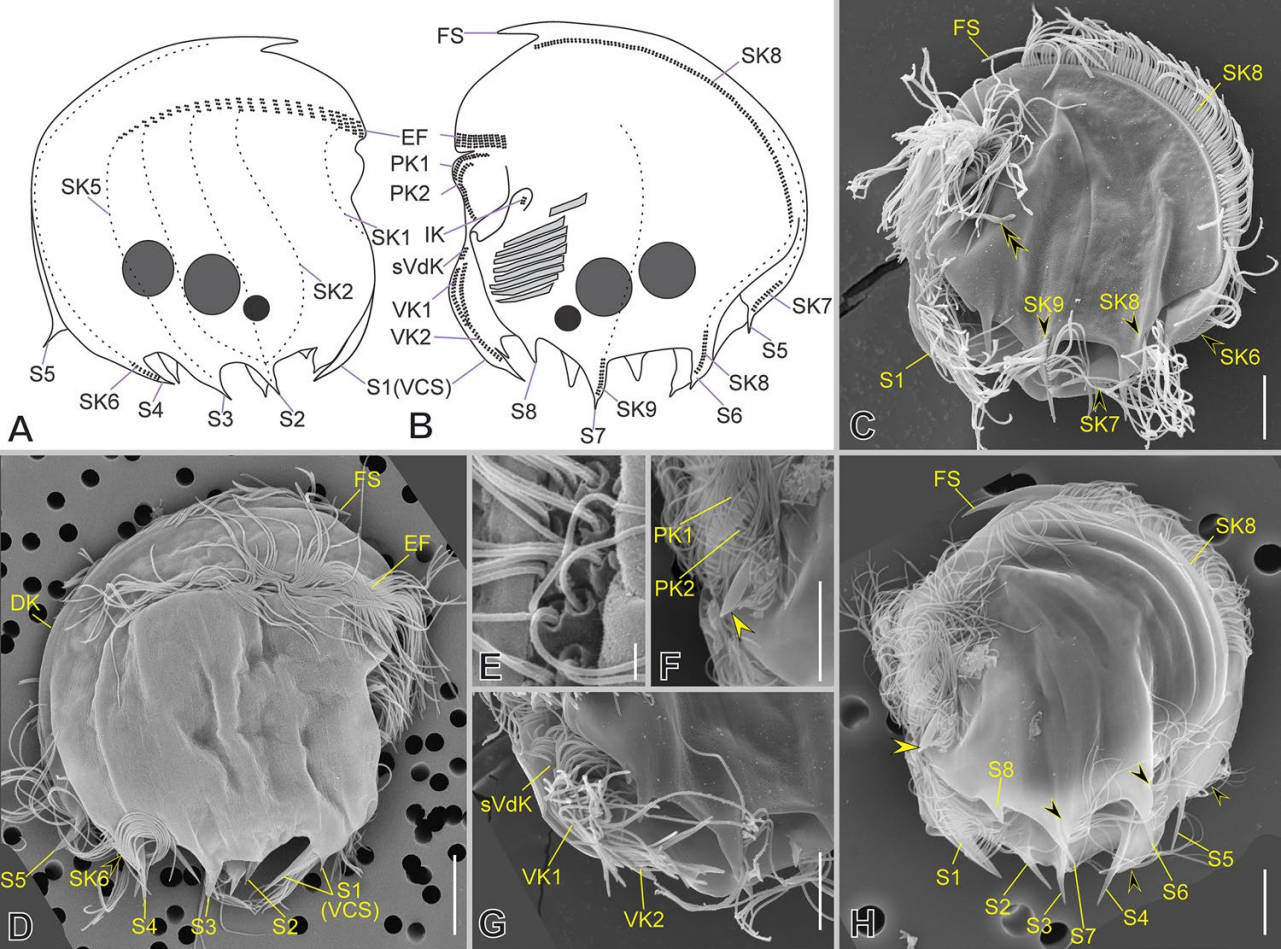
Morphology of *Saprodinium dentatum* after impregnation (**A**–**B**) and in SEM (**C**–**H**). **A**–**D**, **H** General cell shape, dorsal keel (*DK*), distribution of posterior spines (*S*) and their spine segments of kineties (black arrowheads), and infraciliature showing somatic kineties (*SK*) in an *Epalxella* pattern. Epistomial fringe (*EF*); frontal spine (*FS*); inverse kinety (*IK*) (black double arrowhead). **E** Epistomial ciliary sockets bearing dikinetids. **F** Left lateral view of the mid-ventral margin showing lapel-like structure (yellow arrowhead) and preoral kineties (*PK*). **G** Ventro-lateral view showing the ventral kineties (*VK*) and supplementary ventral dikinetids (*sVdK*), and posterior cavity surrounded by spines (*S*). Scale bars: 10 µm (**C**, **D**, **F**–**H**), 2 µm (**E**)

**Fig. 5:**
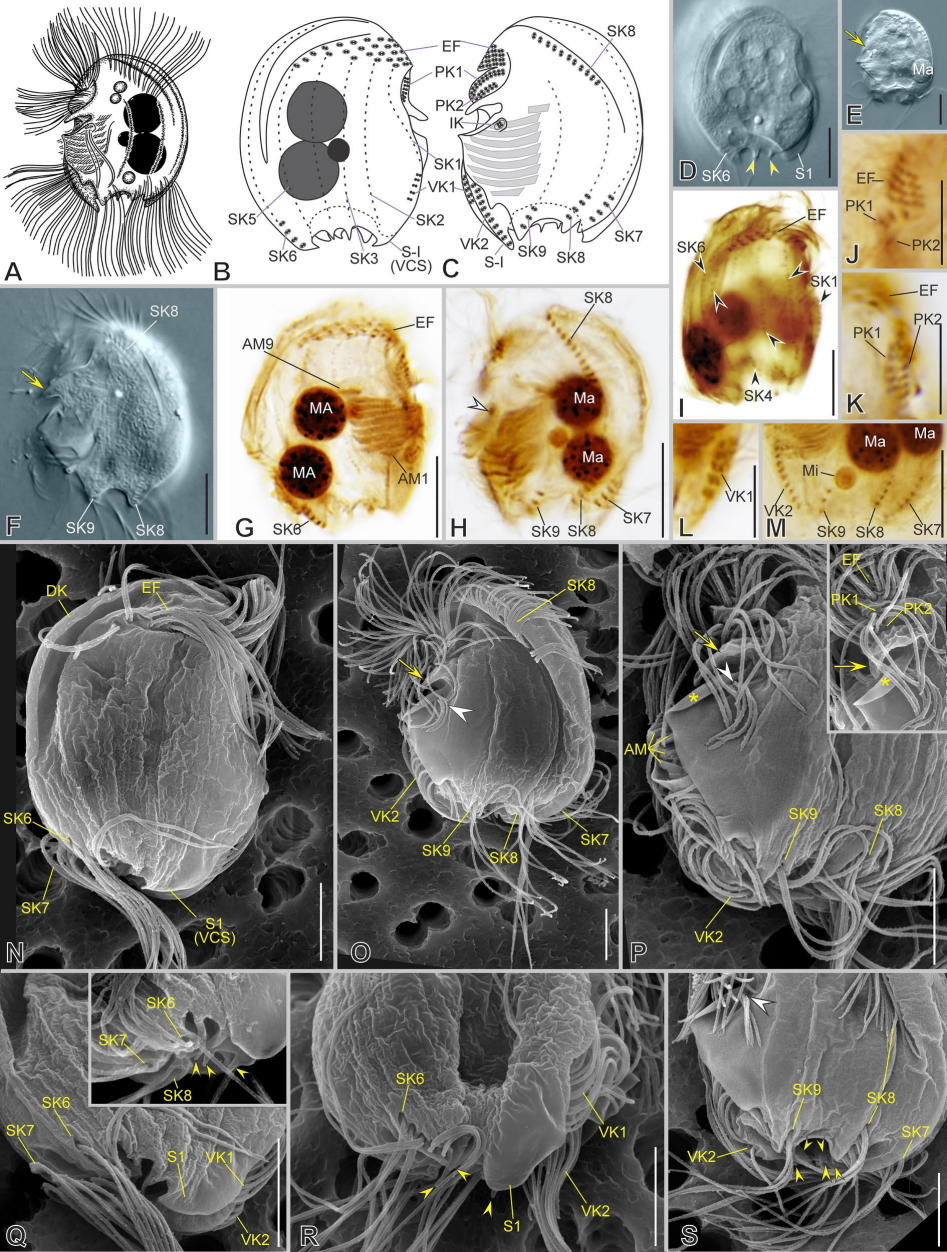
Morphology of *Mircalla polidorii* gen. nov., sp. nov. in vivo (**A**, **D**–**F**), after impregnation (**B**, **C**, **G**–**M**), and in SEM (**N**–**S**). **A**–**I**, **N**, **O** General cell shape, dorsal keel (*DK*) and shallow trough, ventral spine (yellow arrows), and infraciliature showing somatic kineties (*SK*, black arrowheads) in an *Epalxella* pattern. **J**, **K**, **P** Detail of epistomial fringe (*EF*), preoral kineties (*PK*), lapel-like structure (asterisks), inverse kinety (*IK*, white arrowhead), and adoral membranelles (*AM*). **L**, **M**, **Q**–**S** Detail of macronuclei (*Ma*) and micronucleus (*Mi*); distribution of ventral knieties (*VK*), ciliated spine segments of *SKs*, and posterior spines (*S*, yellow arrowheads). Scale bars: 10 µm

***Additional notes to the description of VLKOV population ***(Fig. 4; Supplementary Table S7, Video S2): The shape is discoidal with a frontal spine and eight-to-nine posterior sharp spines. The dorsal keel is conspicuous. The lapel-like structure enfolds the three dikinetids of the inverse kinety (Fig. 4B, C, F, H). The two ventral kineties (VK1–2) lie in the ventrocaudal spine, and a row of four supplementary ventral dikinetids runs anteriorly to the VK1 (Fig. 4B, C, G). The somatic kineties are arranged in the *Epalxella* pattern (Fig. 4A, B). The SK1 ends in the posterior ventral edge. The posterior ends of SK2–4 join at the base of the spine 2. The SK5 ends in spine 3 (Fig. 4A). The SK6 ends in spine 4 with 13 ciliated dikinetids (Fig. 4A). The SK7, 8, 9 end as a spine segment with 12, 12, and 10 ciliated dikinetids ending in the spine 5, 6, and 7, respectively (Fig. 5B). The SK8 with 94 ciliated dikinetids on average in its anterior segment.

***Notes on 18S rRNA gene sequences:*** The genetic distance (uncorrected p distance) between the five studied populations (VLKOV, MOKOT, SLATINA, GROSERDU, and LERMA3) and the published population from Brazil and the previous VLKOV population (MF563970, OP034694) ranges from 0 to 0.01 (Supplementary Table S4).

### ***Mircalla polidorii* gen. nov., sp. nov.**

***Description based on MOKOTP1 population*** (Fig. 5; Supplementary Table S8, Video S3): Cells 30–36 × 23–28 μm in vivo and 26 × 19 μm on average in protargol-impregnated specimens. The shape is almost ellipsoidal in outline (Fig. 5A, D, E, N, O). The frontal spine is absent; the dorsal keel folds slightly rightwards, forming a shallow trough ending subequatorially (Fig. 5D, N). About seven short, inconspicuous spines surround a concavity at the posterior cell margin; three of them are visible on the left side, whereas the remaining are on the right side and inwardly curved, except for the ventrocaudal spine, making the posterior right margin wavy (Fig. 5D, F, N, Q–S). The lapel-like structure enfolds the two dikinetids of the inverse kinety (Fig. 5C, O, P, S). A conspicuous fang-like spine is on the ventral margin toward the left side, between the anterior end of the preoral kineties and the lapel-like structure (Fig. 5A, E, F, O, P). One or two (usually two) macronuclear nodules and a single micronucleus (Fig. 5B, G, H, M).

On average, the continual epistomial fringe comprises 12 pectinelles, three on the left side and nine on the right (Fig. 5B, C, G, I–K, N, O). The two preoral kineties (PK1–2) have an average of nine and five dikinetids, respectively (Fig. 5B, C, I, K, P). The two ventral kineties (VK1–2) have an average of four and ten dikinetids, respectively, and lie in the ventrocaudal spine; the VK1 is more superficial than the VK2 (Fig. 5B, C, L, M, O, P–S). The oral cavity invariably has nine membranelles, eight within the outer compartment and the ninth in the inner (Fig. 5C, G, P).

The somatic kineties are arranged in an *Epalxella* pattern (Fig. 5B, C, G, H, I). The SK1 ends ventrally. The SK2–5 ends posteriorly, likely in the reduced spines. The SK6 ends in a reduced spine with five ciliated dikinetids (Fig. 5G, N, Q, R). The SK8 begins with an anterior ciliated segment having 13 ciliated dikinetids (Fig. 5C, H, O). The SK7, 8, and 9 end in reduced spines with an average of 5, 4, and 2 dikinetids, respectively (Fig. 5M, O, P, S).

### ***Mircalla triangula* (Kahl, 1932) gen. nov., comb. nov.**

**Fig. 6:**
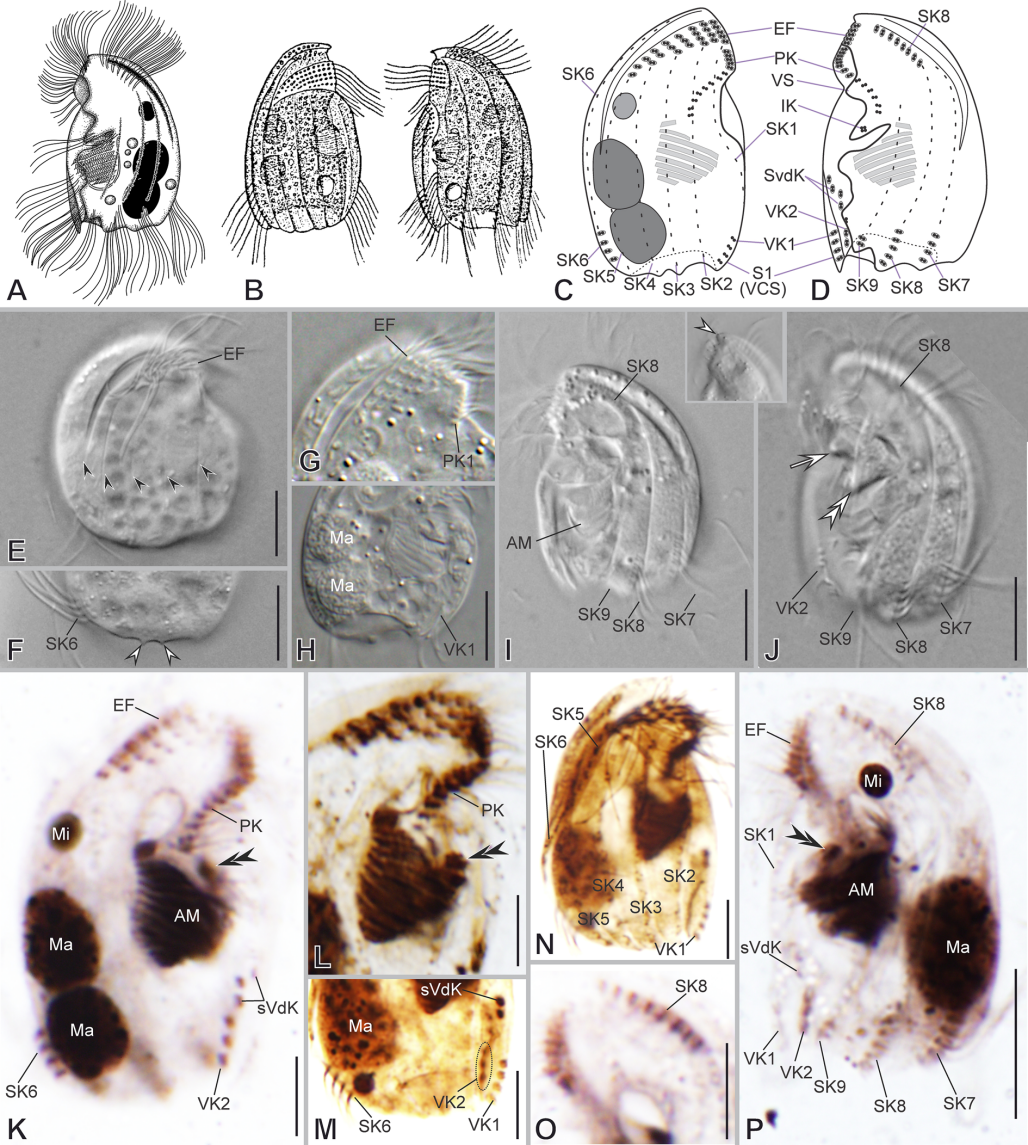
Morphology of *Mircalla triangula* gen. nov., nov. comb. in vivo (**A**, **B**, **E**–**J**) and after impregnation (**C**, **D**, **K**–**P**). **A**–**D**, **I**–**K**, **N**–**P** General cell shape, frontal spine (white arrowhead), ventral spine (white arrow), and lapel-like structure (white double arrowhead); infraciliature showing somatic kineties (*SK*) in an *Epalxella* pattern, macronuclear nodules (*Ma*), micronucleus (*Mi*), and adoral membranelles (*AM*). **B** After Kahl, 1932a, b. **E**, **F** Compressed cell showing non-ciliated segments of *SKs* (black arrowheads) and posterior spines (white arrowheads). **G**, **L** Detail of epistomial fringe (*EF*), preoral kinety (*PK*), and inverse kinety (*IK*, black double arrowhead). **H**, **M** Detail of ventral kineties (*VK*), supplementary ventral dikinetids (*sVdK*), and the ciliated spine segment of *SK6*. Scale bars: 10 µm

***Redescription based on EPOPOLO population*** (Fig. 6; Supplementary Table S8): Cells 32–37 × 20–27 μm in vivo and 25 × 16 μm on average in protargol-impregnated specimens. The shape is almost triangular in outline (Fig. 6A–D, I, J). The frontal spine is short and inconspicuous (Fig. 6B, D, I); the dorsal keel is folded slightly rightwards, forming a shallow trough ending equatorially (Fig. 6C, E, G). About seven blunt, inconspicuous, inwardly curved spines surround a concavity at the posterior cell margin, except for the ventrocaudal spine, making the posterior margins wavy (Fig. 6C, D, F, H, I, J). The lapel-like structure enfolds the two dikinetids of the inverse kinety (Fig. 6D, J, K, P). An inconspicuous fang-like spine is on the ventral margin toward the left side, between the anterior end of the preoral kinety and the lapel-like structure (Fig. 6A, D, J). One or two macronuclear nodules and a single micronucleus (Fig. 6C, H, K, M, P).

On average, the continual epistomial fringe comprises seven pectinelles, mostly on the right side (Fig. 6C, K, L). A single preoral kinetiy, PK, with an average of 13 dikinetids (Fig. 6C, D, K, L). The two ventral kineties (VK1–2) have an average of four and three dikinetids, respectively, and lie in the ventrocaudal spine; the VK1 is more superficial than the VK2. Two pairs of supplementary ventral dikinetids are anterior to the VK2 (Fig. 6C, D, K, M, P). The oral cavity invariably has nine membranelles, eight within the outer compartment and the ninth in the inner (Fig. 6C, D, K, L, N, P).

The somatic kineties are arranged in an *Epalxella* pattern (Fig. 6C, D, I–K, M, N, P). The SK1 ends ventrally. The SK2–5 ends posteriorly, likely in the reduced spines. The SK6 ends posteriorly with four ciliated dikinetids (Fig. 6C, F, K, M). The SK8 begins with seven ciliated dikinetids (Fig. 6D, I, J, O, P). The SK7, 8, and 9 end in reduced spines with an average of five, four, and two dikinetids, respectively (Fig. 6D, I, J, P).

### ***Epalxella exigua* (Penard, 1922) Corliss, 1960**

**Fig. 7:**
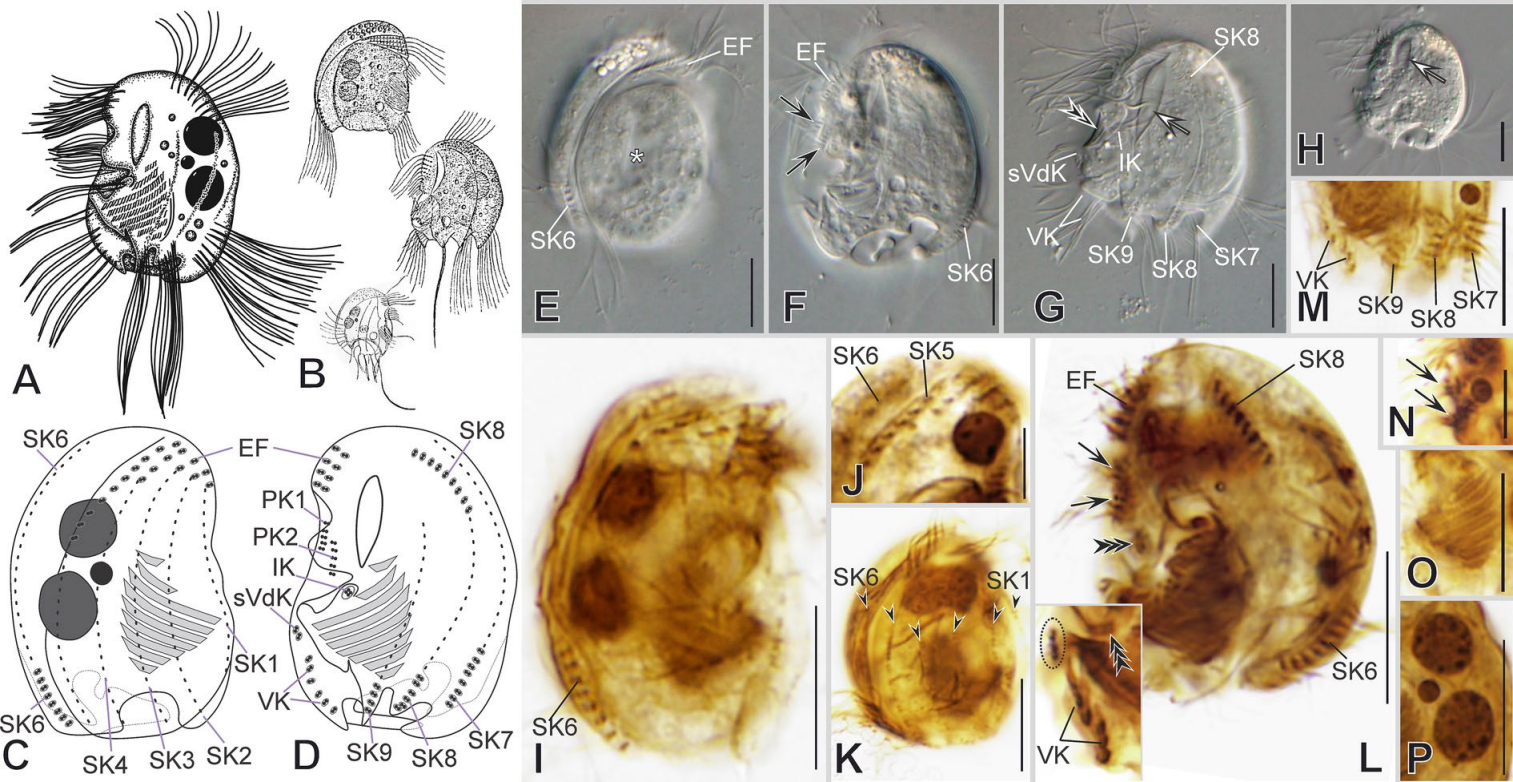
Morphology of *Epalxella exigua* in vivo (**A**, **B**, **E**–**H**) and after impregnation (**C**, **D**, **I**–**P**). **A**, **B**, **E**, **F**–**H** General cell shape, window-like cortical slit (white arrows), vaulted lateral side (asterisk), lapel-like structure (black double arrowhead). **B** After Kahl, 1932a, b, and Penard, 1922. **C**, **D**, **I**, **K**–**M** Infraciliature showing somatic kineties (*SK*, black arrowheads) in an *Epalxella* pattern, inverse kinety (*IK*, black triple arrowheads), ventral kinety (*VK*), and supplementary ventral dikinetids (*sVdK*, dotted circle). **J** Detail of the epistomial fringe (*EF*). **N** Preoral kineties (*PK*¸black arrows). **O** Adoral membranelles. **P** Nuclear apparatus. Scale bars: 10 µm

***Redescription based on LAUFENJ population*** (Fig. 7; Supplementary Table S9): Cells 33 × 25 μm in vivo and 25 × 17 μm in protargol-impregnated specimens on average. The shape is almost semicircular to ellipsoidal in outline (Fig. 7A–H). The frontal spine is absent; the ventro-frontal edge is blunt; the dorsal keel is fully folded rightwards, forming a deep through (Fig. 7B, C, E). On the left side, the cortex bears a conspicuous window-like cortical slit (Fig. 7A, B, D, G, H). Short, inconspicuous spines surround a concavity at the posterior cell margin; three of them are visible on the left side, whereas the remaining are on the right side and inwardly curved, except for the ventrocaudal spine, making the posterior right margin wavy (Fig. 7A–E, G). The lapel-like structure enfolds the two dikinetids of the inverse kinety (Fig. 7D, G, L). One or two macronuclear nodules and a single micronucleus (Fig. 7A–C, I, P).

On average, the continual epistomial fringe comprises eight pectinelles, mostly on the right side (Fig. 7B, C, E, I–K). The two preoral kineties (PK1–2) have an average of five and three dikinetids, respectively (Fig. 7D, F, L, N). One ventral kinety (VK) is located in the internal side of the ventrocaudal spine; the kinety is fragmented into two groups of three dikinetids. Above the VK is one pair of supplementary ventral dikinetids (Fig. 7D, G, L, M). The oral cavity invariably has nine membranelles, eight within the outer compartment and the ninth in the inner (Fig. 7C, D, F, I, L, O).

The somatic kineties are arranged in an *Epalxella* pattern (Fig. 7C, D, K, L, M). The SK1 ends ventrally. The SK2–5 ends posteriorly, likely in the reduced spines. The SK6 ends in a reduced spine with eight ciliated dikinetids (Fig. 7C, E, I, K, L). The SK8 begins with eight ciliated dikinetids (Fig. 7D, G, L). The SK7, 8, and 9 end in reduced spines with an average of eight, six, and four dikinetids, respectively (Fig. 7D, G, M).

***Notes on 18S rRNA gene sequences:*** The genetic distance (uncorrected p distance) between the two studied populations was 0.01 (Supplementary Table S4).

### ***Epalxella* cf. *antiquorum***

**Fig. 8:**
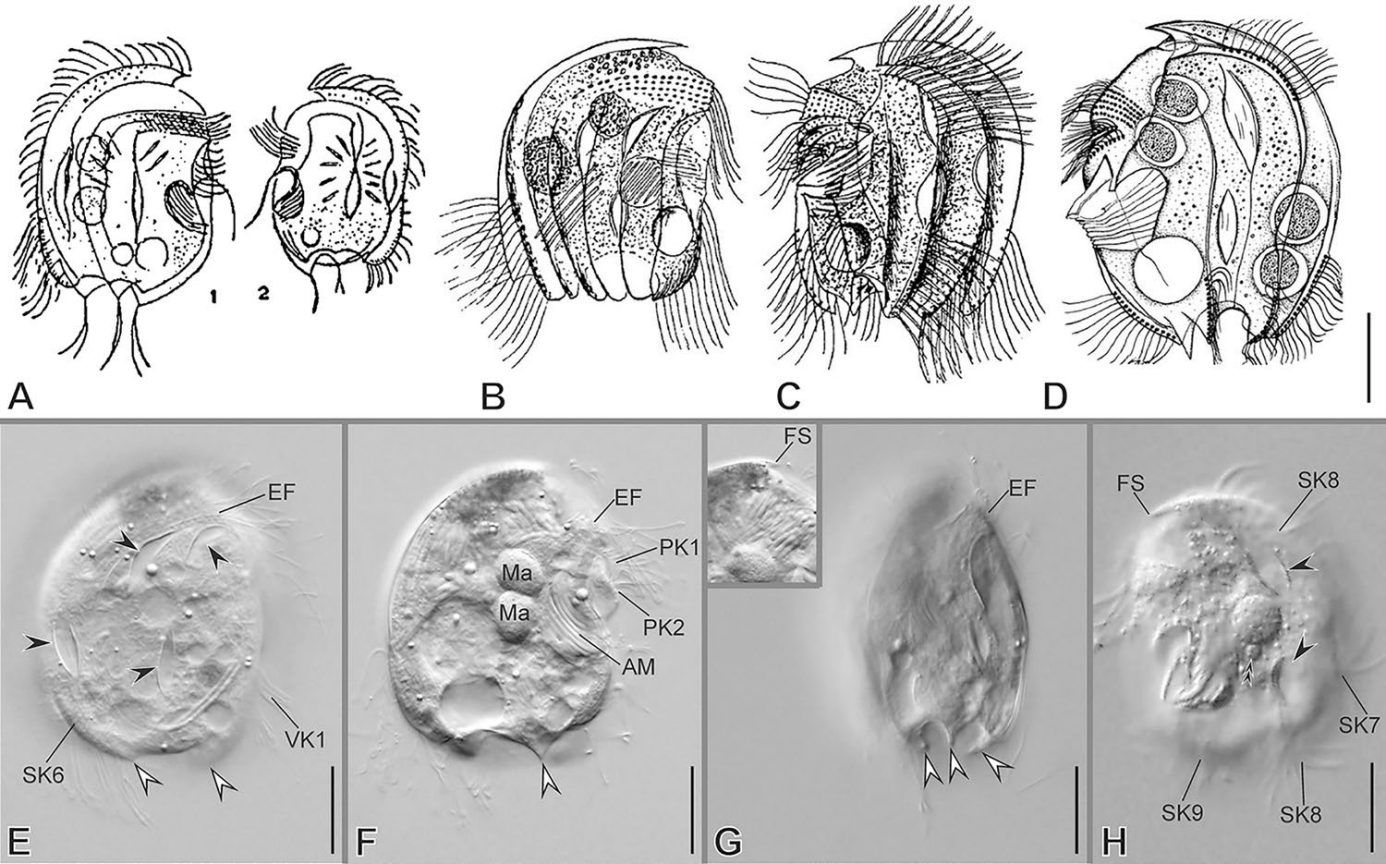
*Epalxella antiquorum* (**A**–**D**) and *Epalxella* cf. *antiquorum* (**E**–**H**) in vivo. After Penard, 1922 (**A**), Kahl, 1932a, b (**B**, **C**), and Jankowski 1964 (**D**). **E**–**H** General morphology of *Epalxella* cf. *antiquorum* showing window-like cortical slits (black arrowheads), posterior spines (white arrowheads), frontal spine (*FS*), epistomial fringe (*EF*), adoral membranelles (*AM*), macronuclei (*Ma*), micronucleus (black double arrowhead), preoral kineties (*PK*), somatic kineties (*SK*), and ventral kinety (*VK*). Scale bars: 20 µm

***Notes on the observed MOKOT populations*** (Fig. 8; Supplementary Video S4): Cells about 64 × 46 μm in vivo on average (*n* = 3). The shape is ellipsoidal to quadrangular in outline. The frontal spine is present (Fig. 8G, inset). Short, inconspicuous spines surround a concavity at the posterior cell margin; three or four are visible on the left side, whereas the remaining ones are inwardly curved on the right side, except for the ventrocaudal spine, making the posterior right margin wavy. The cortex bears window-like holes, four on the right and two on the left sides. The epistomial fringe is continuous, starting on the left side and ending with staggered rows on the right side. One-to three macronuclear nodules and a single micronucleus.

***Notes on 18S rRNA gene sequences:*** Three sequences were obtained, two from MOKOT (PQ699755, PQ699757), and one from ZLAKOR2A (PQ699763). The genetic distance (uncorrected *p* distance) between the two studied populations is 0.001 (Supplementary Table S4).

### ***Pelodinium reniforme* Lauterborn, 1908**

**Fig. 9:**
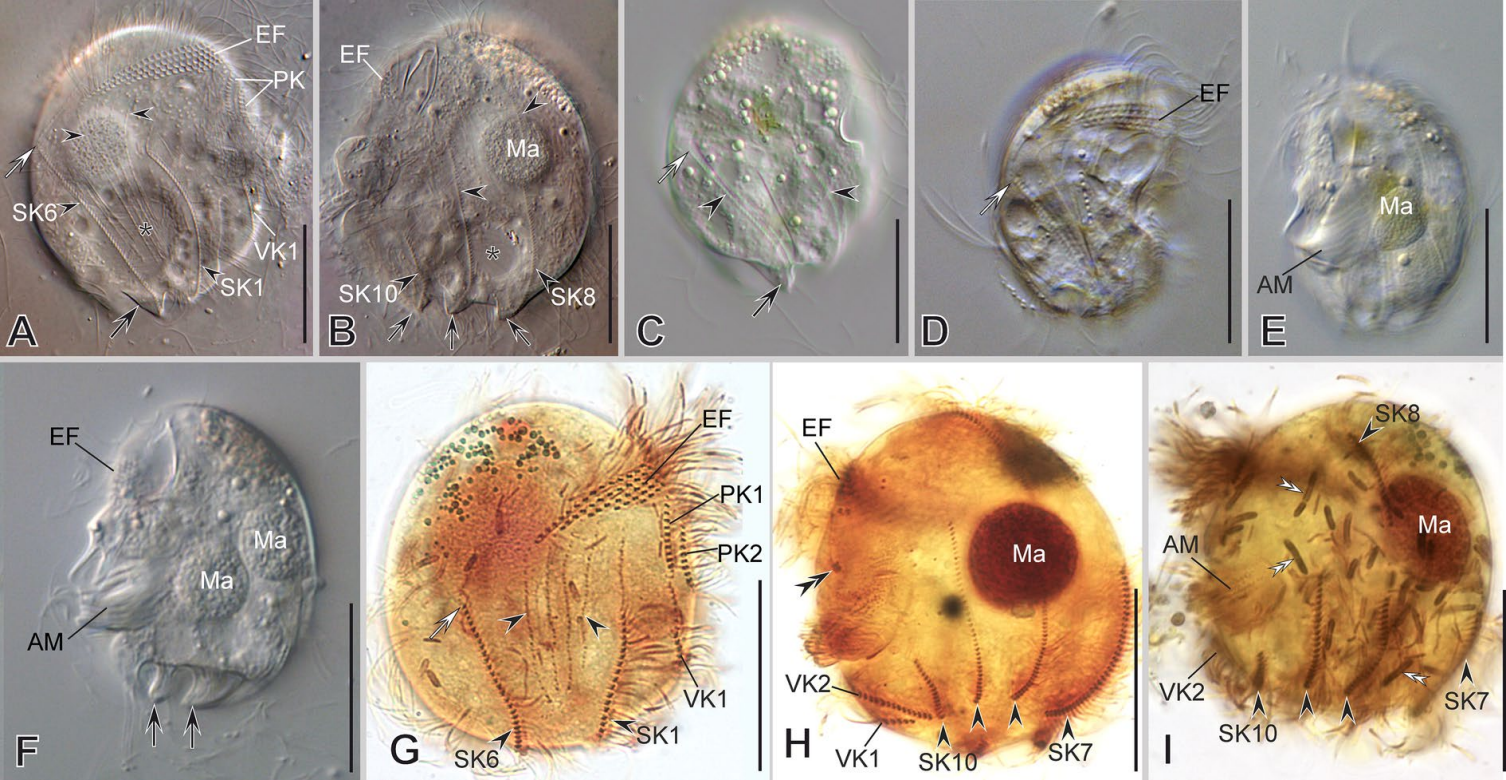
Morphology of *Pelodinium reniforme* in vivo (**A**–**F**) and after silver carbonate impregnation (**G**–**I**). **A**–**I** General cell shape, posterior spines (black arrows), and infraciliature showing the somatic kineties (*SK*, black arrowheads) in a *Pelodinium* pattern, *SK6* and *SK5* of epistomial fringe (*EF*) form a 90° angle (white arrows). Macronucleus (*Ma*), preoral kineties (*PK*), ventral kineties (*VK*), inverse kinety (black double arrowhead), adoral membranelles (*AM*), contractile vacuole (asterisks), and prokaryotic endosymbionts (white double arrowheads). Scale bars: 25 µm

***Notes on the observed MOKOTPEL and GLENWOODP populations ***(Fig. 9): Cells about 50 × 36 μm in vivo on average. The shape is ellipsoidal in outline. The frontal spine is absent, and a ventral spine is present (Fig. 9E, F). Around six blunt spines border the posterior marginal heart-shaped concavity (Fig. 9A–C, F). There are two preoral kineties (PK1 and PK2 with 9–15 and 11–14 dikinetids, respectively, *n* = 4) and two ventral kineties (VK1 and VK2 with 15 and 13 dikinetids, respectively, *n* = 1) (Fig. 9G, H). There are ten somatic kineties arranged in the *Pelodinium* pattern, including the epistomial rows (Fig. 2P), six of them (SK1, 6–10) end with long ciliated spine segments (Fig. 9G–I). The epistomial fringe is continuous with 15–20 pectinelles (*n* = 3), starting on the ventral margin and ending with staggered rows on the right side. The non-ciliated segments of the epistomial fringe (SK1–5) and the SK6 are folded posteriorly, forming an almost 90° angle (Fig. 9A, C, D, G). The SK1 and SK6 end with long ciliated spine segments posteriorly with 18–31 (*n* = 4) and 23–36 (*n* = 4) dikinetids, respectively, whereas the non-ciliated segments of SK2–5 end subposterioly without ciliated segments (Fig. 9G). The SK8 begins with 24–33 ciliated dikinetids (*n* = 2). The SK7, 8, and 9 end posteriorly with 35 (*n* = 1), 22–38 (*n* = 2), and 22–38 (*n* = 2) dikinetids, respectively (Fig. 9H, I). The short SK10 is located on the ventral margin at the posterior third of the cell and includes a ciliated segment solely with around 15 dikinetids (*n* = 1) (Fig. 9H, I). The inverse kinety comprises about two dikinetids (Fig. 9H). There are one or two macronuclear nodules and a single micronucleus (Fig. 9A, B, E, F, H).

### **Odontostomatean sp. GROSERDU**

**Fig. 10:**
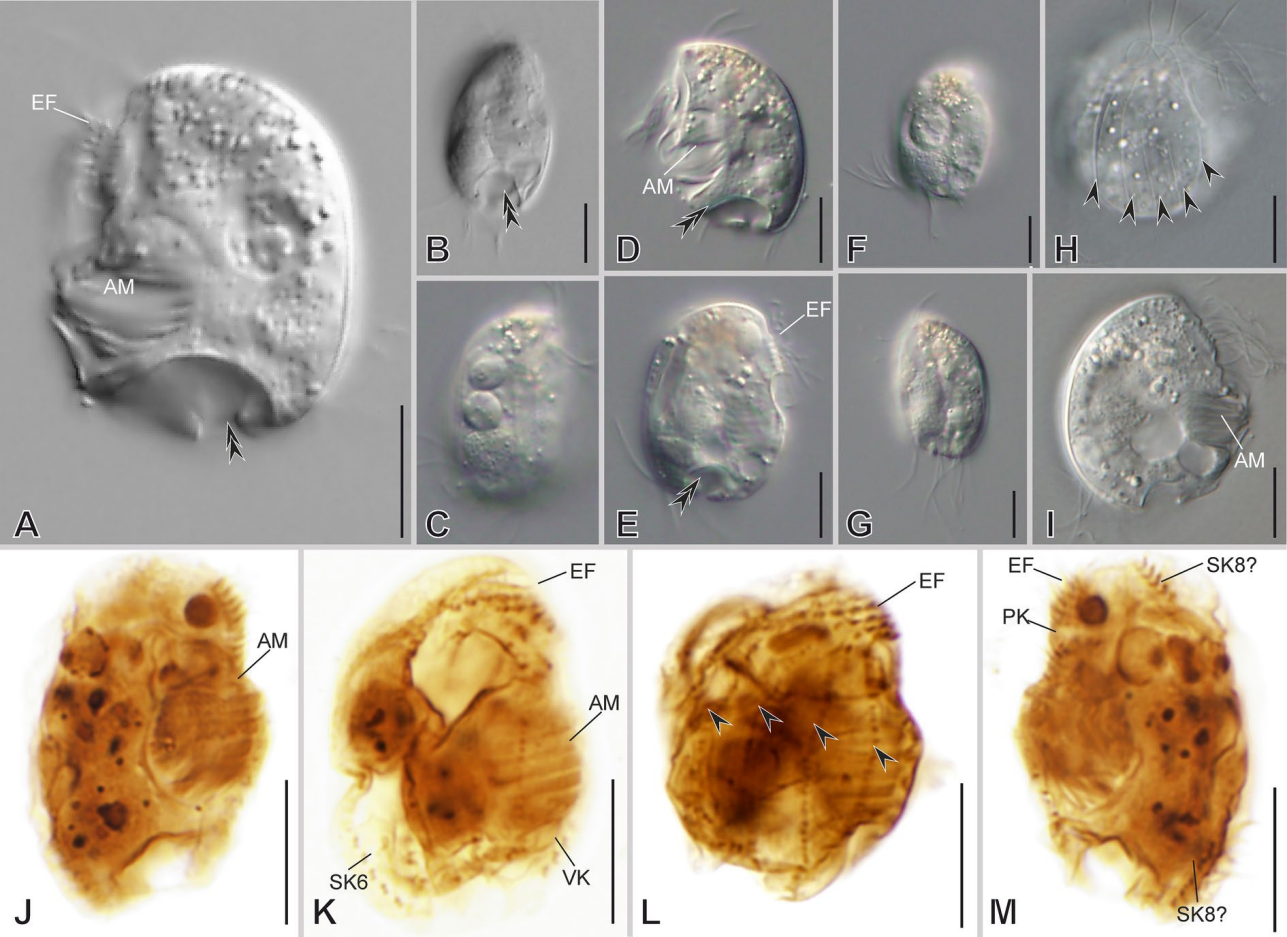
Odontostomatea sp. (GROSERDU population) in vivo (**A**–**I**) and after impregnation (**J**–**M**). **A**–**I** General cell shape in different focal points. **J**–**M** Infraciliature after protargol impregnation. **H**, **L** Right lateral views showing the non-ciliated segments of *SK1*-5 (black arrowheads). *AM* adoral membranelles, *EF* epistomial fringe, *PK* preoral kinety, *SK* somatic kinety, *VK* ventral kinety. Scale bars: 10 µm

***Notes on the observed population*** (Fig. 10): Cells 32 × 24 μm in vivo and 25 × 17 μm in protargol-impregnated specimens on average. The shape is almost circular to ellipsoidal in outline (Fig. 10A–I). The frontal spine is absent. A concavity at the posterior cell margin is surrounded by short, inconspicuous spines on the left side; the posterior right margin is smooth (Fig. 10C, D, F). Details of the infraciliature could not be determined due to the suboptimal quality of the protargol preparations.

***Notes on 18S rRNA gene sequences and voucher material:*** Two sequences were obtained (PQ699741–PQ699742). One slide containing protargol-impregnated specimens is deposited in the National Museum of the Czech Republic, Prague, Czech Republic (catalog number P6E 5620).

### ***Mylestoma bipartitum* (Gourret & Roeser, 1886) Kahl, 1928**

**Fig. 11:**
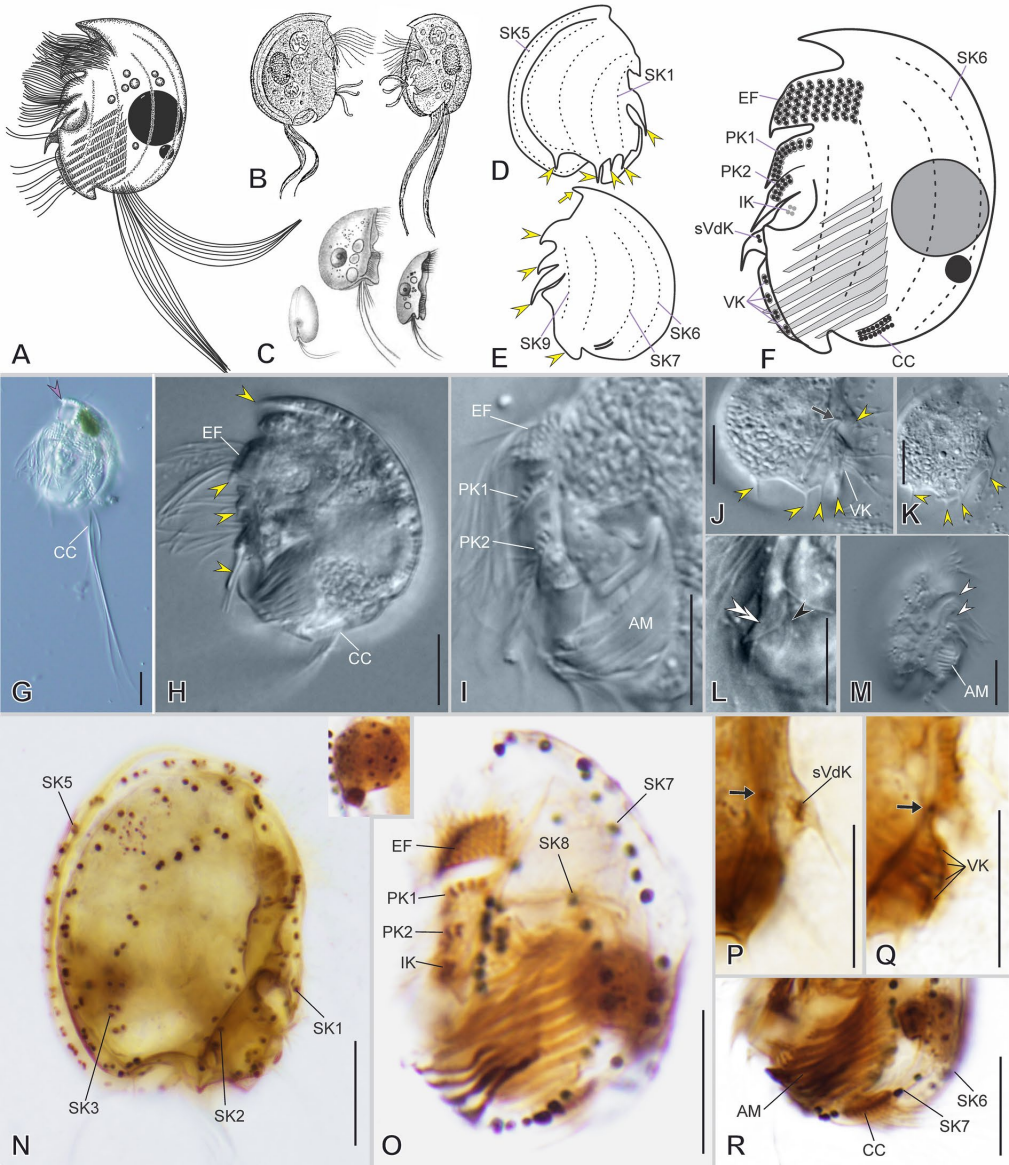
Morphology of *Mylestoma bipartitum* in vivo (**A**–**C**, **G**–**M**) and after impregnation (**D**–**F**, **N**–**R**). **A**, **D**, **E**, **G**, **H** General cell shape and distribution of spines (yellow arrowheads). After Kahl 1928 (**B**), and Gourret and Roeser 1886 (**C**). **F**, **I**, **N**, **O** Infraciliature showing somatic kineties (*SK*) in a *Mylestoma* pattern, nuclear apparatus (inset in **N**), epistomial fringe (*EF*), preoral kineties (*PK*), and adoral membranelles (*AM*). **J**, **K** Left lateral views showing ventral and posterior spines (yellow arrowheads). **L** Ventral margin showing lapel-like structure (white double arrowhead) and inverse kinety (*IK*, black arrowhead). **M** Furrows of *PK* (white arrowheads) and *AM*. **P**, **Q** Ventral margin showing supplementary ventral dikinetids (*sVdK*) on a ventral spine, and ventral kinety (*VK*). **R** Detail of *AM* and the semikinetal arrangement of caudal cirri (*CC*). Scale bars: 10 µm

***Redescription based on KLAGOS population*** (Fig. 11; Supplementary Table S10): Cells 33–42 × 24–31 μm in vivo and 30 × 21 μm on average in protargol-impregnated specimens. The shape is semicircular in outline (Fig. 11A–H, N, O). Anteriorly, the cortex ends ventrally as a hood-like structure resembling a spine in lateral view and is continuous with the dorsal keel (Fig. 11B, D, N). The right side of the cell is distinctly vaulted with two conspicuous and two less conspicuous sharp spines on the posterior margin (Fig. 11B, D, J). The left side is more or less flattened, with a blunt spine on the posterior ventral margin. Ventrally, anterior to the buccal area, there are two conspicuous sharp spines, one on the right side and the other on the left side (Fig. 11D–F, H, J, K, P); two short spines protrude at the end of the ventral ridge and the base of the preoral kinety 1 (Fig. 11E, F, H). The lapel-like structure enfolds the two dikinetids of the inverse kinety (Fig. 11F, L, O). One macronuclear nodule and a single micronucleus (Fig. 11A, F, N inset).

On average, the continual epistomial fringe comprises eight pectinelles, which mostly lie on the left side (Fig. 11F, H, I, O). The two preoral kineties (PK1–2) have an average of 10 and seven dikinetids, respectively (Fig. 11F, I, O). One ventral kinety (VK1) is located in the rightmost plate of the buccal area, having about four dikinetids plus one additional supplementary ventral dikinetid (Fig. 11F, J, P, Q). The oral cavity invariably has nine membranelles, eight within the outer compartment and the ninth in the inner (Fig. 11A, F, H, I, M, O, R). The right ventral spine bears at its base one pair of long cilia (Fig. 11J, P, Q).

The somatic kineties are arranged in the *Mylestoma* pattern consisting of nine non-ciliated kineties, five on the right side and four on the left side (Fig. 11D, E, F, N, O). Two caudal cirri, located at the posterior end of SK7 and composed of 10 kinetids each, bear long cilia (44 μm in vivo), forming a semikinetal pattern (Fig. 11A, B, E–H, R).

### ***Mylestoma monodontum* sp. nov.**

**Fig. 12:**
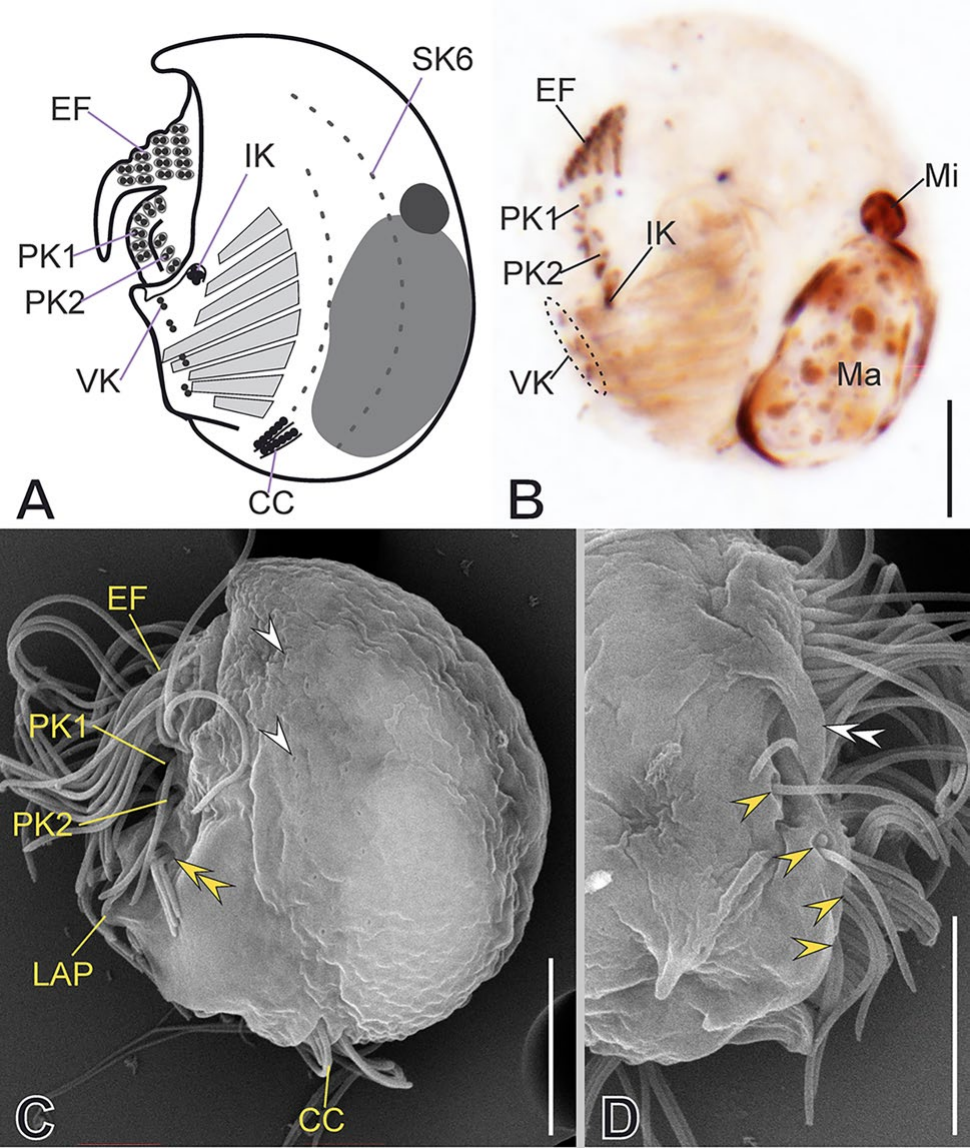
Morphology of *Mylestoma monodontum* sp. nov. after impregnation (**A**, **B**) and SEM (**C**, **D**). **A**–**C** Infraciliature showing epistomial fringe (*EF*), preoral kineties (*PK*), lapel-like structure (*LAP*), inverse kinety (*IK*, yellow double arrowhead), ventral kinety (*VK*), somatic kineties (*SK*, white arrowheads), and semikinetal arrangement of caudal cirri (*CC*). Macronucleus (*Ma*) and micronucleus (*Mi*). **D** Detail of ventral margin showing dikinetids of *VK* (yellow arrowheads) and ventral spine (white double arrowhead). Scale bars: 10 µm

***Additional notes to the description FARO population*** (Fig. 12; Supplementary Table S10, Video 5): Anteriorly ending as a hood-like structure resembling a spine in lateral view. One conspicuous, fang-like spine emerges at the end of the epistomial fringe on the right ventrolateral side. An inconspicuous spine protrudes from the posterior ventral left side. The lapel-like structure is present (labeled as the oral spine in Méndez-Sánchez et al. 2023) and enfolds the two dikinetids of the inverse kinety (Fig. 12A–C). Five somatic kineties (SK1–5) on the right side and two (SK6–7) on the left side are arranged in the *Mylestoma* pattern. Two caudal cirri, located at the posterior end of SK7 and composed of six kinetids each, bear long cilia (25 μm in vivo), forming a semikinetal pattern (Fig. 12A, C).

***Notes on 18S rRNA gene sequences:*** The genetic distance (uncorrected *p* distance) between the FARO sequences and the previously published FRESHO (OP034695-9) ranges from 0 to 0.004 (Supplementary Table S4).

### ***Limnomylestoma shuriken* gen. nov., sp. nov.**

**Fig. 13:**
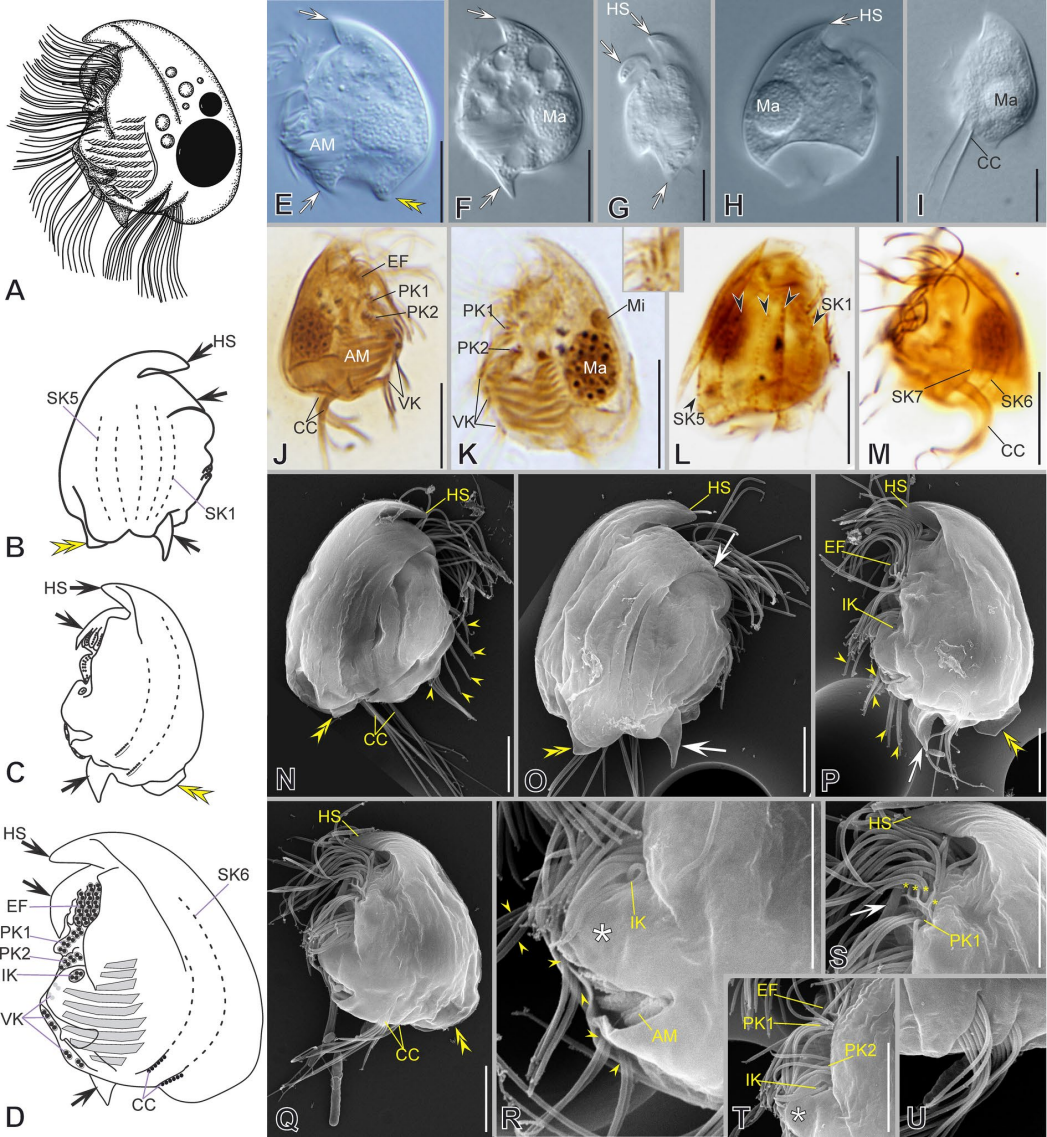
Morphology of *Limnomylestoma shuriken* nov. sp. in vivo (**A**, **E**–**I**), after impregnation (**B**–**D**, **J**–**M**) and in SEM (**N**–**U**). **A**–**C**, **E**–**H**, **O**, **Q** General cell shape and distribution of hood-like structure (*HS*), spines (white and black arrows), and fin-like projection (yellow double arrowhead). **D**, **K**, **L**, **N**, **P** Infraciliature showing somatic kineties (*SK*) in a *Mylestoma* pattern, and macronucleus (*Ma*) and micronucleus (*Mi*). **J**, **R**–**T** Detail of epistomial fringe (*EF*, inset in K, and yellow asterisks in S), preoral kineties (*PK*), inverse kinety (*IK*), lapel-like structure (white asterisks), dikinetids of ventral kinety (*VK*, yellow arrowheads), and adoral membranelles (*AM*). **I**, **M**, **U** Amphikinetal arrangement of the caudal cirri (*CC*). Scale bars: 10 µm

***Description based on SLATINA population*** (Fig. 13; Supplementary Table S10, Video S6): Cells 24–33 × 18–26 μm in vivo and 19 × 15 μm on average in protargol-impregnated specimens. The shape is semicircular in outline with two or three prominent spines (Fig. 13A–H, N–Q). Anteriorly, the cortex ends ventrally as a hood-like structure resembling a spine in lateral view and is continuous with the dorsal keel, which is fused to the right lateral side (Fig. 13B, N, O). There is a ventral spine, which is an extension of the right lateral side of the cortex at the level of the epistomial fringe (Fig. 13A–D, G, S). The caudal spine arises on the right side and is sometimes inconspicuous (Fig. 13B–G, O, P). A fin-like projection is present on the posterior right margin (Fig. 13B, C, E, N–Q). The lapel-like structure appears as a flattened lamina (e.g., not folded) and enfolds the two dikinetids of the inverse kinety (Fig. 13D, P–R, T). One spherical macronucleus and a single micronucleus (Fig. 13A, F, H, I, J, K).

On average, the continual epistomial fringe comprises four pectinelles on the ventral margin (Fig. 13D, J, K, P, S). Two preoral kineties, PK1 and PK2, with an average of five and three dikinetids, respectively (Fig. 13D, J, K, S, T). One ventral kinety, VK, is located on the rightmost plate of the buccal area having six dikinetids on average (Fig. 13D, J, K, N, P, R). The oral cavity invariably has nine membranelles, eight within the outer compartment and the ninth in the inner (Fig. 13D, E, J, K, R).

The somatic kineties are arranged in the *Mylestoma* pattern, consisting of seven non-ciliated kineties, five on the right side and two on the left side (Fig. 13B–D, J–M). Two caudal cirri, one each at the posterior end of SK6 and SK7, and composed of eight kinetids each, bear long cilia (20 μm in vivo), forming an amphikinetal pattern (Fig. 13D, J, M, P, Q, U).

### ***Tostonella uncinata* (Penard, 1922) gen. nov., comb. nov.**

**Fig. 14:**
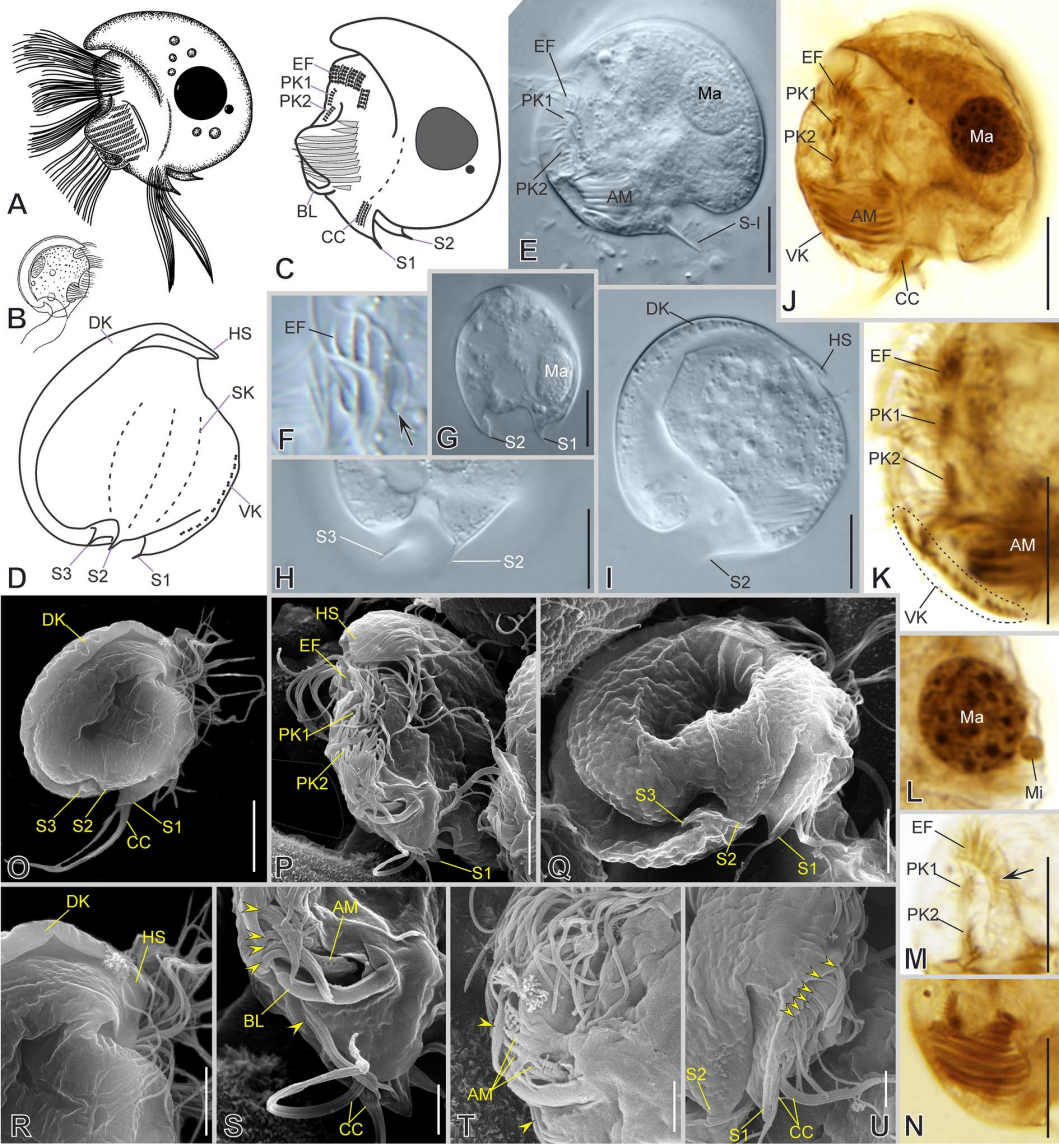
Morphology of *Tostonella uncinata* gen. nov., comb. nov. in vivo (**A**, **B**, **E**–**I**), after impregnation (**C**, **D**, **J**–**N**), and in SEM (**O**–**U**). **A**–**D**, **E**, **I**–**K**, **O**, **P** General cell shape and infraciliature. **B** After Penard 1922. **G**, **H**, **Q** Detail of posterior spines (*S*), and **G** shows a longitudinal crosssection in ventral view. **F**, **M** Detail of epistomial fringe (*EF*), displacement of first pectinelle (black arrows), and preoral kineties (*PK*). **L** Macronucleus (*Ma*) and micronucleus (*Mi*). **N** Adoral membranelles (*AM*). **R** Anterior cell portion showing dorsal keel (*DK*) and hood-like structure (*HS*). **S**–**U** Detail of ventral kinety (*VK*, yellow arrowheads), *AM*, buccal lip (*BL*), spines, and caudal cirri (*CC*). Scale bars: 10 µm

***Redescription based on BUL7A population*** (Fig. 14; Supplementary Table S11, Video S7): Cells 28–34 μm in diameter in vivo and 28 μm on average in protargol-impregnated specimens. The shape is circular in outline with three prominent posterior spines (Fig. 14A–D, E, H–J, O, Q). Anteriorly, the cortex ends ventrally as a hood-like structure resembling a spine in lateral view (Fig. 14D, O, P, R), and the dorsal keel is slightly folded rightwards, forming a shallow trough and posteriorly ends as the backward-folded spine 3 (Fig. 14B, D, I, O, Q). The right side of the cell is more or less flattened, whereas the left side is vaulted (i.e., strongly convex). Two parallel, posteriorly directed, sharp spines emerge from the posterior ventral cell margin (Fig. 14C, D, G, Q). The lapel-like structure and the inverse kinety are absent. One macronuclear nodule and a single micronucleus (Fig. 14A, C, E, G, J, L).

On average, the continual epistomial fringe comprises five pectinelles on the ventral margin; the left-most pectinelle is posteriorly displaced (Fig. 14C, F, M). Two preoral kineties, PK1 and PK2, with an average of seven dikinetids each, respectively (Fig. 14C, E, K, M, P). One ventral kinety, consisting of ten dikinetids on average, is located on the rightmost side of the buccal area (Fig. 14D, J, K, S–U). The oral cavity invariably has ten membranelles, nine within the outer compartment and the tenth in the inner (Fig. 14A, C, E, J, K, N, S, T).

Somatic kineties are not studied in detail, but apparently, there are three non-ciliated kineties on the right side and one on the left side (Fig. 14C, D). Two caudal cirri, with about seven kinetids each, lie on the left-most posterior spine (S1); one of them apparently continuous with the somatic kinety on the left side; the caudal cirri bear long cilia (about 25 μm in vivo) (Fig. 14C, J, O, S, U).

***Notes:*** The material obtained from the CEDARKOR population was insufficient to describe it. However, the cell shape was identical to that of the cells from the BUL7A population.

***Notes on 18S rRNA gene sequences:*** The genetic distance (uncorrected *p* distance) between both populations is 0.

### ***Discomorphella pectinata* (Levander, 1894) Corliss, 1960**

**Fig. 15:**
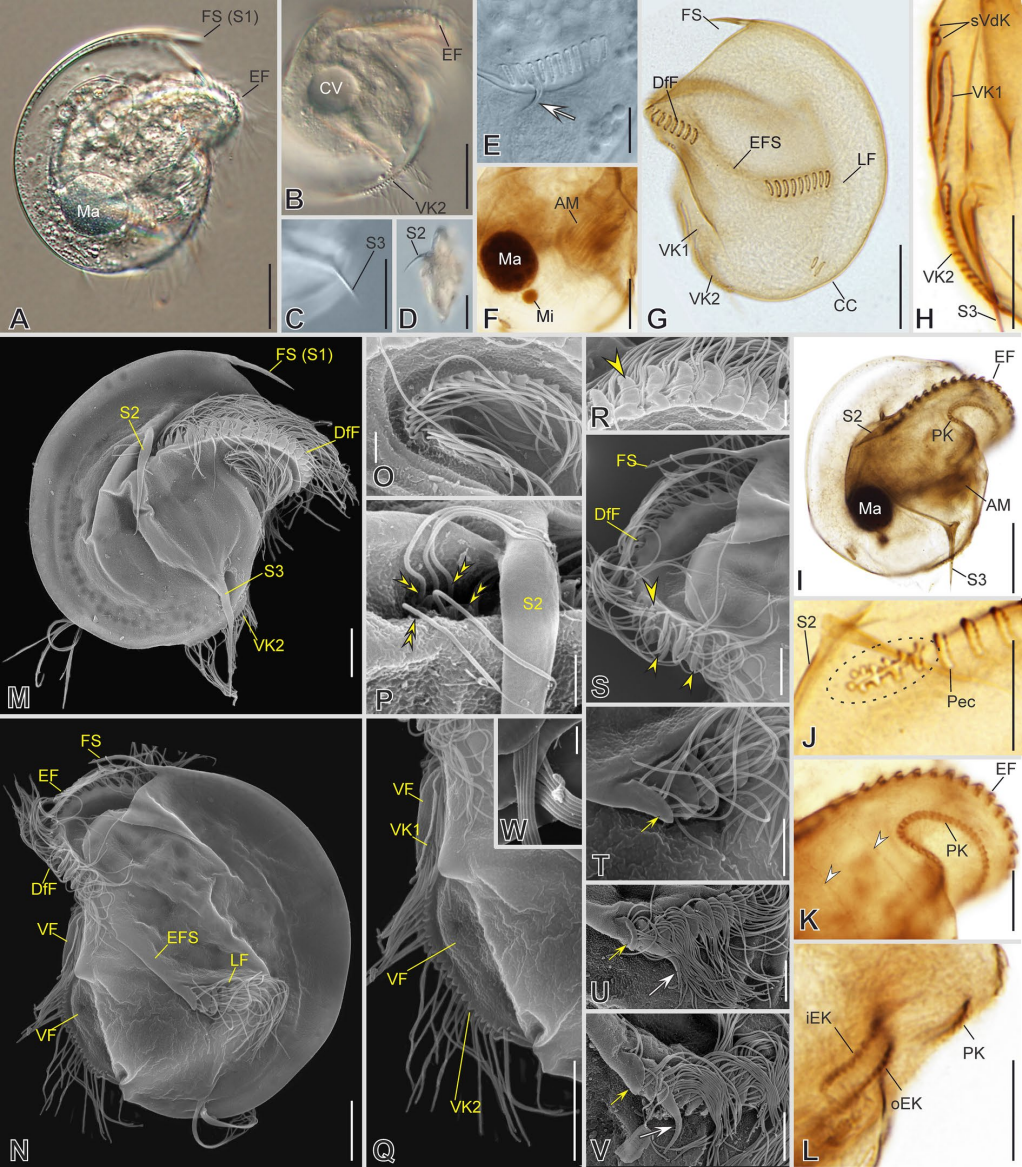
Morphology of *Discomorphella pectinata* in vivo (**A**–**E**), after impregnation (**F**–**L**), and in SEM (**M**–**W**). **A**, **M**, **G**, **I**, **N** General cell shape and infraciliature. Epistomial fringe (*EF*) fragmented into dextro-frontal fragment (*DfF*) and left fragment (*LF*), both separated by an epistomial fringe spacer (*EFS*). **C**, **D** Posterior and lateral spines (*S*). **F** Macronucleus (*Ma*), micronucleus (*Mi*), and adoral membranelles (*AM*). **B**, **H**, **Q** Detail of ventral kineties (*VK*), supplementary ventral dikinetids (*sVdK*), serrated ventral flat (*VF*), and contractile vacuole (*CV* in B). **E**, **T**–**V** Arrangement of *LF*, first pectinelle (*Pec,* yellow arrow), and associated *LF*-associated spine (white arrows). **J**, **P** Detail of papillary dikinetids at the end of *DfF* (encircled in **J** and yellow double arrowheads in **P**). **K**, **O** Detail of preoral kinety (*PK*), and non-ciliated segments of somatic kienties (white arrowheads in **K**). **R**, **S** Detail of *DfF*, pectinelles (yellow arrowheads), and frontal spine (*FS*). **L** Inner and outer endoral kineties (*iEK*, *oEK*, respectively). **W** Detail of caudal cirri (*CC*). Scale bars: 20 µm (**A**–**D**, **G**, **I**), 10 µm (**E**, **F**, **H**, **J**–**N**, **Q**), 2 µm (**O**, **P**, **R**, **W**), 5 µm (**S**, **T**–**V**)

***Redescription based on MOKOT, BOTAN, and LAPLICE populations ***(Fig. 15; Supplementary Table S12, Video S8): Cells 67–79 μm in diameter in vivo and 63 μm on average in protargol-impregnated specimens. Shape circular in outline, the right side of the cell is vaulted, and the left side is flattened. Three long, sharp spines protrude from the cortex, one frontal spine and two spines on the right side: one above the end of the dextro-frontal epistomial fragment and one on the posterior region of the vaulted surface (Fig. 15A, C, D, G, I, M, N). The lapel-like structure and the inverse kinety are absent. One spherical macronuclear nodule and a single micronucleus (Fig. 15A, F, I).

The epistomial fringe resides on a conspicuously protruding hoop-like frontal awning and fragmented into a dextro-frontal and a left fragment linked by the epistomial fringe spacer (Fig. 15A, B, E, G, M, N). Pectinelles are armored with auricules (Fig. 15R, T–V). The dextro-frontal fragment forms a frontal awning and bears 21 pectinelles, the left fragment with nine pectinelles, on average; each pectinelle in the left fragment bears four ciliated dikinetids, whereas the dextro-frontal fragment starts with five and ends staggered with three (Fig. 15J, R, S). The auricule of the second or third pectinelle on the left fragment might extend forming a small spine, but not always present (Fig. 15E, N, T–V). The dextro-frontal fragment ends with about 11 papillary dikinetids (Fig. 15J, P). The preoral kinety is hook-like, sinistrally oriented below the frontal awning, with 25 dikinetids, which are armored with small pectinelles (Fig. 15K, O). Posterior to the frontal awning, in the most external part of the buccal cavity, two endoral kineties, outer and inner, lie in parallel to each other obliquely to the cell long axis and consist of 12 and 13 dikinetids, respectively (Fig. 15L). Adoral zone with nine obliquely oriented membranelles (Fig. 15F) decreasing in length toward each end of the adoral zone. There are two supplementary ventral kineties slightly anterior to the cell equator (Fig. 15H). Posterior to, there is a serrated ventral flap in two sections, each bearing a ventral kinety consisting of 16 and 20 dikinetids, respectively (Fig. 15B, H, N, Q).

The somatic kineties are arranged in a *Discomorphella* pattern, with seven kineties on the right and three on the left side (Fig. 2R). Two caudal cirri, one each at the posterior end of SK9 or SK10 and composed of about seven kinetids each, bear long cilia (20 μm in vivo) (Fig. 15G, N, W).

***Notes:*** The morphometric values given in the above description are based on the MOKOT population. Refer to Table S12 for the morphometric values of the BOTAN population. The BOTAN and MOKOT populations are highly similar. Only a few differences were notable. For instance, the epistomial fringe spacer in the MOKOT population was longer than in the BOTAN population (25 µm vs. 17 µm, on average).

***Notes on 18S rRNA gene sequences:*** The genetic distance (uncorrected p distance) between the three studied populations ranges from 0 to 0.001 (Supplementary Table S4).

## Discussion

### Odontostomatea from a phylogenetic point of view

Odontostomatean ciliates were historically considered to belong to several different groups, e.g., Heterotrichida Stein, 1859, Plagiopylea, and Armophorea (Adl et al. 2019; Jankowski 1964b, 2007; Kahl 1932b; Lynn 2008; Stoeck et al. 2007), until the establishment of their own class (Fernandes et al. 2018). The newly sequenced odontostomatean species strongly support the monophyly of the Odontostomatea (Fig. 1).

Our data provide clear evidence that the problematic genus *Epalxella* truly nests within Odontostomatea since the partial 18S rRNA gene sequences of the species *E*. *exigua* and *E*. cf. *antiquorum* branch within the Odontostomatea subtree of the ciliate phylogenetic tree, unlike the previously sequence published under the name *Epalxella antiquorum* that clustered with plagiopylids (Stoeck et al. 2007). Since no morphological data were provided in the latter publication, we conclude that the sequence was erroneously attributed to *Epalxella antiquorum* and was actually from a misidentified ciliate. Moreover, the morphological characteristics of plagiopylids bear no resemblance to those of odontostomateans (Fernandes et al. 2018). Thus, the Odontostomatea is monophyletic.

Unfortunately, misidentifications still occur. For instance, a few single-cell metagenome sequences attributed to odontostomateans have recently been deposited in GenBank without associated morphologic data for the source organism (Zhang et al. 2024, 2025). After extraction of the 18S rRNA gene sequence from one of the metagenomes under the name *Mylestoma anatinum* (Penard, 1922) Kahl, 1932, we found that it, instead, belongs to *Saprodinium dentatum*. Similar cases have also been recently pointed out for 18S rRNA gene sequences from other groups (Pomahač et al. 2024; Rotterová et al. 2024)*.*

18S rRNA gene sequences of the odontostomateans are highly divergent with respect to sequences of other ciliates, which was a “feature” used for establishing the class Odontostomatea (Fernandes et al. 2018). Notably, sequences of particular odontostomatean species and genera are also highly divergent from each other, which was not noted before due to the scarcity of published sequences. This suggests that the 18S rRNA of odontostomateans has evolved relatively rapidly, usually preventing its amplification with “universal eukaryotic” primers. Additionally, previously published “universal eukaryotic” primers (Abraham et al. 2019) were mapped to the odontostomatean alignment, and only the primers F-566 and R-1200, flanking the region V4 and V5 (Hadziavdic et al. 2014), matched within the odontostomatean alignment, but only for members of the family Epalxellidae. As a result, many environmental studies based on the 18S rRNA gene amplicons overlook odontostomatean ciliates. However, the environmental odontostomatean sequences we identified from metatranscriptomic and metagenomic data suggest a greater, as yet unexplored, diversity.

Although Odontostomatea is monophyletic, its position within Ciliophora Doflein, 1901 remains unclear. In recent phylogenomic analysis, Odontostomatea nests within Armophorea (Zhang et al. 2025). This topology is inconsistent with any previous analyses based on single-gene phylogenies, where Odontostomatea is sister to Armophorea or Litostomatea (Fernandes et al. 2018; Méndez-Sánchez et al. 2023; Rotterová et al. 2020). Based on 18S rRNA gene analyses, our current phylogeny places Odontostomatea as sister to Armophorea or Litostomatea, although with low-to-moderate support. However, it is likely that Odontostomatea is closely related to the class Armophorea since several morphological similarities between the two taxa are found, e.g., the likely homologous perizonal stripe and epistomial fringe, the basis for their previous inclusion in the class Armophorea (Adl et al. 2019; Da Silva‐Neto et al. 2016; Fernandes et al. 2018; Jankowski 1964b).

### Environmental analysis reveals that Odontostomatea is unexpectedly diverse

The environmental odontostomatean 18S rRNA sequences retrieved from databases suggest that Odontostomatea are highly diverse, with clades representing potential new species, genera, or families, unlike the published metabarcoding environmental studies using standard eukaryotic barcoding primers (Burki et al. 2021; Karst et al. 2018). The discovered diversity covers a wide range of ecosystems from several localities around the globe (e.g., marine and freshwater sediments from wetlands, soil, estuaries, and creeks), recovering a broad range of diversity (Angle et al. 2017; Dang et al. 2022; Graf et al. 2021; Karst et al. 2018; Peterson et al. 2020; Woodcroft et al. 2018; Zhang et al. 2021) (Supplementary Tables S2, S3). Our analysis showed that the considerable diversity in the 18S rRNA gene sequences in odontostomateans is coupled with their vast morphological diversity (Kahl 1932a, b).

Since all of the 19 environmental sequences retrieved from marine samples (USA, Sweden, Baltic Sea) clustered with the marine *Mylestoma bipartitum* and *M*. *monodontum* into a poorly supported clade within Clade II, which is otherwise freshwater, we hypothesize that marine odontostomateans, particularly marine *Mylestoma*, have a freshwater ancestor.

We recognize that some environmental odontostomatean sequences may still contain artifacts or undetected chimeras, particularly when derived from assemblies of short reads in metagenomes or metatranscriptomes. These challenges can introduce noise into phylogenetic analyses, underscoring the need for caution when interpreting the true diversity of Odontostomatea. Furthermore, the two major clades of Odontostomatea and their respective subclades remained well or highly supported and congruent with the phylogeny when environmental sequences were excluded.

Odontostomatea appear to be a relatively recent taxon within Ciliophora (Zhang et al. 2025), begging the question of what caused their rapid diversification, which is more typical for endosymbiotic species (Vďačný 2018; Vďačný et al. 2019). One of the causes could be the acquisition of prokaryotic symbionts (Méndez-Sánchez et al. 2023; Paiva et al. 2017; Schrenk 1989; Schrenk and Bardele 1991), which are also common in anaerobic protists from anoxic sediments, similar to the case with their very diverse and closest armophorean relatives, the Metopida Jankowski, 1980 (Armophorea) (Esteban et al. 1995; Kahl 1932b; Pomahač et al. 2024; Rotterová et al. 2018; Schrecengost et al. 2024; Treitli et al. 2023).

### Redefinition of the families Discomoprhellidae, Epalxellidae, and Mylestomatidae and higher taxonomic ranks of Odontostomatea

The newly sequenced populations of various genera and species of odontostomateans provide the first internal phylogeny of the Odontostomatea, demonstrating congruency with its current classification: one order and three families (Corliss 1960; Fernandes et al. 2018; Kahl 1932b; Lynn 2008; Wetzel 1928). Our phylogenetic analysis strongly supports the monophyly of each of the taxa. However, odontostomatean sequences retrieved from environmental datasets, along with sequences of *Limnomylestoma* gen. nov., *Tostonella* gen. nov., and Odontostomatea sp. 2 and 3, suggest that additional families may exist within this class. Likewise, the genera *Pelodinium* and *Atopodinium*, which currently lack molecular data, may also represent distinct families. Since the genera *Limnomylestoma* and *Tostonella* differ morphologically, we hesitate to classify them into any known family at the present state of knowledge.

Morphologically, the family Epalxellidae was defined by the presence of ciliated kineties, one anterior and four on the posterior rear, except in *Pelodinium*, which has six posterior ones (Kahl 1932a). Through a detailed examination of various epalxellid genera and species, i.e., *Saprodinium*, *Epalxella*, and *Mircalla* gen. nov., we identify and define the so-called *Epalxella* pattern, characterized by the ciliary arrangement of the ciliated and non-ciliated segments of the somatic kineties. This pattern is proposed as a synapomorphy of the family. Although *Pelodinium reniforme* was previously classified within Epalxellidae (Jankowski 2007; Kahl 1932a, b; Lynn 2008), its infraciliature pattern does not conform to the *Epalxella* pattern (Fig. 2P) (Foissner et al. 1992; Kahl 1932a, b; Klein 1930; Wetzel 1928). Thus, we recommend its exclusion from Epalxellidae and consider it as *incertae sedis* in Odontostomatea until molecular data are available. Due to a lack of morphological details, it remains unclear whether odontostomatean sp. 1 (GROSERDU population), which clusters into Clade I, belongs to Epalxellidae. We, therefore, consider it as *incertae sedis*.

Based on morphology, Kahl (1932a) and Jankowski (1964a) suggested that Mylestomatidae and Discomorphellidae are more closely related to each other than to Epalxellidae, a relationship supported by our phylogenetic analysis. Additionally, *Tostonella* gen. nov. and *Limnomylestoma* gen. nov. also show a close phylogenetic relationship with these two families.

The strongly supported family Discomorphellidae currently contains a single genus, *Discomorphella*. The family has been well defined by its peculiar morphological features, e.g., fragmented epistomial fringe and a hoop-like frontal awning (Corliss 1960; Kahl 1932a; Lynn 2008). Given the current data, it is premature to include *Tostonella*, *Limnomylestoma,* and the odontostomatean species 2 and 3 within Discomorphellidae. Notably, Tuffrau (1992) described a structure as a paroral membrane in *Discomorphella pectinata* based on silver nitrate impregnation. However, based on electron microscopy images of *Discomorphella pedroeneasi*, *Saprodinium dentatum*, and *Mylestoma pusillum* (Paiva et al. 2017; Schrenk 1989; Schrenk and Bardele 1991) and our own observations (not shown), there is no evidence of a paroral membrane in odontostomateans. Tuffrau likely confused the edge of one of the oral cavities of the cytopharynx with a paroral membrane (see Fig. 3L in Méndez-Sánchez et al. 2023).

Kahl (1932a, b) established the family Mylestomatidae, grouping freshwater and marine species of *Mylestoma* and the freshwater genus *Atopodinium*, based on similarities in body shape and ciliature. Based on the similarities of *Limnomylestoma* and *Mylestoma*, which are unrelated genera, and the clustering of marine environmental sequences into a single clade, we are skeptical about the assignment of the freshwater *Mylestoma* species to Mylestomatidae. Thus, reexamining the remaining *Mylestoma* species is crucial to clarify the members of the family. The freshwater *Mylestoma* species may belong either to *Limnomylestoma* or another genus, since a detailed morphological characterization is missing for most of them. It also remains unclear whether *Atopodinium* or any freshwater *Mylestoma* species corresponds to any of the freshwater environmental sequences associated with the marine clade. A thorough revision of the family is therefore needed.

Furthermore, the robustly supported Clade I and II may represent distinct orders within Odontostomatea. The possible synapomorphies for Clade I include a posterior marginal concavity bordered by spines and somatic kineties consisting of up to three segments: an anterior ciliated segment, a middle non-ciliated segment, and a terminal spine segment (Jankowski 1964a; Kahl 1932a, b). Whereas Clade II includes two caudal cirri along the left posterior cell margin, a reduction of ciliature to non-ciliated somatic kineties only, and a smooth, posterior cell margin without a concavity. However, we refrain from establishing these potential orders until other genera, e.g., *Atopodinium*, *Pelodinium*, are morphologically and molecularly characterized.

### Establishment of new odontostomatean genera

Although the infraciliature of odontostomateans is reduced to several kineties, it remains complex due to the presence of ciliated and non-ciliated segments, which are only revealed with protargol or silver nitrate impregnation (Klein 1930; Schrenk and Bardele 1991; Tuffrau 1992). However, these techniques may miss finer structural details, leading to misinterpretations. Accurate identification also requires careful examination of the lateral aspects of the cortex, the structures of the ventral margin, and the number and arrangement of spines. Since these details are often not well seen in light microscopy, scanning electron microscopy is especially important for reliably distinguishing these structures and avoiding errors in species identification.

Odonstostomatea comprises nine genera, distinguishable primarily by in vivo features, except for very similar *Mylestoma* and *Limnomylestoma*. *Saprodinium* has right- and left-side posterior marginal spines with thorn-like ends. In contrast, in *Epalxella* and *Mircalla*, the posterior spines are less conspicuous and more or less blunt or rounded; they are more prominent on the left side and inwardly curving on the right side, creating a wavy posterior edge (Foissner and Berger 1996; Foissner et al. 1992; Kahl 1932a). Despite similar body shapes, *Mircalla* and *Epalxella* form distinct clades within Odontostomatea, which are not closely related. *Mircalla* uniquely bears a fang-like spine between the buccal cavity and frontal ridge, distinguishing it from *Epalxella* and other genera. This fang-like spine, together with its phylogenetic position, supports the establishment of *Mircalla* as a separate genus. Note that this spine differs from the lapel-like structure of *Saprodinium*, *Epalxella*, *Mylestoma*, and *Limnomylestoma*, which appears like a spine and was previously regarded as an oral one, but instead is a fold of the anterior plate supporting the buccal cavity (Méndez-Sánchez et al. 2023; Schrenk and Bardele 1991).

*Pelodinium* differs from all genera of Epalxellidae by having a rather small heart-shaped posterior concavity and possessing a unique infraciliature, i.e., the *Pelodinium* pattern (described above) (Foissner et al. 1992; Jankowski 1964a; Kahl 1932a, b). *Discomorphella* stands out from other odontostomateans due to its discoidal shape, prominent spines, and fragmented epistomial fringe on a hoop-like frontal awning (Lauterborn 1901; Paiva et al. 2017; Tuffrau 1992; Wetzel 1928).

Despite their phylogenetic distance and the rejection of constrained monophyly by the AU test (Supplementary Table S5), *Mylestoma* and *Limnomylestoma* are morphologically quite similar to each other and cannot be easily distinguished without silver impregnation (Kahl 1932a, b). While not all *Mylestoma* species have been redescribed with the current methodologies, and *Limnomylestoma* is monotypic, our examination of impregnated specimens shows subtle differences: (1) *Limnomylestoma* displays an amphikinetal caudal cirral arrangement, while *M. monodontum*, *M. anatinum*, and *M. bipartitum* show a semikinetal arrangement; (2) *Mylestoma* has a pronounced lapel-like structure, whereas *Limnomylestoma* exhibits a thinner, lamina-like structure (Méndez-Sánchez et al. 2023; Schrenk 1989). The interesting morphological convergences of *Mylestoma* and *Lymnomylestoma* warrant further investigation.

Kahl (1932a) suggested that the genus *Mylestoma* could possibly be further split into separate genera. Based on the morphology and phylogenetic position of *Tostonella uncinata* (original combination *Epalxis uncinatum* Penard, 1922), which does not cluster within Discomorphellidae, Mylestomatidae, or *Limnomylestoma*, we propose *Tostonella* as a new genus. It shares the discoidal body shape of *Discomorphella*, but differs from it by lacking the sharp spines, frontal awning, and fragmented epistomial fringe, characteristic features of *Discomorphella* (Lauterborn 1901; Lynn 2008; Paiva et al. 2017; Tuffrau 1992). *Tostonella* has an anterior hood-like structure originating from the dorsal keel, which covers the reduced epistomial fringe on the ventral surface, similar to *Mylestoma* and *Lymnomylestoma* (Kahl 1932a; Méndez-Sánchez et al. 2023). Unlike *Mylestoma*, the dorsal keel is conspicuous in *Tostonella*. Additionally, one pectinelle of the epistomial fringe is displaced posteriorly in *Tostonella*, a feature not observed in other odontostomatean genera. *Tostonella* has three posterior spines, one originating from the dorsal keel, and the other two from the ventral side of the cortex (Kahl 1932a; Penard 1922). We do not discard the possibility that *Atopodinium* is closely related to *Tostonella* due to its discoidal shape and multiple posterior spines. However, this assumption is challenged by the absence of a ventral kinety and the presence of a single caudal cirrus (vs. 2) in *Atopodinium* (Jankowski 1964a; Kahl 1932a, b). Based on these morphologic differences we establish the genus *Tostonella* for the populations BUL7A and CEDARKOR. A thorough examination of *Atopodinium* is needed to clarify its phylogenetic and morphological relationships with *Tostonella*.

The current phylogenetic tree suggests that additional genera will likely be recognized in the future, as Epalxellidae sp. 1, Odontostomatean sp. 1 (GROSERDU), and Odontostomatean sp. 2 and 3 may represent new taxonomic ranks.

### The odontostomatean species and their comparison with congeners

The genus *Saprodinium* comprises six valid species, with the type species, *Saprodinium dentatum*, being the most well known (Kahl 1932a, b; Méndez-Sánchez et al. 2023; Schrenk and Bardele 1991). Reexamination of the previously described VLKOV population (Méndez-Sánchez et al. 2023) revealed that *S*. *dentatum* has nine somatic kineties (excluding the preoral and ventral ones), composed of ciliated and non-ciliated segments arranged in the *Epalxella* ciliature pattern. Previous studies reported only eight somatic kineties (Jankowski 1964a; Klein 1930; Méndez-Sánchez et al. 2023; Schrenk and Bardele 1991); with one likely overlooked. While details on the infraciliature of other *Saprodinium* species are limited, *S*. *dentatum* is distinguished from them by having 94 ciliated dikinetids on average in the anterior segment of the SK8, and eight or nine caudal spines, including the ventrocaudal one (Kahl 1932a; Méndez-Sánchez et al. 2023; Schrenk and Bardele 1991).

*Saprodinium mimeticum* is distinguished from other *Saprodinium* species by its quadrangular body shape and long and conspicuous ventrocaudal spine. It is also smaller and has fewer dikinetids in the ventral kinety 1 and anterior ciliated segment of the SK8 compared to *S*. *dentatum* (6 vs. 21, and < 26 vs. > 80, respectively) (Foissner et al. 1992; Jankowski 1964a; Méndez-Sánchez et al. 2023; Schrenk and Bardele 1991). *S*. *mimeticum* differs from *S*. *spinigerum* Kahl, 1932 by lacking the long ventral spine, from *S*. *integrum* Kahl, 1928 by having shorter thorn-like posterior spines (vs. long), from *S*. *putrinum* by having longer posterior spines (vs. short), from *S*. *triangulum* Kahl, 1932 by its quadrangular body shape (vs. triangular), and from *S*. *halophilum* Kahl, 1932 by the presence of a frontal spine. Additionally, *S*. *halophilum* and *S*. *integrum* were found in marine and brackish environments, respectively, while the other species are freshwater (Dragesco and Dragesco-Kernéis 1986; Jankowski 1964a; Kahl 1928, 1932a, b; Kreutz 2024; Lauterborn 1901, 1908; Schrenk 1989). Kahl (1932a, b) established two forms, *S*. *mimeticum* f. *obliquum* and *S*. *mimeticum* f. *simplex*. Both forms require further investigation. *S*. *mimeticum* f. *simplex* might represent a different species, since its ventrocaudal spine is less conspicuous and prominent than in *Saprodinium mimeticum* (Kahl 1932a, b; Kreutz 2024).

The genus *Epalxella* comprises seven valid species: *E*. *mirabilis* (Roux, 1899) Corliss, 1960, *E*. *striata* (Kahl, 1926) Corliss, 1960, *E*. *elliptica* (Penard, 1922) Corliss, 1960, *E*. *exigua*, *E*. *antiquorum*, *E*. *bidens* (Kahl, 1932) Corliss, 1960, and *E*. *simplex* Jankowski, 1964. Here, we redescribe *E*. *exigua* using modern morphologic techniques and molecular data and provide the 18S rRNA sequence of *E*. cf. *antiquorum*. Both species form a moderately supported clade and share the characteristic of window-like cortical slits (Jankowski 1964a; Kahl 1932a, b). The population of *E*. *exigua* we studied matches the description by Kahl (1932a, b) and Penard (1922), particularly in the shape of the anterodorsal part of the cell, the absence of a frontal spine, and the presence of a single sharply demarcated window-like cortical slit on the left side. The only difference from the previous descriptions was the shorter length of the long cilia from the spine segment of SK9 in our population. *Epalxella exigua* has a blunt anteroventral margin and lacks the frontal spine, unlike *E*. *antiquorum*, *E*. *bidens*, and *E*. *mirabilis* (Jankowski 1964a; Kahl 1932a). *Epalxella elliptica*, *E. striata*, and *E*. *simplex* have a blunt frontal margin like *E*. *exigua*, but lack the window-like cortical slits. *Epalxella antiquorum* is a large species, possibly the largest in the genus or even in the class (Jankowski 1964a). The few cells observed in the sequenced populations resemble *E*. *antiquorum* in size and shape and displayed window-like cortical slits (four on the right side and 2 on the left), sufficiently distinctive features to identify it as *E*. *antiquorum*.

*Mircalla* gen. nov. contains two species, *M*. *triangula* comb. nov. (original combination *Epalxella triangula*) and *M*. *polidorii* sp. nov., both characterized by the presence of a ventral spine. *Mircalla polidorii* differs from *M*. *triangula* by the cell shape (ellipsoidal vs. triangular, respectively) and the presence of two preoral kineties (vs. one). Kahl (1932a, b) described *Epalxella triangula* Kahl, 1932 as having a more or less triangular body shape with a small frontal spine, distinguishing it from *Epalxella striata*, which has a more rounded anterodorsal side and lacks a frontal spine. Kahl (1932a, b) studied two populations, one small and one large. The African population resembles the smaller one described by Kahl (1932a, b), so we consider it conspecific with *E*. *triangula*. *Mircalla polidorii* and *Epaxella triangula* are closely related species and have similar morphology. Thus, we transfer the species *Epalxella triangula* to the newly established genus *Mircalla*. Until now, all the studied odontostomatean species bear two preoral kineties, except *Mircalla triangula,* which possesses a single one.

Morphologically, *Mylestoma bipartitum*, *M*. *monodontum*, *M*. *anatinum*, *M*. *spinigerum* Kahl, 1932, *M*. *pusillum* Kahl, 1932, and *Limnomylestoma shuriken* are similar (Gourret and Roeser 1886; Jankowski 1964a; Kahl 1932a; Méndez-Sánchez et al. 2023; Penard 1922; Schrenk 1989). Other species, such as *M*. *uncinatum*, *M*. *flagellatum* (Penard, 1922) Kahl, 1932, and *M*. *discoideum* (Penard, 1922) Kahl, 1932, differ from *Mylestoma anatinum*, *M*. *monodontum*, *M*. *bipartitum*, and *Limnomylestoma shuriken* by having a more discoidal and flattened body (Kahl 1932a, b; Kreutz 2024).

*Limnomylestoma shuriken*, *Mylestoma bipartitum*, and *M*. *monodontum* differ from *Mylestoma spinigerum* by lacking the long posterior spine on the right side and by having caudal cirri in the mid-portion of the margin, while in *M*. *spinigerum* are on the left side near the dorsal margin (Kahl 1932a).

*Mylestoma pusillum* is distinctive due to its small size (15–20 µm vs., other species, which are > 20 µm long), smooth posterior margin (no spines), and widely separated caudal cirri with long cilia (Kahl 1932a; Kreutz 2024; Schrenk 1989).

Based on Kahl’s descriptions (1928, 1932a, b), we identify the *Mylestoma* from KLAGOS as *Mylestoma bipartitum*, characterized by its large size, long epistomial fringe, two conspicuous posterior spines on the right side, and marine habitat. Although we could not determine the exact number of dikinetids in the ventral kinety, Kahl (1928, 1932a, b) depicted three. Details of this particular structure need to be confirmed. *Mylestoma bipartitum* is larger and has more pectinelles in the epistomial fringe and somatic kineties compared to *M*. *monodontum*, *M*. *anatinum*, and *Limnomylestoma shuriken* (< 40 µm, eight, and nine vs. > 30 µm, four, and < 8, respectively).

*Mylestoma anatinum* has a broad central concavity in the truncated posterior region and eight somatic kineties, in contrast to *M*. *monodontum* and *Limnomylestoma shuriken*, which both lack a posterior marginal concavity and each of which has seven somatic kineties (Kreutz 2024; Méndez-Sánchez et al. 2023; Schrenk 1989). The caudal cirri are semikinetal in *M*. *anatinum* and *M*. *monodontum* but amphikinetal in *L*. *shuriken* (Schrenk 1989). Additionally, *L*. *shuriken* has six dikinetids in the ventral kinety, whereas *M*. *monodontum* has four (Méndez-Sánchez et al. 2023). Additionally, *M*. *anatinum* and *Limnomylestoma shuriken* are freshwater species, while *M*. *monodontum* and *M*. *bipartitum* are marine.

The odontostomatean from the BUL7A population resembles *Mylestoma uncinatum* (Pernard, 1922) Kahl, 1932 (original combination *Epalxis uncinatum*). Despite lacking details for the infraciliature of *M*. *uncinatum*, the body shape, size, and spine number and arrangement support conspecificity with BUL7A (Kahl 1932a). Based on its phylogenetic position, we transfer *Mylestoma uncinatum* to the new genus *Tostonella*. *Tostonella uncinata* differs from *Mylestoma flagellatum* by lacking a ventral spine, having five pectinelles in the epistomial fringe (vs. ca. nine), and featuring a displaced pectinelle (Kahl 1932a). Accordingly, a population identified by Kreutz (2024) as *Mylestoma flagellatum* is more likely *Tostonella uncinata*.

*Discomorphella* contains three species: *D*. *pectinata*, *D*. *lauterborni* (Wetzel, 1928) Corliss, 1960, and *D*. *pedroeneasi*. *Discomorphella lauterborni* differs from the other two species by having only two spines (one frontal and one posterior) and lacking the lateral spine present in *D*. *pectinata* and *D*. *pedroeneasi* (Levander 1894; Paiva et al. 2017; Wetzel 1928). *Discomorphella pedroeneasi* has a conspicuous fourth spine associated with the left fragment of the epistomial fringe, which is reduced or absent in *D*. *pectinata*. Additionally, *D*. *pectinata* has more pectinelles in the epistomial fringe (> 21 in the dextro-frontal fragment and nine in the left fragment vs. 14 and 6 in *D. pedroeneasi*, respectively). *Discomorphella pedroeneasi* also has three precaudal dikinetids that are absent in *D*. *pectinata*.

*Discomorphella pectinata* is divided into two subspecies, distinguished by the number of short spines on the left fragment of the epsitomial fringe: one in *D*. *pectinata pectinata* and two in *D*. *pectinata bidenticulata* (Kahl, 1932) Corliss, 1960 (Kahl 1932a, b; Lauterborn 1908; Paiva et al. 2017). Paiva et al. (2017) suggested that populations lacking spines on the left fragment, as noted by Jankowski (1964a) and Tuffrau (1992), might represent a different subspecies. We sequenced the 18S rRNA gene of specimens with and without a spine from two *Discomorphella* populations. The sequences showed no genetic divergence (100% identity), indicating no subspecies differentiation. Thus, the presence or absence of a spine on the left fragment is not a reliable character for species delimitation. Further study is needed to clarify the status of *D*. *pectinata bidenticulata*.

## Conclusions

This study provides the first large-scale survey of class Odontostomatea, characterizing several species and genera and redefining the three known families from a morphological and molecular perspective. This group has been neglected for several decades, and some members were incorrectly classified. Due to the highly variable 18S rRNA gene of odontostomateans, specific primers were designed for this study, which will help to refine the phylogeny of the group in future studies. This study reveals the monophyly of Odontostomatea and clarifies the morphologic identification and molecular characterization of the previously problematic genus *Epalxella*. We also identify two main clades with morphological trends that could be used to create taxonomic orders. Additionally, new genera and species are proposed. Based on the environmental analyses, odontostomatean diversity appears to be far greater than previously supposed, with as yet undiscovered families and genera waiting to be described. This taxonomic revision will benefit future taxonomists when revisiting previously described or new species. Furthermore, the identity of prokaryotic symbionts, such as methanogenic archaea, in odontostomatean ciliates remains unknown. Since Odontostomatea seems to have emerged as a relatively recent taxon within Ciliophora, it would be interesting to study the timing of acquisition and the host specificity of these prokaryotic symbionts, and compare these with other closely related groups, e.g., Metopida. The true phylogenetic position of Odonstostomatea within Ciliophora needs to be elucidated with multiple-taxon “omic” analyses.

### Taxonomic summary

**ZooBank registration of this work:** urn:lsid:zoobank.org:pub:686C9F7C-68C1-4596-A9E4-50B8F6DBFEB2

### Class Odontostomatea Fernandes et al., 2018

**Improved diagnosis:** Size about 20–90 µm, anaerobic ciliates; body compressed; thick and rigid armor-like cortex with spines; alveoli reduced or absent; basic ciliary unit dikinetid, kinetodesmal fibers absent; somatic cilia usually arising from cortical sockets; somatic infraciliature reduced, somatic kineties composed of ciliated and/or non-ciliated segments; subsurface basal bodies present; oral apparatus comprising at least two compartments; typically < 10 adoral membranelles exhibiting inverted orientation; paroral membrane absent.

### **Order Odontostomatida Sawaya, 1940**

Characteristics of the class. Three families: Epalxellidae Corliss, 1960; Discomorphellidae Corliss, 1960; Mylestomatidae Kahl in Doflein & Reichenow, 1929.

### **Family Epalxellidae Corliss, 1960**

**Improved diagnosis:** Posterior marginal concavity bordered by posterior spines; dorsal keel; lapel-like structure; inverse kinety two preoral kineties; somatic kineties arranged in *Epalxella* pattern.

**Type and included genera:** *Epalxella* Corliss, 1960 (type genus); *Saprodinium* Lauterborn, 1908; and *Mircalla* gen. nov.

### **Genus *Saprodinium* Lauterborn, 1908**

**Improved diagnosis:** Posterior spines with conspicuous thorn-like ends; cortex without window-like cortical slits.

**Species included:** *S*. *dentatum* (Lauternorn, 1901) Lauterborn, 1908 (type species); *S*. *mimeticum* (Penard, 1922) Kahl, 1932: *S. integrum* Kahl, 1928; *S*. *putrinum* Lackey, 1925; *S. triangulum* Kahl, 1932; *S*. *spinigerum* Kahl, 1932; and *S. halophilum* Kahl, 1932.

***Saprodinium dentatum*** (Lauternorn, 1901) Lauterborn, 1908

**Nomenclatural acts:** *Discomorpha dentata* Lauternorn, 1901 (original combination)

**Improved diagnosis:** Size 50–80 µm; shape semicircular; eight or nine posterior spines, including ventrocaudal spine; > 80 ciliated dikinetids in the anterior segment of somatic kinety 8; > 10 dikinetids in spine segment of somatic kineties.

**GenBank sequence:** Five 18S rRNA gene sequences were obtained, one per studied population, VLKOV (PQ699762), GROSERDU (PQ699743), MOKOTR (PQ699758), LERMA3 (PQ699751), and SLATINA (PQ699761).

**Voucher material:** Four voucher slides, two from VLKOV and two from LERMA3 populations, containing protargol-impregnated specimens, and two aluminum stubs with Pt/Pd-coated specimens (MOKOTR population) are deposited in the National Museum of the Czech Republic, Prague, Czech Republic (catalog numbers P6E 5604–P6E 5607 for slides and P6E 5643 and P6E 5647 for SEM stubs).

***Saprodinium mimeticum*** **(Penard, 1922) Kahl, 1932**

**Nomenclatural acts and synonyms:** *Epalxis mimetica* Penard, 1922 (original combination); *Saprodinium tortum* Kahl, 1926.

**Improved diagnosis:** Size 40–60 µm; shape quadrangular; seven posterior spines, including ventrocaudal spine; ventrocaudal spine long and prominent; 15–32 dikinetids in the anterior segment of somatic kinety 8; < 10 dikinetids in spine segment of somatic kineties.

**Voucher material:** Seven voucher slides, four from IS7 and three from BOPAT populations, containing protargol-impregnated specimens, and four aluminum stubs with Pt/Pd-coated specimens, three from BYMOKOT and one from IS7 populations, are deposited in the National Museum of the Czech Republic, Prague, Czech Republic (catalog numbers P6E 5608–P6E 5614 for the slides and P6E 5640–5642 and P6E 5645 for SEM stubs).

**GenBank sequence:** Five partial 18S rRNA gene sequences were obtained: three from IS7 (PQ699744–PQ699746), one from BYMOKOT (PQ699735), and one from BOPAT (PQ699730) populations.

**Remarks**: The forms *Saprodinium mimeticum* f. *obliquum* Kahl, 1932; and *Saprodinium mimeticum* f. *simplex* Kahl, 1932, require further examination.

### **Genus *Epalxella* Corliss, 1960**

**Improved diagnosis:** Posterior right margin wavy, with spines folded inwards, and left margin with three spines; posterior spines blunt, without thorn-like ends.

**Species included:** *E*. *mirabilis* (Roux, 1899) Corliss, 1960 (type species); *E*. *antiquorum* (Penard, 1922) Corliss, 1960; *E*. *exigua* (Penard, 1922) Corliss, 1960; *E*. *striata* (Kahl, 1926) Corliss, 1960; *E*. *elliptica* (Penard, 1922) Corliss, 1960; *E*. *bidens* (Kahl, 1932) Corliss, 1960; and *E*. *simplex* Jankowski, 1964.

**Remarks:** Based on current literature and data, only *Epalxella exigua* and *E*. *antiquorum* possess window-like cortical slits. Molecular data for the remaining species could potentially clarify the taxonomic significance of this character.

***Epalxella exigua*** **(Penard, 1922) Corliss, 1960**

**Nomenclatural acts:** *Epalxis exigua* Penard, 1922 (original combination)

**Improved diagnosis:** Size 30–37 × 23–29 µm; shape ellipsoidal; frontal spine absent; ventro-frontal edge blunt; a single and conspicuous window-like cortical slit on the left anterior surface; one ventral kinety with six dikinetids in two groups; two supplementary ventral dikinetids.

**Voucher material:** One voucher slide, with protargol-impregnated specimens, is deposited in the National Museum of the Czech Republic, Prague, Czech Republic (catalog number P6E 5619).

**GenBank sequence:** Two partial 18S rRNA gene sequences were obtained, one per studied population, LAUFENJ (PQ699750) and ZLATOVLASKA (PQ699764).

**Genus** ***Mircalla*** **gen. nov.**

**Zoobank registration:** urn:lsid:zoobank.org:act:7AD9396F-0ADD-488B-B711-430B113D0100

**Diagnosis:** A conspicuous ventral spine above the equator on the left ventral margin; frontal spine absent or very reduced; posterior spines without thorn-like ends; without window-like cortical slits.

**Included species:** *Mircalla polidorii* sp. nov. (type species); and *Mircalla triangula* (Kahl, 1932) comb. nov.

**Etymology:** Derived from one of the names of the lady vampire from the Gothic novella Carmilla by Sheridan Le Fanu (1872), referring to the presence of a fang-like ventral spine; feminine gender.

**Remarks:** Based on current knowledge, we assigned *Mircalla triangula* comb. nov. and *Mircalla polidorii* sp. nov. to the same genus due to their molecular phylogenetic position. *M*. *triangula* requires further examination, especially using scanning electron microscopy, to confirm the presence of the ventral spine. However, the possibility that *Mircalla triangula* represents a different genus cannot be excluded.

***Mircalla polidorii*** **sp. nov.**

**Zoobank registration:** urn:lsid:zoobank.org:act:496FD896-738E-4FC8-B2C7-B32AD33EEA3B

**Diagnosis:** Size 20–33 µm; shape ellipsoidal; frontal spine absent; ventro-frontal edge blunt; epistomial fringe with 11–15 pectinelles; two preoral kineties with 8–10 and 4–5 dikinetids, respectively.

**Type locality:** Small permanent pond adjacent to the Vltava river in Prague, Czech Republic, 49°59′24.9″ N 14°24′04.1″ E, freshwater.

**Type material:**Two slides, containing the protargol-impregnated holotype (marked with a black ink circle) and several paratype specimens, and one aluminum stub with Pt/Pd-coated specimens are deposited in the National Museum of the Czech Republic, Prague, Czech Republic (catalog number P6E 5615 and P6E 5616 for slides and P6E 5644 for the SEM stub).

**GenBank sequence:** One partial 18S rRNA gene sequence with accession number PQ699756.

**Etymology**: Eponym for the author John William Polidori, who wrote the first modern vampire story, “The Vampire,” referring to the presence of a fang-like ventral spine.

***Mircalla triangula*** **(Kahl, 1932) comb. nov.**

**Nomenclatural acts:** *Epalxis triangula* Kahl, 1932 (original combination).

**Improved diagnosis:** Size 32–37 µm; shape triangular; frontal spine highly reduced; epistomial fringe with 6–8 pectinelles; a single preoral kinety with 10–15 dikinetids; 3–4 supplementary dikinetids.

**Type locality:** A freshwater pond in Bayanga, Central African Republic, 2°50′32″ N, 16°27′56″ E.

**Voucher material:** Two voucher slides, with protargol-impregnated specimens, are deposited in the National Museum of the Czech Republic, Prague, Czech Republic (catalog number P6E 5617 and P6E 5618).

**GenBank sequence:** One partial 18S rRNA gene sequence with accession number PQ699738.

### **Family Mylestomatidae Kahl in Doflein & Reichenow, 1929**

**Improved diagnosis:** Small-to-medium odontostomatea; epistomial fringe ventrally oriented; 7–9 non-ciliated kineties; one or two caudal cirri.

**Type and included genera:** *Mylestoma* Kahl, 1928 (type genus); and *Atopodinium* Kahl, 1932

### **Genus *Mylestoma* Kahl, 1928**

**Improved diagnosis:** Shape semicircular or circular; frontoventral margin ends as hood-like structure; posterior spines poorly developed; two preoral kineties and one ventral kinety; inverse kinety; 7–9 non-ciliated kineties; two caudal cirri arranged in a semikinetal pattern; lapel-like structure.

**Species included:** *M*. *bipartitum* (Gourret & Roeser, 1886) Kahl, 1928 (type species); *M*. *anatinum* (Penard, 1922) Kahl, 1932; *M*. *discoideum* (Penard, 1922) Kahl, 1932; *M*. *flagellatum* (Penard, 1922) Kahl, 1932; *M*. *monodontum* sp. nov;* M*. *pusillum* Kahl, 1932; and *M*. *spinigerum* Kahl, 1932.

***Mylestoma bipartitum*** **(Gourret & Roeser, 1886) Kahl, 1928**

**Nomenclatural acts:** *Aspidisca bipartita* Gourret & Roeser, 1886 (original combination).

**Improved diagnosis:** Size 33–42 µm; right side vaulted; posterior cavity present; 7–9 epistomial pectinelles; several spines on the ventral edge and four on the posterior right edge; preoral kinety 1 and 2 with 8–11 and 6–9 dikinetids, respectively; nine non-ciliated somatic kineties; caudal cirri with 8–10 kinetids; brackish habitat.

**Voucher material:** Four voucher slides, containing protargol-impregnated specimens, are deposited in the National Museum of the Czech Republic, Prague, Czech Republic (catalog number P6E 5621–P6E 5624).

**GenBank sequence:** One partial 18S rRNA gene sequence with accession number PQ699747.

***Mylestoma monodontum*** **sp. nov.**

**Zoobank registration:** urn:lsid:zoobank.org:act:5147563F-23F4-447E-BDE5-CBB86B9FE2BC

**Diagnosis:** Size 18–25 µm; posterior cavity absent; four epistomial pectinelles; one spine at the right edge of the ventral fringe, one inconspicuous spine on the posterior right side; preoral kinety 1 and 2 with 5–6 and 3 dikinetids, respectively; seven non-ciliated somatic kineties; brackish habitat.

**Type locality:** Fresh River, Falmouth, Massachusetts, USA, 41°32′30.804″ N, 70°37′19.92″ W, brackish.

**Type material:** One slide containing the protargol-impregnated holotype specimen (FRESHO population) marked with a black ink circle, and three slides with protargol-impregnated paratypes (FARO population), are deposited in the National Museum of the Czech Republic, Prague, Czech Republic (catalog numbers P6E 5306 and P6E 5307, P63 5625, and P63 5626).

**GenBank sequence:** Two partial 18S rRNA gene sequences from the FARO population were obtained with accession numbers PQ699739 and PQ699740.

**Etymology**: Adjective, compound of the Greek prefix “mono-” (single) and “odontos” (tooth), referring to the single spine on the ventral ridge.

### **Family Discomorphellidae Corliss, 1960**

**Improved diagnosis:** Shape discoidal; dorsal keel flattened; one long, sharp frontal spine; one or two sharp spines on the right lateral side; epistomial fringe segmented in dextro-frontal and left fragments on ventral awning; pectinelles armored with auricules; papillary dikinetids present; one preoral kinety armored with auricules; two endoral kineties; two ventral kineties with supplementary ventral dikinetids; non-ciliated somatic kineties arranged in *Discomorphella* pattern; two caudal cirri.

**Type and only included genus:** *Discomorphella* Corliss, 1960

### **Genus *Discomorphella* Corliss, 1960**

**Nomenclatural acts:** Replacement name for preoccupied *Discomorpha* Corliss, 1960

**Improved diagnosis:** Characteristics like in the family. See Paiva et al. 2017.

**Included species:** *D*. *pectinata* (Levander, 1894) Corliss, 1960 (type species); *D*. *pedroeneasi* Paiva et al., 2017; and *D*. *lauterborni* (Wetzel, 1928) Corliss, 1960.

***Discomorphella pectinata*** (Levander, 1894) Corliss, 1960

**Nomenclatural acts:** *Discomorphea pectinata* Levander, 1894 (original combination).

**Improved diagnosis:** Size 60–85 µm > 17 pectinelles in dextro-frontal and 8–10 in left fragment, respectively; left fragment with or without associated spine; posterior fringe ratio > 1; without precaudal dikinetids.

**Voucher material:** Seven slides, two from BOTAN, three from MOKOT, and two from LAPLICE populations, containing protargol-impregnated specimens, and two aluminum stubs with Pt/Pd-coated specimens, one from BOTAN and one from MOKOT populations, are deposited in the National Museum of the Czech Republic, Prague, Czech Republic (catalog numbers P63 5633–P63 5639 for the slides and P6E 5643 and P6E 5647 for the SEM stubs).

**GenBank sequence:** Six sequences were obtained: two from LAPLICE (PQ699748-9), and three from BOTAN (PQ699731–PQ699733); one consensus sequence (PQ699753) was obtained from three sequences derived from cloning from MOKOT.

**Remarks:** Based on morphology and molecular evidence, we consider *Discomorphella pectinata pectinata* to be conspecific with *Discomorphella pectinata*. Therefore, we synonymize *D. pectinata pectinata* with *D. pectinata*. The validity of the subspecies *Discomorphella pectinata bidenticulata* (Kahl, 1932) Corliss, 1960 requires further examination.

### ***Incertae sedis* within Odontostomatida:**

*Pelodinium reniforme* Lauterborn, 1908; *Tostonella* gen. nov.; *Limnomylestoma* gen. nov.

### **Genus *Pelodinium* Lauterborn, 1908**

**Improved diagnosis:** Ellipsoidal shape, dorsal keel without frontal spine; posterior marginal heart-shape concavity present, bordered by few blunt spines; inverse kinety; two preoral kineties; two ventral kineties; 10 somatic kineties arranged in *Pelodinium* pattern, with long spine segments.

**Type and only included species:** *Pelodinium reniforme* Lauterborn, 1908

***Pelodinium reniforme*** **Lauterborn, 1908**

**Synonyms:** *Pelodinium rotundum* Kahl, 1926; *Epalxis penardi* Wetzel, 1928

**Diagnosis:** Size 20–50 µm; ellipsoidal shape; ventral margin spine present; posterior heart-shaped concavity surrounded by about six blunt spines; inverse kinety with two dikinetids; without caudal cirri.

### **Genus *Tostonella* gen. nov.**

**Zoobank registration:** urn:lsid:zoobank.org:act:2A5185E1-12EE-4371-A1EA-66136A2683C5

**Diagnosis:** Discoidal shape, posterior spines, hood-like structure; epistomial fringe reduced to five ventrally located pectinelles, left-most pectinelle displaced posteriorly; two preoral kineties, one ventral kinety, non-ciliated kineties present; two caudal cirri with long cilia.

**Type and only included species:** *Tostonella uncinata* (Penard, 1922) comb. nov.

**Etymology:** Derived from the Mexican slang word “tostón” (a low-value coin), referring to the coin in shape and swimming pattern which mimics a flipping coin; feminine gender.

***Tostonella uncinata*** **(Penard, 1922) comb. nov.**

**Original combination:** *Epalxis uncinatum* Penard, 1922

**Diagnosis:** Size 28–34 µm; three posterior spines; preoral kinety 1, 2, and ventral kinety with 5–8, 6–8, and 9–10 dikinetids, respectively; one and three non-ciliated kineties on the left and right side, respectively; caudal cirri located on posterior spine I, each with seven kinetids; freshwater species.

**Type locality:** A freshwater pond in the Arkutino Reserve, Primorsko, Bulgaria. 42°19′53.0″ N 27°43′33.3″ E.

**Voucher material:** Two slides containing protargol-impregnated voucher specimens and one aluminum stub with Pt/Pd-coated specimens from the BUL7A population, are deposited in the National Museum of the Czech Republic, Prague, Czech Republic (catalog numbers P6E 5627 and P6E 5628 for the slides and P6E 5642 for the SEM stub).

**GenBank sequence:** Two 18S rRNA gene sequences were obtained, one from BUL7A (PQ699734) and one from CEDARKOR (PQ699737).

### **Genus *Limnomylestoma* gen. nov.**

**Zoobank registration:** urn:lsid:zoobank.org:act:6C731B3B-67A3-4A22-B211-1E1FF21A5BCD

**Diagnosis:** Shape semicircular; hood-like structure; posterior concavity absent; posterior spines conspicuous; < 5 pectinelles; two caudal cirri arranged in amphikinetal pattern; lapel-like structure lamina-like, i.e., without fold; inverse kinety; freshwater habitat. Phylogenetically, more closely related to *Tostonella* and *Discomorphella* than Mylestomatidae*.*

**Type and only included species:** *Limnomylestoma shuriken* sp. nov.

**Etymology:** A compound of Greek prefix “limno-” (lake, pond), referring to the freshwater nature of the genus, and “mylestoma,” referring to the morphological similarities to the genus *Mylestoma*; neuter gender.

***Limnomylestoma shuriken*** **sp. nov.**

**Zoobank registration:** urn:lsid:zoobank.org:act:1810DD96-B1E5-4140-95E6-B75FAB6431DF

**Diagnosis:** Size 24–33 × 18–26 µm; with two or three prominent spines: one short ventral spine, one posterior spine, and one fin-like structure; 3–4 epistomial pectinelles.

**Type locality:** Slatina pond, Dubeč, Czech Republic, 50°04′13.2″ N 14°34′12.5″ E, freshwater.

**Type material:** Four slides, containing the protargol-impregnated holotype (marked with a black ink circle) and several paratype specimens, and one aluminum stub with Pt/Pd-coated specimens are deposited in the National Museum of the Czech Republic, Prague, Czech Republic (catalog numbers P6E 5629–P6E 5632 for the slides and P6E 5650 for the SEM stub).

**GenBank sequence:** Two partial 18S rRNA gene sequences with accession numbers PQ699759-60.

**Etymology:** Derived from the Japanese word “shuriken,” referring to the cell shape that resembles the star-like shape of a shuriken, a traditional throwing star weapon; noun in apposition.

## Glossary

Given the complexity of the ciliature and the general cell shape of odontostomateans, we explain here specific terms used in this paper. These terms are either adapted from previous works (Foissner et al. 1992; Klein 1930; Paiva et al. 2017; Schrenk and Bardele 1991; Tuffrau 1992; Wetzel 1928) or, where indicated, newly proposed in this work.

***Auricules*:** Conspicuous projections of the borders of *pectinelles* (Fig. 2O) (Paiva et al. 2017).

***Ciliary sockets*:** Small but conspicuous pits, delimited by the thick epiplasm layer, from which the cilia emerge. Also referred to as odontostomatid ciliary sockets (Paiva et al. 2017).

***Cortical pits*:** Small holes in the cortex, likely reduced *ciliary sockets*, immediately underneath the cell surface dilate into large parasomal sacs. Cortical pits are part of the *non-ciliated segments of kineties* (Schrenk and Bardele 1991).

***Cortical slit*:** Sharply demarcated, deep, more or less oval-shaped cortical depression occurring on the right and left sides of some Epalxellidae. Vary in number. Referred to by Kahl (1932a, b) as “Fensterbildungen” (window formations).

***Dorsal keel*:** Flatttened anterodorsal margin of the cortex. It is a dorsal continuation of the frontal spine or the hood-like structure (Fig. 2A–E) (Schrenk and Bardele 1991).

***Epistomial fringe (also frontal band)*:** A conspicuous row of *pectinelles* running perpendicularly to the main body axis, starting from the left side of the cell, embracing the *ventral ridge*, and ending on the right side of the cell in a staggered manner. The most anterior frontal ciliary row is the longest, while the most posterior is the shortest. The epistomial fringe can be long or short, continuous or fragmented into two sections: Dextro-frontal fragment (F1) forming a frontal awning and left fragment (F2); fragments linked by a conspicuous *epistomial fringe spacer* (Paiva et al. 2017; Schrenk and Bardele 1991; Tuffrau 1992).

***Epistomial fringe spacer*:** An elevation of the body surface separating the two fragments of the *epistomial fringe* (Paiva et al. 2017).

***Frontal awning*:** A protrusion of the region containing the epistomial fringe, as opposed to what is present in *Saprodinium*, in which the fringe is etched in the cell surface, forming a frontal band (Paiva et al. 2017).

***Frontal band:*** see epistomial fringe.

***Inverse kinety*:** Two or three dikinetids emerging from a cortical pit at the base of the *lapel-like structure*. The inversion of the kinety is known from TEM observations (Schrenk and Bardele 1991).

***Lapel-like structure (new term)*:** The outer left plate of the buccal cavity anteriorly folds outwards forming a lapel-like structure, which in vivo resembles a spine and previously was referred as an oral spine (Méndez-Sánchez et al. 2023; Schrenk and Bardele 1991), at the base of the fold, an *inverse kinety* rises from a cortical pit (Fig. 2P).

***Papillary dikinetids*:** Ciliated dikinetids at the end of the dextro-frontal fragment of the *epistomial fringe* in which cilia arise from slightly elevated *ciliary sockets*, forming papillae on the cell surface (Paiva et al. 2017).

***Pectinelle*:** Longitudinal row of one-to-six, usually five, *ciliary sockets* from which the cilia of the *epistomial fringe* arise. The pectinelles are separated by notched interkinetal ridges (Fig. 2N) or *auricules*. Pectinelles might be homologous to the false kineties in metopids (Armophorea) (Lynn 2008).

***Subsurface basal bodies (also barren dikinetids)*:** Non-ciliated basal bodies located below the epiplasm layer and interleaved with parasomal sacs. Subsurface basal bodies are the components of the non-ciliated segments of somatic kineties (Paiva et al. 2017). Note that this term was mistakenly used as a synonym for *cortical pits*.

***Ventral flap*:** Expansion of the left wall at the margin of ciliary sockets of the posterior part of ventral kineties (Paiva et al. 2017).

***Ventral ridge*:** Bulge of the cortex that supports the *epistomial fringe*, located above the cell equator on the ventral side (Schrenk and Bardele 1991).

***Ventrocaudal spine (also spine 1):*** An extension of the cortex forming the most ventral spine on the posterior right side of the cell, which bears the ventral kineties (Schrenk and Bardele 1991).

## Supplementary Information

Below is the link to the electronic supplementary material.

Supplementary file1 (PDF 3808 KB)

Supplementary file2 (DOCX 103 KB)

Supplementary file3 (XLSX 17 KB)

Supplementary file4 (XLSX 166 KB)

Supplementary file5 (XLS 52 KB)

Supplementary file6 (DOCX 20 KB)

## Data Availability

18S rRNA gene sequences obtained in this study have been submitted to GenBank (https://www.ncbi.nlm.nih.gov/genbank/). Type and voucher materials are available upon request from the National Museum of the Czech Republic, Prague, Czech Republic (Václavské nám. 68, 110 00 Nové Město, Czechia, Tel.: +420 224 497 111).
