# Supplementary material for: An extensive morphological and molecular characterization of the neglected class Odontostomatea (Ciliophora)
Source: Mar Life Sci Technol. 2026 Mar 12;8(2):289–323. doi: 10.1007/s42995-026-00352-x (PMC13198586; doi:10.1007/s42995-026-00352-x)
Supplement: Supplementary file 2 — Supplementary file2 (DOCX 103 KB) [file 42995_2026_352_MOESM2_ESM.docx]

**Table S1.** Oligonucleotide primers used to amplify the 18S rRNA gene sequence of odontostomatean ciliates.

| **Primer** | **Sequence 5′-3′** | **Paired with primer/ Annealing temperature (°C)** | **Potential target** |
| --- | --- | --- | --- |
| **OdoF2** | GTWGKGYTGTATTTATTAGATACT | Medlin Bc (52) | *Saprodinium dentatum* (SLATINA, VLKOV2, LERMA3)  Epalxellidae (MOKOT, CANSYK, M1LOT)  *Mircalla polidorii* (MOKOTP1)  *Mircalla triangula* (EPOPOLO)  *Epalxella exigua* (LAUFENJ)  *Epalxella* cf. *antiquorum* (ZLAKOR2A, MOKOTNOV)  Odontostomatea (GROSERDU)  *Mylestoma monodontum* (FARO) |
|  |  | OdoR4 (52) | *Epalxella exigua* (ZLATOVLASKA) |
|  |  | OdoR5 (52) | *Saprodinium dentatum* (GROSERDU, MOKOTR)  Odontostomatea (GROSERDU) |
| **OdoF3** | GTGCTGTATTTATTAGATACTAACCT | OdoR5 (52) | *Saprodinium mimeticum* (BOPAT) |
|  |  | Medlin Bc (52) | *Saprodinium mimeticum* (IS7) |
| **DisF1** | GGTATGATAYCGAAAATCGAGC | Medlin Bc (52) | Odontostomatea (3S6A*)*  *Tostonella uncinata* (BUL7A)  *Mylestoma bipartitum* (KLAGOS) |
|  |  | O1740R (52) | *Limnomylestoma shuriken* (SLATINA) |
|  |  | Dis1573R (52) | *Discomorphella pectinata* (BOTAN, LAPLICE) |
| **Dis425F** | CAGGGARAWAGTGAAAARAC | Dis1573R (52) | *Discomorphella pectinata* (BOTAN) |
| **DIs81F** | AGACTGYGGATGGTTTAT | Dis1573R (52) | *Discomorphella pectinata* (BOTAN, LAPLICE) |
| **OUF** | ATCAGTTATAGTTTATATGATAGATT | OUR (45) | *Saprodinium mimeticum* (BYMOKOT)  *Epalxella* cf. *antiquorum* (MOKOTP2) |
| **OdoF3m** | GGTTGTATTTATTAGATACTAACCG |  |  |
| **Dis1573R** | GGCGGTGAGTACAAAAGA |  |  |
| **Odo1687R** | ACCTTGTTACAACTTYTNM |  |  |
| **Medlin Bc** | TGATCCTTCTGCAGGTTCACCT |  |  |
| **OdoR5** | GAAACCTTGTTACAACTTCTRC |  |  |
| **OdoR1** | CGATGGGSGGTGTCTACAAAKRRT | OdoF2 (52) | Works |
| **OdoR2** | TGGGSGGTGTCTACAAAKRRT | OdoF2 (52) | Works |
| **OdoR3** | CTATTCCCATCACGATGCATA |  |  |
| **OdoR4** | GATCTATTCCCATCACGATGCATA |  |  |
| **O1740R** | CTACWRAWACCTTGTTACAACT |  |  |
| **O200F** | TATTAGATACTAACCKWTGGTGA |  |  |
| **OdoF1** | GKGYTGTATTTATTAGATACT |  |  |
| **OUR** | AWACCTTGTTACAACTTYT |  |  |
| **Sap836F** | GGGCATTAGTATTTAATTATCAGAGG | Sequencing |  |
| **Sap836R** | CCTCTGATAATTAAATACTAATGCCC | Sequencing |  |
| **Dis825F** | GGARCRRTTGGRGRTGATAG | Sequencing |  |
| **Dis825R** | CTATCAYCYCCAAYYGYTCC | Sequencing |  |
| **OintR1162** | AGTTTTCSCGWGTTGAGTC | Sequencing |  |

**Table S5.** Topological AU tests of alternative hypotheses.

| **Hypothesis tested** | **logL** | | **p-AU** |
| --- | --- | --- | --- |
| H1: Unconstrained | | -46512.52 | 1.000 |
| H2: *Mylestoma* + *Limnomylestoma* | | -46795.80 | 6e-63 |
| H3: *Mylestoma* + *Limnomylestoma* + sequence AY821980 | | -46674.90 | 1e-08 |

logL: log-likelihood; p-AU: p-value of the approximately unbiased test.

**Table S6.** Morphometric data on *Saprodinium mimeticum* IS7 and BOPAT populations.

| **Character** | **Strain** | **Mean** | **M** | **SD** | **CV** | **Min** | **Max** | **N** |
| --- | --- | --- | --- | --- | --- | --- | --- | --- |
| Body length, in vivo | IS7 | 52,5 | 52,5 | 2,2 | 4,3 | 48,9 | 55,5 | 12 |
|  | BOPAT | 54,9 | 54,9 | 2,5 | 4,6 | 50,1 | 59,9 | 12 |
| Body width, in vivo | IS7 | 38,7 | 39,5 | 3,6 | 9,3 | 32,3 | 42,9 | 12 |
|  | BOPAT | 37,4 | 37,5 | 3,8 | 10,2 | 29,2 | 43,8 | 12 |
| Body length: width, ratio in vivo | IS7 | 1,4 | 1,3 | 0,1 | 7,5 | 1,2 | 1,6 | 12 |
|  | BOPAT | 1,5 | 1,4 | 0,1 | 9,9 | 1,3 | 1,8 | 12 |
| Body length | IS7 | 37,0 | 36,7 | 2,0 | 5,4 | 33,8 | 40,7 | 19 |
|  | BOPAT | 48,0 | 46,9 | 4,0 | 8,3 | 41,7 | 56,0 | 19 |
| Body width | IS7 | 30,1 | 30,5 | 2,5 | 8,4 | 25,2 | 34,8 | 19 |
|  | BOPAT | 34,3 | 34,2 | 5,0 | 14,5 | 22,0 | 43,7 | 19 |
| Body length: width, ratio | IS7 | 1,2 | 1,2 | 0,1 | 7,1 | 1,1 | 1,5 | 19 |
|  | BOPAT | 1,4 | 1,4 | 0,2 | 10,9 | 1,2 | 1,9 | 19 |
| Frontal spine, length | IS7 | 4,5 | 4,5 | 1,0 | 23,0 | 3,0 | 5,8 | 9 |
|  | BOPAT | 6,1 | 5,9 | 0,7 | 12,1 | 4,9 | 7,1 | 9 |
| Anterior cell end to anterior end of adoral zone, distance | IS7 | 19,0 | 19,4 | 1,3 | 6,8 | 16,5 | 21,0 | 19 |
|  | BOPAT | 25,4 | 25,0 | 1,6 | 6,2 | 23,1 | 29,5 | 19 |
| Anterior cell end to posterior end of adoral zone, distance | IS7 | 26,5 | 26,6 | 1,8 | 6,7 | 23,1 | 29,3 | 19 |
|  | BOPAT | 33,5 | 33,1 | 2,2 | 6,5 | 30,4 | 38,9 | 19 |
| Posterior cell end to anterior end of adoral zone, distance | IS7 | 19,9 | 19,9 | 1,3 | 6,4 | 18,1 | 22,5 | 11 |
|  | BOPAT | 21,4 | 20,5 | 2,3 | 10,8 | 18,2 | 25,8 | 13 |
| Posterior cell end to posterior end of adoral zone, distance | IS7 | 10,6 | 10,2 | 1,4 | 13,1 | 8,6 | 13,5 | 11 |
|  | BOPAT | 10,6 | 10,0 | 1,8 | 17,0 | 8,8 | 14,8 | 13 |
| Posterior cell end to adoral zone distance, ratio | IS7 | 0,5 | 0,5 | 0,1 | 10,2 | 0,4 | 0,6 | 11 |
|  | BOPAT | 0,5 | 0,5 | 0,0 | 9,5 | 0,4 | 0,6 | 13 |
| Anterior end to epistomial fringe, distance | IS7 | 7,9 | 7,7 | 1,5 | 19,1 | 4,3 | 10,3 | 19 |
|  | BOPAT | 8,5 | 8,4 | 1,3 | 15,2 | 6,9 | 11,9 | 19 |
| Epistomial fringe on left, length | IS7 | 7,1 | 7,0 | 1,3 | 18,5 | 4,8 | 9,7 | 19 |
|  | BOPAT | 7,6 | 7,6 | 1,1 | 14,0 | 6,3 | 10,6 | 19 |
| Epistomial fringe on right, length | IS7 | 22,3 | 22,3 | 1,9 | 8,4 | 18,2 | 25,8 | 17 |
|  | BOPAT | 26,0 | 26,1 | 3,1 | 12,0 | 20,9 | 33,1 | 19 |
| Macronucleus, number | IS7 | 2,6 | 3,0 | 0,8 | 28,9 | 1,0 | 4,0 | 19 |
|  | BOPAT | 2,0 | 2,0 | 0,7 | 34,3 | 1,0 | 3,0 | 18 |
| Macronucleus, length | IS7 | 6,3 | 6,2 | 1,0 | 15,8 | 4,6 | 8,8 | 49 |
|  | BOPAT | 8,6 | 8,3 | 1,8 | 20,9 | 6,3 | 13,6 | 37 |
| Macronucleus, width | IS7 | 4,8 | 4,8 | 0,9 | 19,5 | 3,0 | 7,7 | 49 |
|  | BOPAT | 7,3 | 7,2 | 1,2 | 17,1 | 4,8 | 11,5 | 37 |
| Micronucleus, diameter | IS7 | 2,6 | 2,7 | 0,4 | 14,3 | 2,0 | 3,0 | 5 |
|  | BOPAT | 2,9 | 2,9 | 0,4 | 13,3 | 2,2 | 3,4 | 20 |
| Epistomial fringe pectinelles, number | IS7 | 23,7 | 24,0 | 1,9 | 7,9 | 21,0 | 27,0 | 19 |
|  | BOPAT | 23,9 | 24,0 | 1,6 | 6,7 | 21,0 | 27,0 | 19 |
| Epistomial fringe pectinelles on the left, number | IS7 | 8,3 | 8,0 | 1,0 | 12,0 | 6,0 | 10,0 | 19 |
|  | BOPAT | 8,2 | 8,0 | 0,6 | 7,4 | 7,0 | 9,0 | 19 |
| Epistomial fringe pectinelles on the right, number | IS7 | 15,5 | 15,0 | 1,6 | 10,2 | 13,0 | 18,0 | 19 |
|  | BOPAT | 15,7 | 16,0 | 1,2 | 7,9 | 13,0 | 18,0 | 19 |
| Epistomial fringe ciliary rows, highest number | IS7 | 5,0 | 5,0 | 0,0 | 0,0 | 5,0 | 5,0 | 19 |
|  | BOPAT | 5,0 | 5,0 | 0,0 | 0,0 | 5,0 | 5,0 | 19 |
| Adoral membranelles, number | IS7 | 9,0 | 9,0 | 0,0 | 0,0 | 9,0 | 9,0 | 19 |
|  | BOPAT | 9,0 | 9,0 | 0,0 | 0,0 | 9,0 | 9,0 | 19 |
| Longest membranelle, length | IS7 | 8,8 | 8,6 | 1,1 | 12,4 | 7,5 | 11,5 | 19 |
|  | BOPAT | 9,0 | 9,0 | 0,7 | 7,6 | 7,8 | 10,1 | 19 |
| Posterior spines, including ventrocaudal spine, number | IS7 | 7,0 | 7,0 | 0,0 | 0,0 | 7,0 | 7,0 | 19 |
|  | BOPAT | 7,0 | 7,0 | 0,0 | 0,0 | 7,0 | 7,0 | 19 |
| Preoral kineties, number | IS7 | 2,0 | 2,0 | 0,0 | 0,0 | 2,0 | 2,0 | 19 |
|  | BOPAT | 2,0 | 2,0 | 0,0 | 0,0 | 2,0 | 2,0 | 18 |
| Dikinetids in preoral kinety 1, number | IS7 | 20,3 | 21,0 | 2,9 | 14,3 | 15,0 | 26,0 | 17 |
|  | BOPAT | 22,4 | 23,0 | 1,9 | 8,4 | 19,0 | 25,0 | 17 |
| Dikinetids in preoral kinety 2, number | IS7 | 13,6 | 13,0 | 2,3 | 17,3 | 9,0 | 18,0 | 17 |
|  | BOPAT | 13,0 | 13,0 | 1,8 | 13,6 | 10,0 | 16,0 | 15 |
| Dikinetids in ventral kinety 1, number | IS7 | 5,5 | 6,0 | 0,7 | 13,1 | 4,0 | 6,0 | 17 |
|  | BOPAT | 5,6 | 6,0 | 0,5 | 8,9 | 5,0 | 6,0 | 16 |
| Dikinetids in ventral kinety 2, number | IS7 | 15,8 | 16,0 | 1,4 | 8,7 | 13,0 | 17,0 | 19 |
|  | BOPAT | 15,8 | 16,0 | 1,3 | 8,3 | 12,0 | 17,0 | 19 |
| Somatic kineties, including epistomial fringe ciliary rows, number | IS7 | 9,0 | 9,0 | 0,0 | 0,0 | 9,0 | 9,0 | 19 |
|  | BOPAT | 9,0 | 9,0 | 0,0 | 0,0 | 9,0 | 9,0 | 19 |
| Associated spine kineties, number | IS7 | 5,0 | 5,0 | 0,0 | 0,0 | 5,0 | 5,0 | 19 |
|  | BOPAT | 5,0 | 5,0 | 0,0 | 0,0 | 5,0 | 5,0 | 19 |
| Non-ciliated kineties on left side, number | IS7 | 3,0 | 3,0 | 0,0 | 0,0 | 3,0 | 3,0 | 19 |
|  | BOPAT | 3,0 | 3,0 | 0,0 | 0,0 | 3,0 | 3,0 | 12 |
| Non-ciliated kineties on right side, number | IS7 | 6,0 | 6,0 | 0,0 | 0,0 | 6,0 | 6,0 | 19 |
|  | BOPAT | 6,0 | 6,0 | 0,0 | 0,0 | 6,0 | 6,0 | 11 |
| Dikinetids in associated spine kinety 6, number | IS7 | 8,5 | 9,0 | 0,6 | 7,2 | 7,0 | 9,0 | 19 |
|  | BOPAT | 8,3 | 8,0 | 0,7 | 8,9 | 7,0 | 10,0 | 19 |
| Dikinetids in non-ciliated kinety 7, number | IS7 | 26,6 | 27,0 | 2,6 | 9,7 | 23,0 | 30,0 | 10 |
| Dikinetids in associated spine kinety 7, number | IS7 | 8,4 | 8,0 | 0,6 | 7,3 | 8,0 | 10,0 | 18 |
|  | BOPAT | 8,7 | 9,0 | 0,7 | 8,6 | 8,0 | 10,0 | 19 |
| Anterior fragment of kinety 8, length | IS7 | 13,1 | 13,2 | 2,0 | 14,9 | 10,1 | 16,2 | 19 |
|  | BOPAT | 21,0 | 21,2 | 2,3 | 10,9 | 17,3 | 26,7 | 18 |
| Dikinetids in anterior fragment of kinety 8, number | IS7 | 19,1 | 19,0 | 2,5 | 12,9 | 15,0 | 24,0 | 19 |
|  | BOPAT | 25,8 | 26,0 | 3,1 | 12,0 | 22,0 | 32,0 | 17 |
| Dikinetids in non-ciliated kinety 8, number | IS7 | 28,6 | 30,0 | 4,7 | 16,6 | 20,0 | 36,0 | 14 |
| Dikinetids in associated spine kinety 8, number | IS7 | 7,5 | 7,5 | 0,9 | 12,3 | 6,0 | 10,0 | 18 |
|  | BOPAT | 7,9 | 8,0 | 0,6 | 7,0 | 7,0 | 9,0 | 17 |
| Dikinetids in non-ciliated kinety 9, number | IS7 | 26,8 | 26,5 | 6,1 | 22,7 | 18,0 | 37,0 | 12 |
| Dikinetids in associated spine kinety 9, number | IS7 | 6,0 | 5,0 | 1,3 | 21,5 | 5,0 | 8,0 | 13 |
|  | BOPAT | 6,3 | 6,0 | 1,0 | 15,3 | 5,0 | 8,0 | 18 |

Measurements in µm based on protargol-impregnated specimens, except as noted (in vivo). M: mean, SD: standard deviation, CV: coefficient of variation, Min: minimum value, Max: maximum value, N: number of specimens examined.

**Table S7.** Morphometric data on *Saprodinium dentatum* VLKOV population.

| **Character** | **Mean** | **M** | **SD** | **CV** | **Min** | **Max** | **N** |
| --- | --- | --- | --- | --- | --- | --- | --- |
| Body length in vivo | 73,9 | 75,3 | 4,5 | 6,1 | 65,4 | 78,4 | 10 |
| Body width in vivo | 67,3 | 67,1 | 6,1 | 9,0 | 56,6 | 75,2 | 10 |
| Body length:width ratio in vivo | 1,1 | 1,1 | 0,0 | 4,3 | 1,0 | 1,2 | 10 |
| Body length | 61,0 | 60,5 | 4,6 | 7,5 | 56,0 | 72,0 | 12 |
| Body width | 57,6 | 58,0 | 4,3 | 7,4 | 51,0 | 65,0 | 12 |
| Body length:width ratio | 1,1 | 1,1 | 0,0 | 4,6 | 1,0 | 1,1 | 12 |
| Frontal spine, length | 9,3 | 9,0 | 2,2 | 23,7 | 6,0 | 13,0 | 11 |
| Anterior cell end to anterior end of adoral zone, distance | 30,0 | 30,5 | 3,4 | 11,2 | 26,0 | 37,0 | 12 |
| Anterior cell end to posterior end of adoral zone, distance | 42,2 | 41,5 | 4,8 | 11,3 | 36,0 | 53,0 | 12 |
| Posterior cell end to anterior end of adoral zone, distance | 30,6 | 30,2 | 2,1 | 6,7 | 28,1 | 34,0 | 6 |
| Posterior cell end to posterior end of adoral zone, distance | 15,4 | 15,2 | 1,3 | 8,5 | 13,8 | 17,6 | 6 |
| Posterior cell end to adoral zone distance, ratio | 0,5 | 0,5 | 0,1 | 12,0 | 0,4 | 0,6 | 6 |
| Anterior cell end to anterior end of epistomial fringe, distance | 11,6 | 11,5 | 1,9 | 16,7 | 9,0 | 16,0 | 12 |
| Epistomial fringe, right side, length | 39,3 | 40,0 | 3,6 | 9,2 | 32,0 | 44,0 | 12 |
| Macronuclear nodules, number | 1,6 | 1,5 | 0,7 | 42,2 | 1,0 | 3,0 | 12 |
| Length of Macronucleus | 10,5 | 10,0 | 2,5 | 23,9 | 6,0 | 16,0 | 19 |
| Width of macronucleus | 10,0 | 9,0 | 2,6 | 26,5 | 7,0 | 16,0 | 19 |
| Micronucleus, diameter | 4,2 | 4,0 | 0,4 | 9,3 | 4,0 | 5,0 | 12 |
| Epistomial fringe pectinelles, number | 31,2 | 32,0 | 2,1 | 6,9 | 27,0 | 34,0 | 10 |
| Epistomial fringe pectinelles on the left, number | 10,5 | 10,0 | 1,7 | 16,1 | 8,0 | 14,0 | 11 |
| Epistomial fringe pectinelles on the right, number | 21,2 | 21,0 | 1,9 | 9,2 | 17,0 | 24,0 | 11 |
| Epistomial fringe ciliary rows, highest number | 5,0 | 5,0 | 0,0 | 0,0 | 5,0 | 5,0 | 11 |
| Adoral membranelles, number | 9,0 | 9,0 | 0,0 | 0,0 | 9,0 | 9,0 | 17 |
| Longest membranelle, length | 12,1 | 12,0 | 1,5 | 12,5 | 10,0 | 14,0 | 12 |
| Posterior spines, including ventrocaudal spine, number | 8,4 | 8,0 | 0,5 | 6,0 | 8,0 | 9,0 | 11 |
| Preoral kineties, number | 2,0 | 2,0 | 0,0 | 0,0 | 2,0 | 2,0 | 12 |
| Dikinetids in preoral kinety 1, number | 33,9 | 34,0 | 1,3 | 4,0 | 32,0 | 35,0 | 7 |
| Dikinetids in preoral kinety 2, number | 25,7 | 26,0 | 0,8 | 2,9 | 25,0 | 27,0 | 7 |
| Dikinetids in ventral kinety 1, number | 21,4 | 20,0 | 2,9 | 13,4 | 19,0 | 27,0 | 7 |
| Dikinetids in ventral kinety 2, number | 31,7 | 31,0 | 2,1 | 6,5 | 30,0 | 35,0 | 6 |
| Supplementary ventral dikinetids, number | 4,2 | 4,0 | 0,4 | 9,8 | 4,0 | 5,0 | 6 |
| Somatic kineties, including epistomial fringe ciliary rows, number | 9,0 | 9,0 | 0,0 | 0,0 | 9,0 | 9,0 | 12 |
| Associated spine kineties, number | 4,0 | 4,0 | 0,0 | 0,0 | 4,0 | 4,0 | 12 |
| Non-ciliated kineties on left side, number | 3,0 | 3,0 | 0,0 | 0,0 | 3,0 | 3,0 | 12 |
| Non-ciliated kineties on right side, number | 6,0 | 6,0 | 0,0 | 0,0 | 6,0 | 6,0 | 12 |
| Dikinetids in associated spine kinety 6, number | 13,4 | 13,0 | 1,1 | 8,4 | 12,0 | 15,0 | 7 |
| Dikinetids in associated spine kinety 7, number | 11,7 | 12,0 | 0,8 | 6,5 | 11,0 | 13,0 | 7 |
| Dikinetids in anterior fragment of kinety 8, number | 94,4 | 95,0 | 8,2 | 8,7 | 82,0 | 110,0 | 14 |
| Dikinetids in associated spine kinety 8, number | 12,1 | 12,0 | 1,3 | 11,1 | 10,0 | 14,0 | 7 |
| Dikinetids in associated spine kinety 9, number | 10,1 | 10,0 | 0,7 | 6,8 | 9,0 | 11,0 | 7 |

Measurements in µm based on protargol-impregnated specimens, except as noted (in vivo). M: mean, SD: Standard deviation, CV: coefficient of variation, Min: minimum value, Max: maximum value, N: number of specimens examined.

**Table S****8.** Morphometric data on *Mircalla triangula* gen. nov., comb. nov. (MT) and *Mircalla polidorii* gen. nov., sp. nov. (MP).

| **Character** | **Species** | **Mean** | **M** | **SD** | **CV** | **Min** | **Max** | **N** |
| --- | --- | --- | --- | --- | --- | --- | --- | --- |
| Body length in vivo | MT | 33,8 | 33,6 | 1,4 | 4,1 | 31,7 | 36,8 | 12 |
|  | MP | 31,6 | 31,3 | 1,8 | 5,8 | 29,7 | 35,6 | 10 |
| Body width in vivo | MT | 23,6 | 23,6 | 1,7 | 7,2 | 20,3 | 27,1 | 12 |
|  | MP | 25,2 | 25,3 | 1,5 | 6,1 | 22,9 | 27,6 | 10 |
| Body length:width, ratio in vivo | MT | 1,4 | 1,5 | 0,1 | 6,5 | 1,3 | 1,7 | 12 |
|  | MP | 1,3 | 1,2 | 0,1 | 7,5 | 1,1 | 1,4 | 10 |
| Body length | MT | 25,4 | 25,0 | 1,9 | 7,4 | 22,2 | 28,9 | 19 |
|  | MP | 26,5 | 26,3 | 2,2 | 8,3 | 23,6 | 29,5 | 10 |
| Body width | MT | 16,1 | 16,2 | 1,7 | 10,8 | 12,5 | 19,5 | 19 |
|  | MP | 19,1 | 18,7 | 2,4 | 12,5 | 15,2 | 23,1 | 10 |
| Body length:width, ratio | MT | 1,6 | 1,6 | 0,1 | 6,7 | 1,4 | 1,9 | 19 |
|  | MP | 1,4 | 1,4 | 0,1 | 8,8 | 1,3 | 1,6 | 10 |
| Anterior end to anterior end of adoral zone, distance | MT | 10,3 | 10,1 | 1,3 | 12,2 | 8,2 | 13,9 | 19 |
|  | MP | 11,2 | 11,4 | 0,9 | 8,5 | 9,4 | 12,3 | 10 |
| Anterior end to posterior end of adoral zone, distance | MT | 17,8 | 17,4 | 1,6 | 9,2 | 16,0 | 22,8 | 19 |
|  | MP | 19,9 | 20,0 | 1,5 | 7,6 | 17,6 | 21,7 | 10 |
| Posterior cell end to anterior end of adoral zone, distance | MT | 15,5 | 15,8 | 1,0 | 6,5 | 13,6 | 16,8 | 12 |
|  | MP | 15,4 | 15,2 | 1,4 | 8,9 | 13,6 | 17,9 | 10,0 |
| Posterior cell end to posterior end of adoral zone, distance | MT | 6,7 | 6,1 | 1,3 | 18,8 | 5,3 | 8,6 | 10,0 |
|  | MP | 7,5 | 7,7 | 0,8 | 10,1 | 6,2 | 8,5 | 12 |
| Posterior adoral zone ratio | MT | 0,5 | 0,5 | 0,0 | 6,4 | 0,4 | 0,5 | 12 |
|  | MP | 0,4 | 0,4 | 0,1 | 11,8 | 0,4 | 0,5 | 10 |
| Epistomial fringe on left, length | MT | NA | NA | NA | NA | NA | NA | NA |
|  | MP | 2,2 | 2,1 | 0,3 | 14,7 | 1,9 | 2,9 | 10 |
| Epistomial fringe on right, length | MT | 9,7 | 9,4 | 1,5 | 15,3 | 7,2 | 12,7 | 16 |
|  | MP | 12,1 | 11,9 | 0,6 | 4,6 | 11,3 | 13,2 | 10 |
| Macronucleus, number | MT | 1,4 | 1,0 | 0,5 | 35,4 | 1,0 | 2,0 | 18 |
|  | MP | 1,6 | 2,0 | 0,5 | 32,3 | 1,0 | 2,0 | 10 |
| Macronucleus, length | MT | 6,9 | 6,6 | 1,7 | 24,1 | 4,4 | 10,2 | 28 |
|  | MP | 7,0 | 7,0 | 1,3 | 18,4 | 5,3 | 10,3 | 15 |
| Macronucleus, width | MT | 5,4 | 5,4 | 0,9 | 15,9 | 4,1 | 7,6 | 28 |
|  | MP | 6,4 | 6,4 | 0,8 | 11,8 | 5,4 | 7,9 | 15 |
| Micronucleus, diameter | MT | 2,6 | 2,6 | 0,2 | 9,5 | 2,3 | 3,0 | 18 |
|  | MP | 2,3 | 2,3 | 0,2 | 9,9 | 2,0 | 2,7 | 9 |
| Epistomial fringe pectinelles, number | MT | 7,1 | 7,0 | 0,7 | 10,1 | 6,0 | 8,0 | 16 |
|  | MP | 12,1 | 12,0 | 1,3 | 10,6 | 11,0 | 15,0 | 10 |
| Epistomial fringe pectinelles on the left, number | MT | NA | NA | NA | NA | NA | NA | NA |
|  | MP | 3,2 | 3,0 | 0,8 | 24,7 | 2,0 | 5,0 | 10 |
| Epistomial fringe pectinelles on the right, number | MT | 7,1 | 7,0 | 0,7 | 10,1 | 6,0 | 8,0 | 16 |
|  | MP | 8,9 | 9,0 | 0,7 | 8,3 | 8,0 | 10,0 | 10 |
| Epistomial fringe ciliary rows, highest number | MT | 4,0 | 4,0 | 0,0 | 0,0 | 4,0 | 4,0 | 15 |
|  | MP | 5,0 | 5,0 | 0,0 | 0,0 | 5,0 | 5,0 | 10 |
| Adoral membranelles, number | MT | 9,0 | 9,0 | 0,0 | 0,0 | 9,0 | 9,0 | 18 |
|  | MP | 9,0 | 9,0 | 0,0 | 0,0 | 9,0 | 9,0 | 10 |
| Longest membranelle, length | MT | 6,0 | 6,2 | 0,6 | 10,6 | 4,5 | 7,0 | 19 |
|  | MP | 8,2 | 8,3 | 0,9 | 10,7 | 6,9 | 9,5 | 10 |
| Preoral kineties, number | MT | 1,0 | 1,0 | 0,0 | 0,0 | 1,0 | 1,0 | 17 |
|  | MP | 2,0 | 2,0 | 0,0 | 0,0 | 2,0 | 2,0 | 10 |
| Dikinetids in preoral kinety 1, number | MT | 13,4 | 13,0 | 1,5 | 11,5 | 10,0 | 15,0 | 17 |
|  | MP | 8,9 | 9,0 | 0,9 | 9,8 | 8,0 | 10,0 | 10 |
| Dikinetids in preoral kinety 2, number | MT | 0,0 | 0,0 | 0,0 | 0,0 | 0,0 | 0,0 | 0 |
|  | MP | 4,8 | 5,0 | 0,4 | 8,4 | 4,0 | 5,0 | 6 |
| Dikinetids in ventral kinety 1, number | MT | 3,9 | 4,0 | 0,3 | 8,8 | 3,0 | 4,0 | 16 |
|  | MP | 4,4 | 4,0 | 0,5 | 11,9 | 4,0 | 5,0 | 9 |
| Dikinetids in ventral kinety 2, number | MT | 3,0 | 3,0 | 0,0 | 0,0 | 3,0 | 3,0 | 15 |
|  | MP | 10,1 | 10,0 | 1,3 | 12,6 | 7,0 | 11,0 | 9 |
| Supplementary ventral dikinetids, number | MT | 3,7 | 4,0 | 0,5 | 13,0 | 3,0 | 4,0 | 13 |
|  | MP | 0,0 | 0,0 | 0,0 | 0,0 | 0,0 | 0,0 | 0 |
| Somatic kineties, including epistomial fringe ciliary rows, number | MT | 9,0 | 9,0 | 0,0 | 0,0 | 9,0 | 9,0 | 9 |
|  | MP | 9,0 | 9,0 | 0,0 | 0,0 | 9,0 | 9,0 | 8 |
| Associated spine kineties, number | MT | 5,0 | 5,0 | 0,0 | 0,0 | 5,0 | 5,0 | 13 |
|  | MP | 4,0 | 4,0 | 0,0 | 0,0 | 4,0 | 4,0 | 7 |
| Non-ciliated kineties on left side, number | MT | 3,0 | 3,0 | 0,0 | 0,0 | 3,0 | 3,0 | 15 |
|  | MP | 3,0 | 3,0 | 0,0 | 0,0 | 3,0 | 3,0 | 8 |
| Non-ciliated kineties on right side, number | MT | 6,0 | 6,0 | 0,0 | 0,0 | 6,0 | 6,0 | 15 |
|  | MP | 6,0 | 6,0 | 0,0 | 0,0 | 6,0 | 6,0 | 7 |
| Dikinetids in associated spine kinety 6, number | MT | 4,2 | 4,0 | 0,5 | 13,0 | 4,0 | 6,0 | 18 |
|  | MP | 4,9 | 5,0 | 0,4 | 7,8 | 4,0 | 5,0 | 7 |
| Dikinetids in associated spine kinety 7, number | MT | 4,9 | 5,0 | 1,0 | 20,9 | 3,0 | 6,0 | 18 |
|  | MP | 4,7 | 5,0 | 0,5 | 10,7 | 4,0 | 5,0 | 9 |
| Anterior fragment of kinety 8, length | MT | 7,1 | 7,1 | 0,7 | 9,9 | 5,9 | 8,3 | 17 |
|  | MP | 9,5 | 9,7 | 0,8 | 8,1 | 8,0 | 10,3 | 10 |
| Dikinetids in anterior fragment of kinety 8, number | MT | 7,4 | 7,0 | 0,6 | 8,2 | 6,0 | 8,0 | 17 |
|  | MP | 12,6 | 12,5 | 0,7 | 5,5 | 12,0 | 14,0 | 10 |
| Dikinetids in associated spine kinety 8, number | MT | 4,4 | 4,5 | 0,6 | 14,2 | 3,0 | 5,0 | 16 |
|  | MP | 4,1 | 4,0 | 0,8 | 19,0 | 3,0 | 5,0 | 9 |
| Dikinetids in associated spine kinety 9, number | MT | 2,0 | 2,0 | 0,0 | 0,0 | 2,0 | 2,0 | 14 |
|  | MP | 2,4 | 2,0 | 0,5 | 21,6 | 2,0 | 3,0 | 9 |

Measurements in µm based on protargol-impregnated specimens, except as noted (in vivo). M: mean, SD: Standard deviation, CV: coefficient of variation, Min: minimum value, Max: maximum value, N: number of specimens examined.

**Table S9.** Morphometric data on *Epalxella exigua* LAUFENJ population.

| **Character** | **Mean** | **M** | **SD** | **CV** | **Min** | **Max** | **N** |
| --- | --- | --- | --- | --- | --- | --- | --- |
| Body length in vivo | 33,1 | 33,1 | 2,4 | 7,3 | 30,2 | 36,8 | 5 |
| Body width in vivo | 25,2 | 24,6 | 2,2 | 8,7 | 23,1 | 28,8 | 5 |
| Body length:width, ratio in vivo | 1,3 | 1,3 | 0,1 | 8,0 | 1,1 | 1,4 | 5 |
| Body length | 24,6 | 24,8 | 0,7 | 3,0 | 23,7 | 25,4 | 6 |
| Body width | 17,3 | 17,7 | 1,1 | 6,6 | 15,0 | 18,0 | 6 |
| Body length:width, ratio | 1,4 | 1,4 | 0,1 | 5,9 | 1,3 | 1,6 | 6 |
| Anterior end to anterior end of adoral zone, distance | 11,7 | 11,7 | 0,8 | 7,0 | 10,7 | 13,0 | 6 |
| Anterior end to posterior end of adoral zone, distance | 20,2 | 20,2 | 0,6 | 2,8 | 19,5 | 21,2 | 6 |
| Posterior cell end to anterior end of adoral zone, distance | 13,4 | 13,4 | 0,9 | 6,8 | 12,2 | 14,7 | 6 |
| Posterior cell end to posterior end of adoral zone, distance | 4,2 | 4,0 | 0,7 | 16,2 | 3,4 | 5,1 | 6 |
| Posterior cell end to adoral zone distance, ratio | 0,3 | 0,3 | 0,0 | 10,6 | 0,3 | 0,4 | 6 |
| Epistomial fringe on left, length | 10,0 | 9,4 | 2,1 | 21,1 | 7,4 | 12,5 | 5 |
| Epistomial fringe on right, length | 2,7 | 2,5 | 0,7 | 26,1 | 2,1 | 3,9 | 6 |
| Macronucleus, number | 1,8 | 2,0 | 0,8 | 41,1 | 1,0 | 3,0 | 6 |
| Macronucleus, length | 5,7 | 5,2 | 1,7 | 29,3 | 3,9 | 9,1 | 11 |
| Macronucleus, width | 4,4 | 4,4 | 0,9 | 20,1 | 2,9 | 6,4 | 11 |
| Micronucleus, diameter | 2,0 | 2,0 | 0,2 | 7,9 | 1,8 | 2,2 | 6 |
| Pectinelles of epistomial fringe, number | 7,8 | 8,0 | 0,8 | 9,6 | 7,0 | 9,0 | 6 |
| Epistomial fringe ciliary rows, highest number | 5,0 | 5,0 | 0,0 | 0,0 | 5,0 | 5,0 | 6 |
| Adoral membranelles, number | 9,0 | 9,0 | 0,0 | 0,0 | 9,0 | 9,0 | 6 |
| Longest membranelle, length | 7,1 | 6,9 | 0,6 | 8,9 | 6,6 | 8,2 | 6 |
| Preoral kineties, number | 2,0 | 2,0 | 0,0 | 0,0 | 2,0 | 2,0 | 6 |
| Dikinetids in preoral kinety 1, number | 3,3 | 3,0 | 0,6 | 17,3 | 3,0 | 4,0 | 3 |
| Dikinetids in preoral kinety 2, number | 5,3 | 5,0 | 0,5 | 9,7 | 5,0 | 6,0 | 6 |
| Dikinetids in ventral kinety 1, number | 6,2 | 6,0 | 0,4 | 7,2 | 6,0 | 7,0 | 5 |
| Supplementary ventral dikinetids, number | 2,0 | 2,0 | 0,0 | 0,0 | 2,0 | 2,0 | 6 |
| Somatic kineties, including epistomial fringe ciliary rows, number | 9,0 | 9,0 | 0,0 | 0,0 | 9,0 | 9,0 | 6 |
| Associated spine kineties, number | 4,0 | 4,0 | 0,0 | 0,0 | 4,0 | 4,0 | 6 |
| Non-ciliated kineties on left side, number | 3,0 | 3,0 | 0,0 | 0,0 | 3,0 | 3,0 | 6 |
| Non-ciliated kineties on right side, number | 6,0 | 6,0 | 0,0 | 0,0 | 6,0 | 6,0 | 6 |
| Dikinetids in associated spine kinety 6, number | 7,8 | 8,0 | 0,4 | 5,2 | 7,0 | 8,0 | 6 |
| Dikinetids in associated spine kinety 7, number | 8,0 | 8,0 | 0,9 | 11,2 | 7,0 | 9,0 | 6 |
| Anterior fragment of kinety 8, length | 6,5 | 6,4 | 0,6 | 9,0 | 5,6 | 7,4 | 6 |
| Dikinetids in anterior fragment of kinety 8, number | 8,0 | 8,0 | 0,7 | 8,8 | 7,0 | 9,0 | 5 |
| Dikinetids in associated spine kinety 8, number | 6,2 | 6,0 | 0,4 | 6,6 | 6,0 | 7,0 | 6 |
| Dikinetids in associated spine kinety 9, number | 4,5 | 4,5 | 0,5 | 12,2 | 4,0 | 5,0 | 6 |

Measurements in µm based on protargol-impregnated specimens, except as noted (in vivo). M: mean, SD: Standard deviation, CV: coefficient of variation, Min: minimum value, Max: maximum value, N: number of specimens examined.

**Table S10.** Morphometric data on *Mylestoma bipartitum* KLAGOS (K), *Mylestoma monodontum* sp. nov. FRESHO (F) and FARO (FA), and *Limnomylestoma shuriken* gen. nov., sp. nov. SLATINA (S).

| **Character** | **Species** | **Strain** | **Mean** | **M** | **SD** | **CV** | **Min** | **Max** | **N** |
| --- | --- | --- | --- | --- | --- | --- | --- | --- | --- |
| Body length in vivo | *M. bipartitum* | K | 36,7 | 36,9 | 2,3 | 6,2 | 33,4 | 41,8 | 11 |
|  | *M. monodontum* | F | 21,7 | 21,3 | 2,0 | 9,2 | 18,9 | 25,0 | 17 |
|  |  | FA | 20,7 | 21,3 | 1,4 | 6,8 | 18,3 | 22,5 | 12 |
|  | *L. shuriken* | S | 28,0 | 27,2 | 2,9 | 10,5 | 24,2 | 33,3 | 10 |
| Body width in vivo | *M. bipartitum* | K | 27,5 | 27,8 | 2,5 | 9,1 | 24,2 | 31,2 | 11 |
|  | *M. monodontum* | F | 18,0 | 18,0 | 2,3 | 13,0 | 13,6 | 21,2 | 17 |
|  |  | FA | 16,8 | 16,7 | 1,7 | 10,0 | 14,3 | 19,8 | 12 |
|  | *L. shuriken* | S | 21,9 | 22,4 | 2,2 | 10,0 | 18,3 | 25,9 | 10 |
| Body length:width ratio in vivo | *M. bipartitum* | K | 1,3 | 1,3 | 0,1 | 6,3 | 1,2 | 1,5 | 11 |
|  | *M. monodontum* | F | 1,2 | 1,2 | 0,1 | 6,4 | 1,1 | 1,4 | 17 |
|  |  | FA | 1,2 | 1,2 | 0,1 | 4,5 | 1,1 | 1,3 | 12 |
|  | *L. shuriken* | S | 1,3 | 1,3 | 0,1 | 5,7 | 1,2 | 1,4 | 10 |
| Body length | *M. bipartitum* | K | 29,5 | 29,6 | 2,7 | 9,1 | 22,8 | 34,1 | 25 |
|  | *M. monodontum* | F | 19,8 | 19,7 | 2,2 | 10,9 | 15,9 | 25,4 | 17 |
|  |  | FA | 19,8 | 20,0 | 2,2 | 11,2 | 16,0 | 24,0 | 11 |
|  | *L. shuriken* | S | 19,2 | 19,2 | 1,9 | 10,0 | 15,8 | 23,5 | 42 |
| Body width | *M. bipartitum* | K | 21,3 | 21,4 | 2,2 | 10,3 | 16,9 | 25,4 | 24 |
|  | *M. monodontum* | F | 16,4 | 17,1 | 2,2 | 13,7 | 13,2 | 21,1 | 17 |
|  |  | FA | 16,7 | 17,0 | 1,3 | 8,1 | 14,0 | 19,0 | 11 |
|  | *L. shuriken* | S | 15,1 | 15,4 | 1,5 | 9,9 | 12,2 | 18,6 | 41 |
| Body length:width ratio | *M. bipartitum* | K | 1,4 | 1,4 | 0,1 | 7,4 | 1,2 | 1,6 | 24 |
|  | *M. monodontum* | F | 1,2 | 1,2 | 0,1 | 6,7 | 1,1 | 1,4 | 17 |
|  |  | FA | 1,2 | 1,2 | 0,1 | 5,6 | 1,1 | 1,3 | 11 |
|  | *L. shuriken* | S | 1,3 | 1,3 | 0,1 | 9,1 | 1,0 | 1,5 | 41 |
| Anterior end to anterior end of adoral zone, distance | *M. bipartitum* | K | 15,7 | 15,8 | 1,7 | 10,9 | 12,9 | 20,5 | 25 |
|  | *M. monodontum* | F | 9,0 | 8,6 | 1,4 | 15,7 | 6,3 | 11,8 | 17 |
|  |  | FA | 9,8 | 10,0 | 1,5 | 15,7 | 6,0 | 12,0 | 11 |
|  | *L. shuriken* | S | 10,4 | 10,3 | 1,4 | 13,3 | 8,1 | 13,2 | 42 |
| Anterior end to posterior end of adoral zone, distance | *M. bipartitum* | K | 25,3 | 25,4 | 2,3 | 9,1 | 19,6 | 30,5 | 25 |
|  | *M. monodontum* | F | 17,5 | 17,8 | 1,7 | 9,6 | 14,1 | 20,9 | 17 |
|  |  | FA | 17,4 | 17,0 | 1,7 | 10,1 | 14,0 | 20 | 11 |
|  | *L. shuriken* | S | 16,9 | 17,0 | 1,6 | 9,6 | 13,4 | 19,7 | 42 |
| Posterior cell end to anterior end of adoral zone, distance | *M. bipartitum* | K | 13,9 | 13,6 | 1,7 | 12,1 | 9,1 | 16,5 | 25,0 |
|  | *M. monodontum* | F | 10,8 | 10,6 | 1,2 | 10,8 | 9,5 | 13,6 | 17 |
|  |  | FA | 10,0 | 10,0 | 1,3 | 13,4 | 8,0 | 12 | 11 |
|  | *L. shuriken* | S | 8,8 | 8,9 | 1,0 | 11,3 | 6,9 | 11,6 | 42,0 |
| Posterior cell end to posterior end of adoral zone, distance | *M. bipartitum* | K | 4,2 | 4,2 | 1,1 | 26,4 | 1,8 | 6,3 | 25,0 |
|  | *M. monodontum* | F | 2,3 | 2,0 | 0,8 | 37,1 | 1,3 | 5 | 17 |
|  |  | FA | 2,5 | 2,0 | 1,2 | 49,4 | 1,0 | 5 | 11 |
|  | *L. shuriken* | S | 2,3 | 2,3 | 0,7 | 29,3 | 1,2 | 4,2 | 42,0 |
| Posterior cell end to adoral zone distance, ratio | *M. bipartitum* | K | 0,3 | 0,3 | 0,1 | 22,2 | 0,1 | 0,4 | 25,0 |
|  | *M. monodontum* | F | 0,2 | 0,2 | 0,1 | 28,4 | 0,1 | 0,3 | 17 |
|  |  | FA | 0,2 | 0,2 | 0,1 | 37,8 | 0,1 | 0,4 | 11 |
|  | *L. shuriken* | S | 0,3 | 0,3 | 0,1 | 23,4 | 0,1 | 0,4 | 42,0 |
| Macronucleus, number | *M. bipartitum* | K | 1,0 | 1,0 | 0,0 | 0,0 | 1,0 | 1,0 | 25 |
|  | *L. shuriken* | S | 1,0 | 1,0 | 0,0 | 0,0 | 1,0 | 1,0 | 43 |
| Macronucleus, length | *M. bipartitum* | K | 8,5 | 8,4 | 0,9 | 10,8 | 6,9 | 10,0 | 23 |
|  | *M. monodontum* | F | 10,1 | 10,1 | 1,7 | 16,7 | 7,7 | 12,7 | 17 |
|  |  | FA | 9,7 | 9,0 | 1,8 | 18,4 | 8,0 | 14,0 | 11 |
|  | *L. shuriken* | S | 7,3 | 7,2 | 1,2 | 16,1 | 4,4 | 10,4 | 43 |
| Macronucleus, width | *M. bipartitum* | K | 7,0 | 6,6 | 1,3 | 18,8 | 4,8 | 9,8 | 23 |
|  | *M. monodontum* | F | 5,4 | 5,3 | 1,2 | 21,4 | 4,2 | 7,4 | 17 |
|  |  | FA | 4,7 | 5,0 | 1,0 | 21,3 | 3,0 | 6,0 | 11 |
|  | *L. shuriken* | S | 5,4 | 5,3 | 0,7 | 13,1 | 4,4 | 7,6 | 43 |
| Micronucleus, diameter | *M. bipartitum* | K | 2,8 | 2,9 | 0,5 | 18,2 | 2,0 | 3,6 | 20 |
|  | *M. monodontum* | F | 2,1 | 2,1 | 0,4 | 18,8 | 1,7 | 2,8 | 13 |
|  |  | FA | 2,1 | 2,0 | 0,6 | 27,0 | 1,0 | 3,0 | 10 |
|  | *L. shuriken* | S | 2,2 | 2,1 | 0,3 | 15,0 | 1,7 | 3,1 | 36 |
| Pectineles in epistomial fringe, number | *M. bipartitum* | K | 8,4 | 8,5 | 0,6 | 7,7 | 7,0 | 9,0 | 14 |
|  | *M. monodontum* | F | 4,0 | 4,0 | 0,0 | 0,0 | 4,0 | 4,0 | 10 |
|  |  | FA | 4,0 | 4,0 | 0,0 | 0,0 | 4,0 | 4,0 | 9 |
|  | *L. shuriken* | S | 3,8 | 4,0 | 0,4 | 10,7 | 3,0 | 4,0 | 30 |
| Epistomial fringe ciliary rows, highest number | *M. bipartitum* | K | 5,0 | 5,0 | 0,0 | 0,0 | 5,0 | 5,0 | 13 |
|  | *M. monodontum* | F | 5,0 | 5,0 | 0,0 | 0,0 | 5,0 | 5,0 | 10 |
|  |  | FA | 5,0 | 5,0 | 0,0 | 0,0 | 5,0 | 5,0 | 9 |
|  | *L. shuriken* | S | 5,0 | 5,0 | 0,0 | 0,0 | 5,0 | 5,0 | 12 |
| Adoral membranelles, number | *M. bipartitum* | K | 8,9 | 9,0 | 0,3 | 3,2 | 8,0 | 9,0 | 23 |
|  | *M. monodontum* | F | 7,9 | 8,0 | 0,3 | 4,3 | 7,0 | 8,0 | 16 |
|  |  | FA | 7,5 | 7,0 | 0,5 | 7,0 | 7,0 | 8,0 | 11 |
|  | *L. shuriken* | S | 9,0 | 9,0 | 0,0 | 0,0 | 9,0 | 9,0 | 37 |
| Longest membranelle, length | *M. bipartitum* | K | 10,7 | 10,6 | 1,4 | 12,8 | 8,6 | 13,2 | 24 |
|  | *M. monodontum* | F | 6,6 | 6,4 | 0,9 | 13,4 | 5,3 | 8,8 | 17 |
|  |  | FA | 6,2 | 6,0 | 0,8 | 12,1 | 5,0 | 7,0 | 11 |
|  | *L. shuriken* | S | 6,3 | 6,4 | 0,6 | 9,3 | 4,9 | 7,6 | 41 |
| Shortest membranelle, length | *L. shuriken* | S | 4,0 | 4,0 | 0,0 | 0,0 | 4,0 | 4,0 | 38 |
|  | *M. monodontum* | F | 2,0 | 1,9 | 0,3 | 15,3 | 1,5 | 2,5 | 17 |
|  |  | FA | 2,6 | 3,0 | 0,5 | 19,1 | 2,0 | 3,0 | 11 |
| Adoral zone, length | *M. monodontum* | F | 8,5 | 8,4 | 0,8 | 9,7 | 6,9 | 9,6 | 17 |
|  |  | FA | 7,5 | 7,0 | 0,7 | 9,1 | 7,0 | 9,0 | 11 |
| Preoral kineties, number | *M. bipartitum* | K | 2,0 | 2,0 | 0,0 | 0,0 | 2,0 | 2,0 | 10 |
|  | *M. monodontum* | F | 2,0 | 2,0 | 0,0 | 0,0 | 2,0 | 2,0 | 5 |
|  |  | FA | 2,0 | 2,0 | 0,0 | 0,0 | 2,0 | 2,0 | 2 |
|  | *L. shuriken* | S | 2,0 | 2,0 | 0,0 | 0,0 | 2,0 | 2,0 | 22 |
| Dikinetids in preoral kinety 1, number | *M. bipartitum* | K | 9,7 | 10,0 | 0,8 | 8,1 | 8,0 | 11,0 | 11 |
|  | *M. monodontum* | F | 6,0 | 6,0 | 0,0 | 0,0 | 6,0 | 6,0 | 5 |
|  |  | FA | 5,5 | 5,5 | 0,6 | 10,5 | 5,0 | 6,0 | 4 |
|  | *L. shuriken* | S | 4,9 | 5,0 | 0,4 | 7,3 | 4,0 | 5,0 | 28 |
| Dikinetids in preoral kinety 2, number | *M. bipartitum* | K | 7,2 | 7,0 | 1,3 | 17,4 | 6,0 | 9,0 | 11 |
|  | *M. monodontum* | F | 3,0 | 3,0 | 0,0 | 0,0 | 3,0 | 3,0 | 6 |
|  |  | FA | 3,0 | 3,0 | 0,0 | 0,0 | 3,0 | 3,0 | 2 |
|  | *L. shuriken* | S | 3,3 | 3,0 | 0,6 | 16,7 | 2,0 | 4,0 | 24 |
| Dikinetids in ventral kinety 1, number | *M. bipartitum* | K | 2,7 | 3,0 | 0,6 | 21,7 | 2,0 | 3,0 | 3 |
|  | *M. monodontum* | F | 3,9 | 4,0 | 0,4 | 9,8 | 3,0 | 4,0 | 7 |
|  |  | FA | 4,0 | 4,0 | 0,0 | 0,0 | 4,0 | 4,0 | 6 |
|  | *L. shuriken* | S | 6,0 | 6,0 | 0,0 | 0,0 | 6,0 | 6,0 | 25 |
| Supplementary ventral dikinetids, number | *M. bipartitum* | K | 1,0 | 1,0 | 0,0 | 0,0 | 1,0 | 1,0 | 13 |
| Somatic kineties, number | *M. bipartitum* | K | 9,0 | 9,0 | 0,0 | 0,0 | 9,0 | 9,0 | 15 |
|  | *M. monodontum* | F | 7,0 | 7,0 | 0,0 | 0,0 | 7,0 | 7,0 | 6 |
|  |  | FA | 7,0 | 7,0 | 0,0 | 0,0 | 7,0 | 7,0 | 6 |
|  | *L. shuriken* | S | 7,0 | 7,0 | 0,0 | 0,0 | 7,0 | 7,0 | 9 |
| Non-ciliated kineties on left side, number | *M. bipartitum* | K | 5,0 | 5,0 | 0,0 | 0,0 | 5,0 | 5,0 | 12 |
|  | *M. monodontum* | F | 2,0 | 2,0 | 0,0 | 0,0 | 2,0 | 2,0 | 6 |
|  |  | FA | 2,0 | 2,0 | 0,0 | 0,0 | 2,0 | 2,0 | 6 |
|  | *L. shuriken* | S | 5,0 | 5,0 | 0,0 | 0,0 | 5,0 | 5,0 | 11 |
| Non-ciliated kineties on right side, number | *M. bipartitum* | K | 4,0 | 4,0 | 0,0 | 0,0 | 4,0 | 4,0 | 11 |
|  | *M. monodontum* | F | 5,0 | 5,0 | 0,0 | 0,0 | 5,0 | 5,0 | 6 |
|  |  | FA | 5,0 | 5,0 | 0,0 | 0,0 | 5,0 | 5,0 | 6 |
|  | *L. shuriken* | S | 2,0 | 2,0 | 0,0 | 0,0 | 2,0 | 2,0 | 9 |
| Rows of caudal cirri, number | *M. bipartitum* | K | 2,0 | 0,0 | 0,0 | 2,0 | 2,0 | 17,0 | 18 |
|  | *M. monodontum* | F | 2,0 | 2,0 | 0,0 | 0,0 | 2,0 | 2,0 | 17 |
|  |  | FA | 2,0 | 2,0 | 0,0 | 0,0 | 2,0 | 2,0 | 11 |
|  | *L. shuriken* | S | 2,0 | 2,0 | 0,0 | 0,0 | 2,0 | 2,0 | 35 |
| Caudal cirri, length, in vivo | *M. bipartitum* | K | 44,3 | 43,6 | 6,0 | 13,5 | 37,6 | 56,0 | 10 |
|  | *M. monodontum* | F | 24,4 | 25,1 | 4,1 | 16,8 | 17,2 | 31,0 | 16 |
|  |  | FA | 24,6 | 25,3 | 3,0 | 12,2 | 19,8 | 28,4 | 10 |
|  | *L. shuriken* | S | 19,3 | 19,3 | 3,1 | 16,2 | 15,2 | 24,1 | 6 |
| Kinetids in each caudal cirrus, number | *M. bipartitum* | K | 9,6 | 10,0 | 0,7 | 7,6 | 8,0 | 10,0 | 9 |
|  | *M. monodontum* | F | 6,0 | 6,0 | 0,0 | 0,0 | 6,0 | 6,0 | 8 |
|  |  | FA | 6,0 | 6,0 | 0,0 | 0,0 | 6,0 | 6,0 | 8 |
|  | *L. shuriken* | S | 8,0 | 8,0 | 0,0 | 0,0 | 8,0 | 8,0 | 17 |

Measurements in µm based on protargol-impregnated specimens, except as noted (in vivo). M: mean, SD: Standard deviation, CV: coefficient of variation, Min: minimum value, Max: maximum value, N: number of specimens examined.

**Table S11.** Morphometric data on *Tostonella uncinata* gen. nov., sp. nov. BUL7A population.

| **Character** | **Mean** | **M** | **SD** | **CV** | **Min** | **Max** | **N** |
| --- | --- | --- | --- | --- | --- | --- | --- |
| Body length in vivo | 31,3 | 31,5 | 1,6 | 0,1 | 28,1 | 33,6 | 17 |
| Body width in vivo | 30,8 | 31,1 | 1,7 | 0,1 | 28,1 | 33,1 | 17 |
| Body length:width, ratio in vivo | 1,0 | 1,0 | 0,0 | 0,0 | 1,0 | 1,1 | 17 |
| Body length | 28,0 | 28,3 | 1,3 | 0,0 | 25,3 | 29,5 | 12 |
| Body width | 27,3 | 27,1 | 1,9 | 0,1 | 24,8 | 29,7 | 11 |
| Body length:width, ratio | 1,0 | 1,0 | 0,1 | 0,0 | 0,9 | 1,1 | 11 |
| Anterior cell end to posterior end of frontal band, distance | 6,9 | 6,3 | 1,6 | 0,2 | 4,9 | 9,9 | 11 |
| Anterior end to anterior end of adoral zone, distance | 16,4 | 16,9 | 1,4 | 0,1 | 14,1 | 18,1 | 12 |
| Anterior end to posterior end of adoral zone, distance | 24,9 | 25,1 | 1,4 | 0,1 | 22,2 | 26,7 | 12 |
| Posterior cell end to anterior end of adoral zone, distance | 11,6 | 11,4 | 1,4 | 0,1 | 10,1 | 15,4 | 12,0 |
| Posterior cell end to posterior end of adoral zone, distance | 3,1 | 2,6 | 1,5 | 0,5 | 1,7 | 7,3 | 12,0 |
| Posterior cell end to adoral zone distance, ratio | 0,3 | 0,2 | 0,1 | 0,3 | 0,2 | 0,5 | 12,0 |
| Macronucleus, number | 1,0 | 1,0 | 0,0 | 0,0 | 1,0 | 1,0 | 12 |
| Macronucleus, diameter | 8,8 | 9,0 | 1,0 | 0,1 | 6,8 | 10,6 | 12 |
| Micronucleus, diameter | 2,0 | 2,0 | 0,1 | 0,1 | 1,9 | 2,2 | 10 |
| Pectinelles of epistomial fringe, number | 5,0 | 5,0 | 0,0 | 0,0 | 5,0 | 5,0 | 8 |
| Epistomial fringe ciliary rows, highest number | 5,0 | 5,0 | 0,0 | 0,0 | 5,0 | 5,0 | 8 |
| Adoral membranelles, number | 10,0 | 10,0 | 0,0 | 0,0 | 10,0 | 10,0 | 12 |
| Longest membranelle, length | 8,8 | 9,1 | 1,0 | 0,1 | 7,1 | 10,0 | 12 |
| Preoral kineties, number | 2,0 | 2,0 | 0,0 | 0,0 | 2,0 | 2,0 | 12 |
| Dikinetids in perioral kinety 1, number | 6,9 | 7,0 | 1,2 | 0,2 | 5,0 | 8,0 | 7 |
| Dikinetids in perioral kinety 2, number | 7,3 | 7,5 | 1,0 | 0,1 | 6,0 | 8,0 | 4 |
| Dikinetids in ventral kinety 1, number | 9,8 | 10,0 | 0,4 | 0,0 | 9,0 | 10,0 | 5 |
| Somatic kineties, number | 4,0 | 4,0 | 0,0 | 0,0 | 4,0 | 4,0 | 4 |
| Non-ciliated kineties on left side, number | 1,0 | 1,0 | 0,0 | 0,0 | 1,0 | 1,0 | 4 |
| Non-ciliated kineties on right side, number | 3,0 | 3,0 | 0,0 | 0,0 | 3,0 | 3,0 | 4 |
| Rows of caudal cilia, number | 2,0 | 2,0 | 0,0 | 0,0 | 2,0 | 2,0 | 11 |
| Caudal cirri, length, in vivo | 25,5 | 23,7 | 4,7 | 0,2 | 21,6 | 38,2 | 11 |
| Kinetids in each caudal cirrus, number | 6,7 | 6,0 | 1,2 | 0,2 | 6,0 | 8,0 | 3 |

Measurements in µm based on protargol-impregnated specimens, except as noted (in vivo). M: mean, SD: Standard deviation, CV: coefficient of variation, Min: minimum value, Max: maximum value, N: number of specimens examined.

**Table S12.** Morphometric data on *Discomorphella pectinata* MOKOT and BOTAN populations.

| **Character** | **Strain** | **Mean** | **M** | **SD** | **CV** | **Min** | **Max** | **N** |
| --- | --- | --- | --- | --- | --- | --- | --- | --- |
| Body length, in vivo | MOKOT | 72,7 | 71,2 | 4,7 | 6,4 | 66,9 | 78,7 | 9,0 |
|  | BOTAN | 74,2 | 74,5 | 4,4 | 5,9 | 67,0 | 80,0 | 12,0 |
| Body width, in vivo | MOKOT | 64,6 | 63,7 | 5,9 | 9,1 | 56,4 | 71,0 | 9,0 |
|  | BOTAN | 72,0 | 72,5 | 4,6 | 6,4 | 63,0 | 79,0 | 12,0 |
| Body length:width, ratio in vivo | MOKOT | 1,2 | 1,2 | 0,1 | 5,0 | 1,1 | 1,2 | 5,0 |
|  | BOTAN | 1,0 | 1,0 | 0,1 | 5,1 | 0,9 | 1,1 | 12,0 |
| Body spines, number | MOKOT | 3,0 | 3,0 | 0,0 | 0,0 | 3,0 | 3,0 | 13,0 |
|  | BOTAN | 3,0 | 3,0 | 0,0 | 0,0 | 3,0 | 3,0 | 12,0 |
| Frontal spine, length | MOKOT | 11,5 | 11,5 | 1,3 | 11,2 | 10,0 | 13,0 | 4,0 |
|  | BOTAN | 15,1 | 15,0 | 1,2 | 7,7 | 13,0 | 17,0 | 12,0 |
| Lateral spine, length | MOKOT | 16,7 | 16,0 | 2,1 | 12,5 | 15,0 | 19,0 | 3,0 |
|  | BOTAN | 22,0 | 21,0 | 1,7 | 7,9 | 21,0 | 24,0 | 3,0 |
| Posterior spine, length | MOKOT | 21,0 | 21,0 | 2,0 | 9,5 | 19,0 | 23,0 | 3,0 |
|  | BOTAN | 26,0 | 25,5 | 3,3 | 12,8 | 21,0 | 30,0 | 10,0 |
| Body length | MOKOT | 62,5 | 62,3 | 4,9 | 7,8 | 53,1 | 69,8 | 13,0 |
|  | BOTAN | 60,6 | 61,7 | 3,8 | 6,3 | 54,2 | 64,3 | 5,0 |
| Body width | MOKOT | 63,2 | 64,1 | 4,9 | 7,7 | 52,7 | 71,6 | 12,0 |
|  | BOTAN | 61,1 | 60,0 | 7,8 | 12,7 | 55,4 | 74,4 | 5,0 |
| Body length:width, ratio | MOKOT | 1,0 | 1,0 | 0,1 | 6,2 | 0,9 | 1,1 | 12,0 |
|  | BOTAN | 1,0 | 1,0 | 0,1 | 11,9 | 0,8 | 1,2 | 5,0 |
| Anterior end to anterior end of adoral zone, distance | MOKOT | 38,0 | 36,2 | 5,2 | 13,6 | 31,4 | 45,5 | 8,0 |
| Anterior end to posterior end of adoral zone, distance | MOKOT | 48,6 | 48,3 | 4,2 | 8,7 | 43,1 | 54,1 | 8,0 |
| Posterior cell end to anterior end of adoral zone, distance | MOKOT | 39,2 | 27,2 | 19,4 | 49,5 | 19,5 | 65,8 | 13,0 |
| Posterior cell end to posterior end of adoral zone, distance | MOKOT | 32,7 | 15,7 | 24,6 | 75,2 | 11,7 | 65,8 | 13,0 |
| Posterior cell end to adoral zone distance, ratio | MOKOT | 0,7 | 0,6 | 0,2 | 29,7 | 0,5 | 1,0 | 13,0 |
| Anterior end to fragment 1 of epistomial fringe, distance | MOKOT | 12,6 | 12,8 | 4,5 | 35,2 | 5,4 | 20,2 | 13,0 |
|  | BOTAN | 18,0 | 18,0 | 2,6 | 14,3 | 15,1 | 21,5 | 5,0 |
| Longest membranelle, length | MOKOT | 12,1 | 12,1 | 1,5 | 12,4 | 9,6 | 13,8 | 8,0 |
| Macronucleus, number | MOKOT | 1,0 | 1,0 | 0,0 | 0,0 | 1,0 | 1,0 | 13,0 |
|  | BOTAN | 1,0 | 1,0 | 0,0 | 0,0 | 1,0 | 1,0 | 4,0 |
| Macronucleus, length | MOKOT | 17,8 | 18,0 | 2,6 | 14,5 | 11,8 | 21,5 | 13,0 |
|  | BOTAN | 15,8 | 14,8 | 2,5 | 16,0 | 14,0 | 19,5 | 4,0 |
| Macronucleus, width | MOKOT | 13,6 | 14,0 | 1,6 | 11,8 | 10,4 | 16,2 | 13,0 |
|  | BOTAN | 15,5 | 14,5 | 2,0 | 13,2 | 14,4 | 18,5 | 4,0 |
| Micronucleus, number | MOKOT | 1,0 | 1,0 | 0,0 | 0,0 | 1,0 | 1,0 | 11,0 |
|  | BOTAN | 1,0 | 1,0 | 0,0 | 0,0 | 1,0 | 1,0 | 3,0 |
| Micronucleus, diameter | MOKOT | 4,3 | 4,2 | 1,1 | 24,5 | 2,0 | 6,4 | 11,0 |
|  | BOTAN | 2,8 | 2,8 | 0,1 | 3,6 | 2,7 | 2,9 | 3,0 |
| Epistomial fringe spacer, distance | MOKOT | 24,6 | 24,9 | 4,0 | 16,3 | 16,9 | 30,3 | 13,0 |
|  | BOTAN | 16,6 | 16,7 | 3,6 | 21,9 | 12,6 | 20,2 | 4,0 |
| dFS: anterior end to the anterior end of the front-most pectinelle of left fragment of epistomial fringe, distance | MOKOT | 36,3 | 38,0 | 4,3 | 11,9 | 28,7 | 42,3 | 13,0 |
|  | BOTAN | 33,0 | 33,9 | 4,2 | 12,7 | 27,6 | 36,5 | 4,0 |
| Fringe spacer, ratio | MOKOT | 0,6 | 0,6 | 0,0 | 7,5 | 0,5 | 0,6 | 13,0 |
|  | BOTAN | 0,5 | 0,5 | 0,0 | 7,8 | 0,5 | 0,6 | 4,0 |
| dF2a: posterior end of the front-most pectinelle in left fragment of epistomial fringe to the posterior end, distance | MOKOT | 28,0 | 27,9 | 4,1 | 14,5 | 20,1 | 35,6 | 13,0 |
|  | BOTAN | 27,5 | 27,3 | 1,8 | 6,4 | 25,7 | 29,5 | 4,0 |
| dF2b: posterior end of the hind-most pectinelle in left fragment of epistomial fringe to the posterior end, distance | MOKOT | 20,7 | 20,2 | 2,5 | 12,0 | 16,6 | 25,5 | 13,0 |
|  | BOTAN | 20,2 | 19,7 | 2,0 | 9,9 | 18,5 | 23,1 | 4,0 |
| Posterior fringe ratio | MOKOT | 1,4 | 1,4 | 0,1 | 10,6 | 1,1 | 1,7 | 13,0 |
|  | BOTAN | 1,4 | 1,4 | 0,2 | 13,3 | 1,1 | 1,5 | 4,0 |
| Pectinelles of dextro frontal fragment of epistomial fringe, number | MOKOT | 21,2 | 21,0 | 0,4 | 2,1 | 21,0 | 22,0 | 13,0 |
|  | BOTAN | 21,2 | 21,0 | 0,4 | 1,9 | 21,0 | 22,0 | 6,0 |
| Pectinelles of left fragment of epistomial fringe, number | MOKOT | 9,0 | 9,0 | 0,6 | 6,4 | 8,0 | 10,0 | 13,0 |
|  | BOTAN | 8,6 | 9,0 | 0,5 | 6,4 | 8,0 | 9,0 | 5,0 |
| Adoral membranelles, number | MOKOT | 9,0 | 9,0 | 0,0 | 0,0 | 9,0 | 9,0 | 8,0 |
|  | BOTAN | 9,0 | 9,0 | 0,0 | 0,0 | 9,0 | 9,0 | 4,0 |
| Ciliary sockets of the perioral kinety, number | MOKOT | 24,7 | 25,0 | 3,8 | 15,5 | 20,0 | 34,0 | 11,0 |
|  | BOTAN | 24,4 | 25,0 | 1,9 | 8,0 | 21,0 | 26,0 | 5,0 |
| Dikinetids in inner endoral kinety, number | MOKOT | 11,5 | 12,0 | 2,3 | 20,3 | 8,0 | 15,0 | 11,0 |
|  | BOTAN | 8,8 | 8,5 | 1,0 | 11,1 | 8,0 | 10,0 | 6,0 |
| Dikinetids in outer endoral kinety, number | MOKOT | 13,4 | 14,0 | 1,9 | 14,2 | 10,0 | 15,0 | 10,0 |
|  | BOTAN | 10,8 | 10,0 | 1,1 | 10,1 | 10,0 | 12,0 | 5,0 |
| Supplementary ventral dikinetids, number | MOKOT | 2,0 | 2,0 | 0,0 | 0,0 | 2,0 | 2,0 | 3,0 |
|  | BOTAN | 2,0 | 2,0 | 0,0 | 0,0 | 2,0 | 2,0 | 4,0 |
| Dikinetids in ventral kinety 1, number | MOKOT | 16,0 | 16,0 | 1,9 | 12,0 | 13,0 | 19,0 | 7,0 |
|  | BOTAN | 14,2 | 14,5 | 1,0 | 6,9 | 13,0 | 15,0 | 6,0 |
| Dikinetids in ventral kinety 2, number | MOKOT | 20,0 | 20,0 | 1,2 | 6,0 | 18,0 | 22,0 | 12,0 |
|  | BOTAN | 16,8 | 17,0 | 0,4 | 2,4 | 16,0 | 17,0 | 6,0 |
| Rows of caudal cirri, number | MOKOT | 2,0 | 2,0 | 0,0 | 0,0 | 2,0 | 2,0 | 12,0 |
|  | BOTAN | 2,0 | 2,0 | 0,0 | 0,0 | 2,0 | 2,0 | 6,0 |

Measurements in µm based on protargol-impregnated specimens, except as noted (in vivo). M: mean, SD: Standard deviation, CV: coefficient of variation, Min: minimum value, Max: maximum value, N: number of specimens examined.
