# Supplementary material for: An extensive morphological and molecular characterization of the neglected class Odontostomatea (Ciliophora)
Source: Mar Life Sci Technol. 2026 Mar 12;8(2):289–323. doi: 10.1007/s42995-026-00352-x (PMC13198586; doi:10.1007/s42995-026-00352-x)
Supplement: Supplementary file 6 — Supplementary file6 (DOCX 20 KB) [file 42995_2026_352_MOESM6_ESM.docx]

**[Supplementary](sps:id::sec63) data**

Figure S1. Environmental phylogenetic tree of Odontostomatea. Labels in blue represent the obtained sequences in this study, and in green are the previously deposited sequences in GenBank.

Table S2. Biosamples used to extract the 18S rRNA sequences of environmental odontostomateans.

Table S3. Pebblescout summary of hits.

Table S4. Genetic distance matrix of the 18S rRNA sequences from the studied populations and published odontostomatean sequences.

Video S1. *Saprodinium mimeticum*. [[https://youtu.be/C9KLLl6s4gs](sps:urlprefix::https)](sps:urlprefix::https)

Video S2. *Saprodinium dentatum*. [[https://youtu.be/3aow_EjKkIU](sps:urlprefix::https)](sps:urlprefix::https)

Video S3. *Mircalla polidorii*. [[https://youtu.be/ZcgWQucdxgg](sps:urlprefix::https)](sps:urlprefix::https)

Video S4. *Epalxella* cf*. antiquorum*. [https://youtu.be/J5ghRbA8vKQ](sps:urlprefix::https)

Video S5. *Mylestoma monodontum*. [https://youtu.be/cDYyri9RtYg](sps:urlprefix::https)

Video S6. *Limnomylestoma shuriken*. [[https://youtu.be/XCUS5nZauNQ](sps:urlprefix::https)](sps:urlprefix::https)

Video S7. *Tostonella uncinata*. [[https://youtu.be/M63IUlVy5Ec](sps:urlprefix::https)](sps:urlprefix::https)

Video S8. *Discomorphella pectinata*. https://youtu.be/B-52wu8YgTM
