# Supplementary material for: An extensive morphological and molecular characterization of the neglected class Odontostomatea (Ciliophora)
Source: Mar Life Sci Technol. 2026 Mar 12;8(2):289–323. doi: 10.1007/s42995-026-00352-x (PMC13198586; doi:10.1007/s42995-026-00352-x)

Figure S1. Environmental phylogenetic tree of Odontostomatea. Labels in blue represent the obtained sequences in this study, and in green are the previously deposited sequences in GenBank.

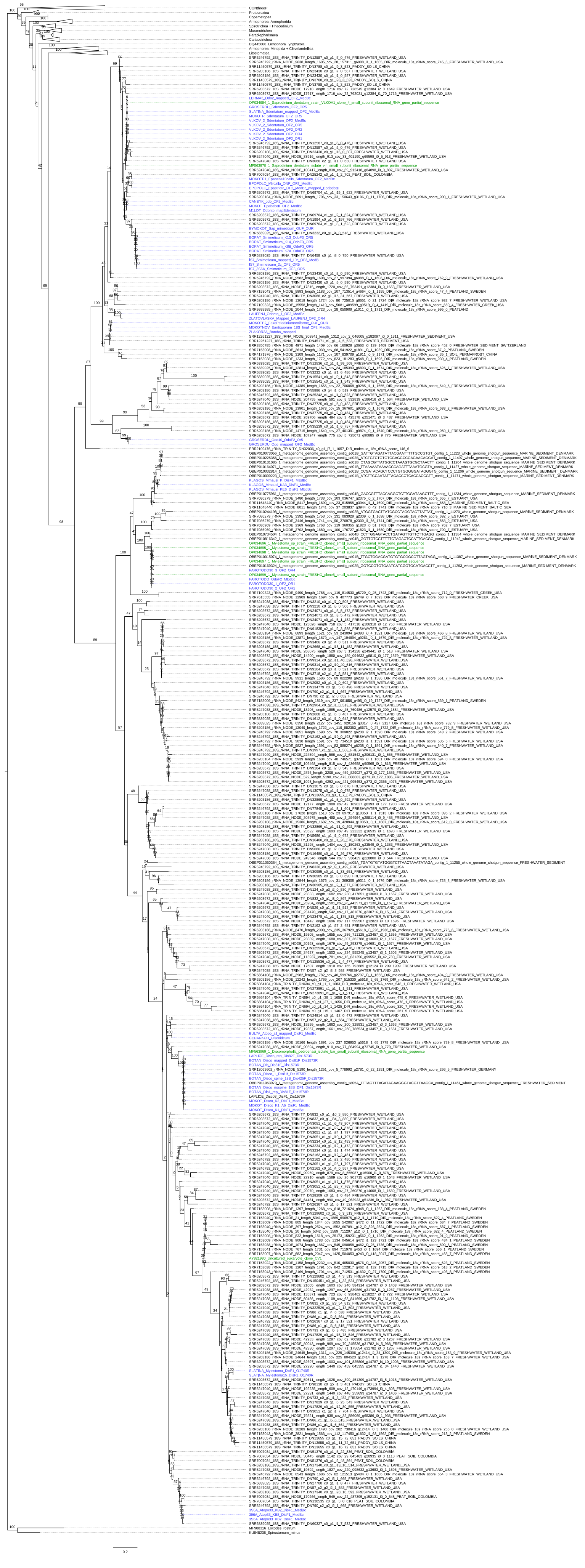

Supplement: Supplementary file 1 — Supplementary file1 (PDF 3808 KB) [file 42995_2026_352_MOESM1_ESM.pdf]
